# Unsupervised Feature Selection to Identify Important ICD-10 and ATC Codes for Machine Learning on a Cohort of Patients With Coronary Heart Disease: Retrospective Study

**DOI:** 10.2196/52896

**Published:** 2024-07-26

**Authors:** Peyman Ghasemi, Joon Lee

**Affiliations:** 1 Data Intelligence for Health Lab, Cumming School of Medicine, University of Calgary, Calgary, AB, Canada; 2 Department of Biomedical Engineering, University of Calgary, Calgary, AB, Canada; 3 Department of Cardiac Sciences, Cumming School of Medicine, University of Calgary, Calgary, AB, Canada; 4 Department of Community Health Sciences, Cumming School of Medicine, University of Calgary, Calgary, AB, Canada; 5 Department of Preventive Medicine, School of Medicine, Kyung Hee University, Seoul, Republic of Korea

**Keywords:** unsupervised feature selection, ICD-10, International Classification of Diseases, ATC, Anatomical Therapeutic Chemical, concrete autoencoder, Laplacian score, unsupervised feature selection for multicluster data, autoencoder-inspired unsupervised feature selection, principal feature analysis, machine learning, artificial intelligence, case study, coronary artery disease, artery disease, patient cohort, artery, mortality prediction, mortality, data set, interpretability, International Classification of Diseases, Tenth Revision

## Abstract

**Background:**

The application of machine learning in health care often necessitates the use of hierarchical codes such as the International Classification of Diseases (ICD) and Anatomical Therapeutic Chemical (ATC) systems. These codes classify diseases and medications, respectively, thereby forming extensive data dimensions. Unsupervised feature selection tackles the “curse of dimensionality” and helps to improve the accuracy and performance of supervised learning models by reducing the number of irrelevant or redundant features and avoiding overfitting. Techniques for unsupervised feature selection, such as filter, wrapper, and embedded methods, are implemented to select the most important features with the most intrinsic information. However, they face challenges due to the sheer volume of ICD and ATC codes and the hierarchical structures of these systems.

**Objective:**

The objective of this study was to compare several unsupervised feature selection methods for ICD and ATC code databases of patients with coronary artery disease in different aspects of performance and complexity and select the best set of features representing these patients.

**Methods:**

We compared several unsupervised feature selection methods for 2 ICD and 1 ATC code databases of 51,506 patients with coronary artery disease in Alberta, Canada. Specifically, we used the Laplacian score, unsupervised feature selection for multicluster data, autoencoder-inspired unsupervised feature selection, principal feature analysis, and concrete autoencoders with and without ICD or ATC tree weight adjustment to select the 100 best features from over 9000 ICD and 2000 ATC codes. We assessed the selected features based on their ability to reconstruct the initial feature space and predict 90-day mortality following discharge. We also compared the complexity of the selected features by mean code level in the ICD or ATC tree and the interpretability of the features in the mortality prediction task using Shapley analysis.

**Results:**

In feature space reconstruction and mortality prediction, the concrete autoencoder–based methods outperformed other techniques. Particularly, a weight-adjusted concrete autoencoder variant demonstrated improved reconstruction accuracy and significant predictive performance enhancement, confirmed by DeLong and McNemar tests (*P*<.05). Concrete autoencoders preferred more general codes, and they consistently reconstructed all features accurately. Additionally, features selected by weight-adjusted concrete autoencoders yielded higher Shapley values in mortality prediction than most alternatives.

**Conclusions:**

This study scrutinized 5 feature selection methods in ICD and ATC code data sets in an unsupervised context. Our findings underscore the superiority of the concrete autoencoder method in selecting salient features that represent the entire data set, offering a potential asset for subsequent machine learning research. We also present a novel weight adjustment approach for the concrete autoencoders specifically tailored for ICD and ATC code data sets to enhance the generalizability and interpretability of the selected features.

## Introduction

Machine learning is increasingly being used in health care to analyze patient data and provide insights on improving health outcomes and the quality of care [1]. With the rise of electronic health data (EHD) and entering a large amount of data per patient in hospitals, there are big opportunities to train machine learning models for a variety of applications, such as the prediction or diagnosis of diseases, outcome prediction, and treatment planning [1,2]. EHD are a valuable source of information on a patient, containing details on their demographics, hospital visits, medical diagnoses, physiological measurements, and treatments received [3]. However, despite the opportunities offered by these large data sets, there are challenges in terms of data quality, privacy, and the complexity of medical conditions [1]. In terms of machine learning, EHD can include many irrelevant and redundant features, where their direct use can lead to the “curse of dimensionality” as the high dimensionality of the data can make it more difficult to extract meaningful patterns and relationships [4]. Therefore, it is important to apply appropriate techniques for dimensionality reduction and feature engineering to address this challenge and improve the effectiveness of predictive models built from EHD.

Feature selection is one of the critical aspects of machine learning. It involves selecting a subset of relevant features that are the most useful for predicting a target variable. In the case of medical data, these features could include patient demographics, medical history, laboratory test results, and diagnosis codes [3]. Feature selection is essential because it can help improve the accuracy and performance of machine learning models by reducing the number of irrelevant or redundant features and avoiding overfitting [4]. Unsupervised feature selection is a type of feature selection method that is used when there is no target variable available to guide the selection of features. Unlike supervised feature selection, which chooses features that better predict a certain target variable, unsupervised feature selection methods rely on the intrinsic structure of the data to identify the most important features. This behavior helps the selected features to be unbiased and perform well when there are no labeled data. It can also reduce the risk of overfitting to a certain target variable and ensure robustness to new target variables [5]. This is an important advantage in health care, where collecting labeled data is usually difficult and the same data are often used to predict multiple target variables.

Generally, there are 3 main categories of feature selection methods: filter, wrapper, and embedded methods. Filter methods use statistical tests such as variance to rank individual features within a data set and select the features that maximize the desired criteria. However, they usually lack the ability to consider the interactions between features [6]. Wrapper methods, on the other hand, select features that optimize an objective function for a clustering algorithm. Therefore, these methods are generally specific to particular clustering algorithms and may not be suitable for use with other algorithms. Wrapper methods can detect potential relationships between features, but this often results in increased computational complexity [5]. Embedded methods also take into account feature relationships but generally do so more efficiently by incorporating feature selection into the learning phase of another algorithm. Lasso regularization is one of the well-known embedded methods that can be applied to a variety of machine learning models [6].

The *International Classification of Diseases, Tenth Revision* (*ICD-10*) is a method of classifying diseases that was created by the World Health Organization and is used internationally [7]. It categorizes diseases based on their underlying cause, characteristics, symptoms, and location in the body and uses codes to represent each disease. The *ICD-10* system organizes thousands of codes in a hierarchical structure that includes chapters, sections, categories, and expansion codes. Within this structure, section codes and their corresponding chapter codes can be thought of as child-parent relationships, with each *ICD-10* code serving as a node in the classification system. The same relationship applies to categories and sections, as well as expansion codes and categories. The high number of codes in this system is one of the major challenges of using them in machine learning applications [8]. It is worth noting that Canada has added or changed some codes in the lower levels according to their health care system requirements (*ICD-10, Canada* [*ICD-10-CA*]) [9].

Similar to International Classification of Diseases (ICD) codes, the Anatomical Therapeutic Chemical (ATC) classification system, developed by the World Health Organization Collaborating Centre for Drug Statistics Methodology, is an international tool for the active and systematic categorization of active pharmaceutical ingredients [10]. ATC codes are also structured hierarchically and are assigned based on the organ or system they impact, as well as their therapeutic, pharmacological, and chemical properties. This hierarchical system comprises 5 distinct levels, with the lower levels providing detailed information about the pharmacological subgroup and chemical substance, and the highest level representing the anatomical main group. As in the *ICD-10*, the ATC’s hierarchy introduces child-parent relationships at each level.

In this research, we used 3 administrative databases comprising *ICD-10* and ATC codes pertaining to patients with coronary artery disease (CAD). These databases, relevant to acute care, ambulatory care, and pharmacy facilities, were used to select the most insightful codes characterizing this cohort.

## Methods

### Data Set and Preprocessing

The Alberta Provincial Project for Outcome Assessment in Coronary Heart Disease (APPROACH) registry [11] is one of the most comprehensive data repositories of CAD management in the world, matching unique disease phenotypes with rich clinical information and relevant outcomes for patients in Alberta, Canada, who have undergone diagnostic cardiac catheterization or revascularization procedures. Our cohort’s patients were selected from the APPROACH registry. These patients underwent diagnostic angiography between January 2009 and March 2019 at 1 of the following 3 hospitals in Alberta: Foothills Medical Centre, University of Alberta Hospital, and Royal Alexandra Hospital. We excluded patients with ST elevation myocardial infarction from the study to focus on nonemergency CAD.

Discharge Abstract Database (DAD), National Ambulatory Care Reporting System (NACRS), and Pharmaceutical Information Network (PIN) data for the abovementioned patients were extracted from Alberta provincial health records. The DAD contains summary hospitalization information from all acute care facilities in Alberta. The NACRS includes all visits to ambulatory care facilities (ie, emergency department, urgent care, and day surgery visits) in the province as well as some nonabstracted data from other specialty clinics. The PIN is based on a system that collects all prescription medicine dispensations from pharmacies all over Alberta.

In the DAD and NACRS, for each patient, we aggregated all *ICD-10-CA* codes of hospital admissions or physician visits every 3 months following the first admission date (all codes in that period are treated as 1 record’s codes). This helps us to make sure that chronic diseases are captured more comprehensively in fewer records and reduce the effect of noisy records. We did a similar procedure for ATC codes in the PIN data set and aggregated the codes every 6 months, since most medications prescription refills did not extend beyond 6 months. We one-hot encoded the *ICD-10-CA* and ATC codes and their parent nodes for each record. For example, if the *ICD-10-CA* code “I251” was present, “I25,” “I20-I25,” and “Chapter IX” were also encoded in the one-hot table. Similarly, if the ATC code “C07AB02” was present, “C07AB,” “C07A,” “C07,” and “C” were also encoded. We show the number of all unique *ICD-10-CA* or ATC codes in the data set with *N_All_*.

To validate the performance of the selected features in a real clinical problem, we pulled the mortality data of the patients enrolled in the cohort from the Vital Statistics Database and matched them with the aggregated records to determine 90-day mortality following the end of the last procedure.

### Feature Selection

The following unsupervised algorithms were used for feature selection:

- Concrete autoencoder (CAE) [6]: In this method, continuous relaxation of discrete random (concrete) variables [12] and the Gumbel-Softmax reparameterization trick are used to construct a special layer in the neural network to transform discrete random variables into continuous ones, which allows for efficient computation and optimization using gradient-based methods. The reparameterization trick allows the use of a softmax function in this layer, which is differentiable, unlike the argmax function. This characteristic is useful for designing an autoencoder, in which features are selected in the concrete layer (as the encoder) through the softmax operation and a common neural network (as the decoder) is used to reconstruct the main feature space out of the selected features. During the training, a temperature parameter can be gradually decreased, allowing the concrete selector layer to try different features in the initial epochs and behave more similar to an argmax function in the last epochs to keep the best features. After training, we can use an argmax function on the weights to find the features passed to the neurons of the encoder layer. One of the major problems of this method is that it may converge to a solution where some duplicate features are selected in some neurons (ie, fewer than the desired number of features are selected).
- Autoencoder-inspired unsupervised feature selection (AEFS) [13]: This method combines autoencoder and group lasso tasks by applying an L_1,2_ regularization on the weights of the autoencoder. The autoencoder in this method tries to map the inputs to a latent space and then reconstruct the inputs from that space. The L_1,2_ regularization will optimize the weights (change them toward 0) to select a smaller number of features. The neural network structure of the autoencoder will enable the model to incorporate both linear and nonlinear behavior of the data in the results. After training this neural network, the features with higher weight values in the first layer can be selected as the most informative features. The authors claimed that this algorithm showed promising results in computer vision tasks.
- Principal feature analysis (PFA) [14]: This method selects features based on principal component analysis (PCA). The most important features are selected by applying a *k*-means clustering algorithm to the components of PCA and finding the features dominating each cluster (closest to the mean of the cluster). This algorithm is primarily designed for computer vision.
- Unsupervised feature selection for multicluster data (MCFS) [15]: This approach prioritizes the preservation of the multicluster structure of data. The algorithm involves constructing a nearest neighbor graph of the features, solving a sparse eigen-problem to find top eigenvectors with regard to the smallest eigenvalues. Then, an L_1_-regularized least squares problem is optimized to find the linear weights between the features and the eigenvectors. This allows us to define the MCFS score as the maximum weight of each feature across different clusters and select the highest scores as the best features.
- Laplacian score (LS) [16]: The LS algorithm uses the nearest neighbor graph to capture the local structure of the data in the affinity matrix. For each feature, its adjusted variation is calculated by removing the feature’s mean, normalized by a degree matrix, which itself is derived from the sum of similarities in the affinity matrix. The Laplacian matrix, essential for this calculation, is formed by subtracting the affinity matrix from the degree matrix. The significance of each feature is then assessed by the LS, which is the ratio of the feature’s ability to preserve local information (captured by its adjusted variation’s alignment with the Laplacian matrix) to its overall variance (measured by its alignment with the degree matrix). The lower the LS, the more relevant the feature for representing the intrinsic geometry of the data set.

We applied the LS, AEFS, PFA, MCFS, and CAE algorithms to a 67% training data set (split based on patients) of one-hot encoded features to select the best 100 features (*N_Best_*=100) with the following specifications (we chose *N_Best_* based on preliminary experimentations).

For the AEFS method, we used a single hidden-layer autoencoder and optimized the loss function as described in Han et al [13], with α=0.001 as the trade-off parameter of the reconstruction loss and the regularization term and β=0.1 as the penalty parameter for the weight decay regularization. The choice of these parameters was based on preliminary experimentations on a small set of data and exploring α and β of {0.001, 0.1, 1, 1000}.

For the PFA method, we used incremental PCA instead of the normal PCA in the original paper [14], with a batch size of 2*N_All_* due to the high computational cost. We decomposed the data to $\frac{N_{All}}{2}$ components and then applied *k*-means clustering to find *N_Best_* clusters. We also tried { $\frac{N_{All}}{5},\frac{N_{All}}{3},\frac{N_{All}}{2}$ } as the number of components of the PCA in the preliminary experiments.

To use the LS and MCFS methods for feature selection, we used the Euclidean distances between features to construct a nearest neighbor graph G based on the 5 nearest neighbors. For the LS method, we set the weights of the connected nodes of G to 1, assuming a large *t* in the LS formulation. Then, we computed the LS for each feature and selected the top features with higher scores. Due to the high computational resources required for the LS and MCFS methods, we did not explore different parameters and used the same settings suggested by the implementation codes of these algorithms.

As the structure of the loss function allows us to prioritize some target variables, the CAE method was applied in 2 different ways—with and without adjusting weights for features. The reason for adjusting the weights is that since there are many correlated features in the *ICD-10-CA* and ATC code data sets, the model may choose one of them randomly [3]. Therefore, we applied the function in equation 1 as the class weights of the features to the loss function of the model:


(1) $$W_{F}=\frac{1}{1+d\left(F\right)}$$

where *W_F_* is the weight for feature *F* and *d*(*F*) is the depth of feature *F* as a node of the *ICD-10-CA* or ATC tree. This weight adjustment will force the model to give more importance to the features at the top of the tree and to generalize more in clinical settings. In the rest of the paper, this variant of the CAE model will be referred to as the CAE with weight adjustment (CAEWW) and the regular CAE model will be referred to as the CAE with no weight adjustment (CAENW).

We defined *N_Best_* neurons in the concrete selector layer and used a learning rate of 0.001, a batch size of 64, and 1000 epochs. We also controlled the learning of the concrete selector layer by the temperature parameter that started from 20 and decreased to 0.01 exponentially (this annealing strategy was suggested by Abid et al [6] for better convergence). The decoder of the CAE was a feed-forward neural network with 2 hidden layers with 64 neurons and used a sigmoid activation function for the output layer and a leaky rectified linear unit activation function for the other layers. The learning rate, number of neurons, and the layers were determined based on preliminary experiments for the fastest convergence of the autoencoder.

### Evaluation of Selected Features: Reconstruction of Initial Feature Space

To evaluate the effectiveness of the selected features, we trained a simple feed-forward neural network model using the chosen features to reconstruct the original feature space for each data set separately. The neural network consisted of 2 hidden layers, each with 64 neurons, and used leaky rectified linear unit activation functions, with a 10% dropout rate, in the hidden layers and a sigmoid activation function in the output layer. We trained the model using the same training set used in the feature selection step and evaluated its performance on the remaining 33% test set using binary cross entropy. We also calculated the accuracy of each feature selection method to determine which method produced the most accurate results. One of the challenges in comparing models with a large number of targets is that the accuracy values are inflated, because most of the targets are heavily imbalanced (ie, most of them were 0s) and the models were able to predict them easily. To circumvent this issue, we used a 2-tailed *t* test analysis and compared the accuracy values of the classes with the accuracy of a baseline model that simply outputs the mode of the training data for each class regardless of the input.

### Evaluation of Selected Features: Prediction of 90-Day Mortality

To demonstrate the utility of using unsupervised feature selection methods in a supervised setting, we conducted a case study where we used the selected features from each method to predict 90-day mortality following the end of the last procedure for each data set separately. Since our data sets were highly imbalanced, with only ~6%, ~2%, and ~1% of the aggregated records for the DAD, NACRS, and PIN data set, respectively, leading to 90-day mortality, we upsampled the minority class using random sampling to balance the training sets. We then trained extreme gradient boosting (XGBoost) models using the training sets with 5-fold cross-validation to tune the hyperparameters for each model. We used the best models to predict the binary outcome variables on the test sets and measured their performances. XGBoost was selected for its efficiency with sparse data, which was crucial for our data sets. XGBoost’s regularization features help prevent overfitting [17]. Additionally, its ability to provide interpretable models through tree-based Shapley values aligns with our objective to not only predict mortality but also understand the contributing factors [18]. XGBoost’s scalability on multiple processors and speed (for both training [17] and Shapley analysis [18]) are also beneficial for processing large volumes of data and complex model tuning. After training the mortality prediction models for each method and data set, we calculated tree-based Shapley values corresponding to the features. This allowed us to rank the importance of each feature and explain their roles in predicting mortality.

We have made the implementation code for the methods discussed available at our GitHub repository [19].

### Ethical Considerations

This study received ethics approval from the Conjoint Health Research Ethics Board at the University of Calgary (REB20-1879). Informed consent was waived due to the retrospective nature of the data and the large number of patients involved, making it impractical to seek consent from each patient. All data were deidentified. No compensation was provided to the participants as the study did not involve direct participant interaction.

## Results

### Data Set Description

Table 1 summarizes the characteristics of the patients in the cohort at the time of their initial catheterization. The total numbers of patients with at least 1 record in the respective data sets, as well as the time ranges for each data set, are provided in Table 2. The aggregation procedure described in the *Methods* section reduced the number of records to the values listed in the “Aggregated Records” row, and the table also includes the total number of codes (unique *ICD-10-CA* or ATC codes and their parent codes) in a data set, along with the average number of codes per record. Multimedia Appendix 1 illustrates the percentages of the 20 most common *ICD-10-CA* and ATC codes within each processed data set. Within the data set, there were 9942 cases corresponding to a 90-day mortality, resulting in a 20% mortality rate in the cohort. The final aggregated data for each data set were split into 67% for the training sets and 33% for the test sets at the patient level.

**Table 1.** Key characteristics of the patients with CAD^a^ enrolled in the cohort.

Variable		Overall (N=51,506)
Total population, n (%)		51,506 (100)
**Sex, n (%)**		
	Female	12,875 (25)
	Male	38,631 (75)
Age (years), mean (SD)		66.09 (11.41)
**BMI (kg/m** ^**2**^ **), mean (SD)**		29.51 (7.45)
	Missing data, n (%)	8449 (16.4)
**CAD type, n (%)**		
	Non-ST elevation myocardial infarction	24,119 (46.83)
	Unstable angina	10,671 (20.72)
	Stable angina	9832 (19.09)
	Missing data	6884.0 (13.37)
**Canadian Cardiovascular Society angina grade, n (%)**		
	II (slight limit)	4688 (9.1)
	IVb	7513 (14.59)
	IVa (hospitalized with acute coronary syndrome)	21,117 (41)
	III (marked limit)	2581 (5.01)
	IVc	1627 (3.16)
	I (strenuous)	1309 (2.54)
	Atypical	698 (1.36)
	Other or missing data	11,973 (23.25)
**Diabetes, n (%)**		
	No diabetes	37,544 (72.89)
	Type II	12,067 (23.43)
	Type I	806 (1.56)
	Other	1089 (2.11)
Dyslipidemia, n (%)		32,967 (64.01)
Heart failure, n (%)		3689 (7.16)
Atrial fibrillation or flutter, n (%)		1220 (2.37)
Hypertension, n (%)		32,264 (62.64)
Angina, n (%)		2559 (4.97)
Family history of CAD, n (%)		15,209 (29.53)
**Smoking, n (%)**		
	Never	25,822 (50.13)
	Current	11,196 (21.74)
	Past	14,488 (28.13)
Chronic lung disease, n (%)		5318 (10.33)
Cerebrovascular disease, n (%)		2040 (3.96)
Psychiatric history, n (%)		1097 (2.13)
Venous insufficiency, n (%)		476 (0.92)
Alcohol consumption, n (%)		599 (1.16)
**Extent of CAD, n (%)**		
	3 VDs^b^	247 (0.48)
	3 VDs (one >75%)	7765 (15.08)
	3 VDs (>75% proximal LAD^c^)	5704 (11.07)
	3 VDs (proximal LAD)	3318 (6.44)
	2 VDs	5392 (10.47)
	2 VDs (>75% LAD)	569 (1.1)
	2 VDs (both >75%)	5215 (10.13)
	2 VDs (>75% proximal LAD)	2819 (5.47)
	1 VD (>75% proximal LAD)	2299 (4.46)
	1 VD (>75%)	8504 (16.51)
	1 VD (50%‐75%)	4032 (7.83)
	Severe left main disease	3058 (5.94)
	Left main disease	2584 (5.02)

a CAD: coronary artery disease.

b VD: vessel disease.

c LAD: left anterior descending.

**Table 2.** Summary statistics of the DAD^a^, NACRS^b^, and PIN^c^ data sets.

Summary statistics	Data set		
	DAD	NACRS	PIN
Patients with at least 1 record, n	49,075	50,628	49,052
Records, n	273,910	3,974,403	28,807,136
Aggregated records, n	166,083	173,507	997,997
Unique *ICD-10-CA^d^* or ATC^e^ codes and their parent codes, n	9651	7803	2315
Codes per aggregated record, mean (SD)	24.90 (16.55)	15.27 (12.55)	33.31 (18.95)
Time range	2004‐2022	2010‐2022	2004‐2022

a DAD: Discharge Abstract Database.

b NACRS: National Ambulatory Care Reporting System.

c PIN: Pharmaceutical Information Network.

d *ICD-10-CA*: *International Classification of Diseases, Tenth Revision, Canada*.

e ATC: Anatomical Therapeutic Chemical.

### Performances of the Feature Selection Methods

Table 3 shows the accuracies and binary cross entropies of the models based on the selected features from each method. Table 4 shows the accuracy, *F*_1_-score, and area under the receiver operating characteristic curve (AUC-ROC) metrics of the XGBoost models to predict 90-day mortality.

**Table 3.** Average accuracy and binary cross entropy (BCE) loss of different sets of selected features in reconstructing the original feature space in a neural network structure.

Feature selection method	DAD^a^		NACRS^b^		PIN^c^	
	Accuracy, mean (95% CI)	BCE, mean (95% CI)	Accuracy, mean (95% CI)	BCE, mean (95% CI)	Accuracy, mean (95% CI)	BCE, mean (95% CI)
CAEWW^d^	0.9992^e^ (0.9992-0.9993)	0.0121^e^ (0.0121-0.0121)	0.9994^e^ (0.9994-0.9995)	0.0091^e^ (0.0091-0.0091)	0.9972^e^ (0.9969-0.9975)	0.0432^e^ (0.0432-0.0432)
CAENW^f^	0.9992^e^ (0.9991-0.9993)	0.0121^e^ (0.0121-0.0121)	0.9994^e^ (0.9993-0.9994)	0.0094^e^ (0.0094-0.0094)	0.9972^e^ (0.9969-0.9974)	0.0438^e^ (0.0438-0.0438)
AEFS^g^	0.9976 (0.9972-0.9980)	0.0370 (0.0370-0.0370)	0.9982 (0.9979-0.9985)	0.0274 (0.0274-0.0274)	0.9884 (0.9867-0.9901)	0.1794 (0.1794-0.1794)
MCFS^h^	0.9991^e^ (0.9990-0.9991)	0.0145^e^ (0.0145-0.0145)	0.9992^e^ (0.9992-0.9993)	0.0117^e^ (0.0117-0.0117)	0.9956^e^ (0.9951-0.9962)	0.0677^e^ (0.0677-0.0677)
PFA^i^	0.9975 (0.9971-0.9979)	0.0382 (0.0382-0.0382)	0.9981 (0.9978-0.9985)	0.0286 (0.0286-0.0286)	0.9871 (0.9852-0.9891)	0.1982 (0.1982-0.1982)
LS^j^	0.9989^e^ (0.9988-0.9990)	0.0165^e^ (0.0165-0.0165)	0.9991^e^ (0.9990-0.9992)	0.0136^e^ (0.0136-0.0136)	0.9945^e^ (0.9938-0.9952)	0.0850^e^ (0.0850-0.0850)
*Mode of the training set (baseline model*)	0.9975 (0.9971-0.9979)	0.0384 (0.0322-0.0447)	0.9981 (0.9978-0.9984)	0.0294 (0.0245-0.0342)	0.9870 (0.9850-0.9889)	0.2012 (0.1712-0.2312)

a DAD: Discharge Abstract Database.

b NACRS: National Ambulatory Care Reporting System.

c PIN: Pharmaceutical Information Network.

d CAEWW: concrete autoencoder with weight adjustment.

e Significantly different from the baseline model that outputs the mode of each class (*P*<.05). The *P* values are presented in Table S1 of Multimedia Appendix 2.

f CAENW: concrete autoencoder with no weight adjustment.

g AEFS: autoencoder-inspired unsupervised feature selection.

h MCFS: unsupervised feature selection for multicluster data.

i PFA: principal feature analysis.

j LS: Laplacian score.

**Table 4.** Performance of the extreme gradient boosting (XGBoost) model in predicting 90-day mortality using different sets of selected features.

Feature selection method	DAD^a^			NACRS^b^			PIN^c^		
	Accuracy	*F*_1_-score	AUC-ROC^d^	Accuracy	*F*_1_-score	AUC-ROC	Accuracy	*F*_1_-score	AUC-ROC
CAEWW^e^	0.86	0.37	0.87	0.85	0.15	0.75	0.84	0.1	0.82
CAENW^f^	0.86	0.36	0.87^g^	0.86	0.15	0.75	0.83	0.1	0.82
AEFS^h^	0.88	0.21	0.61^g^	0.9	0.09	0.56^g^	0.85	0.08	0.69^g^
MCFS^i^	0.86	0.37	0.89^g^	0.84	0.13	0.74	0.81	0.09	0.84^g^
PFA^j^	0.92	0.05	0.5^g^	0.97	0.02	0.5^g^	0.93	0.05	0.54^g^
LS^k^	0.77	0.26	0.81^g^	0.8	0.11	0.73^g^	0.76	0.08	0.82

a DAD: Discharge Abstract Database.

b NACRS: National Ambulatory Care Reporting System.

c PIN: Pharmaceutical Information Network.

d AUC-ROC: area under the receiver operating characteristic curve.

e CAEWW: concrete autoencoder with weight adjustment.

f CAENW: concrete autoencoder with no weight adjustment.

g Significantly different from the AUC-ROC of the model trained on CAEWW features in their corresponding data set (*P*<.05) using the DeLong test [20]. The *P* values are presented in Table S2 in Multimedia Appendix 2.

h AEFS: autoencoder-inspired unsupervised feature selection.

i MCFS: unsupervised feature selection for multicluster data.

j PFA: principal feature analysis.

k LS: Laplacian score.

Both tables indicate that the CAE methods generally selected superior features compared to the other algorithms. Adjusting the weights within the CAE improved the performance of feature space reconstruction slightly. In terms of predicting 90-day mortality, the CAE methods again performed better than the other methods, as evidenced by the AUC-ROC. This superior performance was statistically significant in most instances (*P*<.05), according to the DeLong test for AUC-ROC. Furthermore, the McNemar test revealed a significant difference between the overall performance of the mortality prediction models trained on the features of the CAEWW method and those based on the other methods (*P*<.05). The *P* values of the McNemar and DeLong tests can be found in Table S2 of Multimedia Appendix 2.

**Figure 1.**
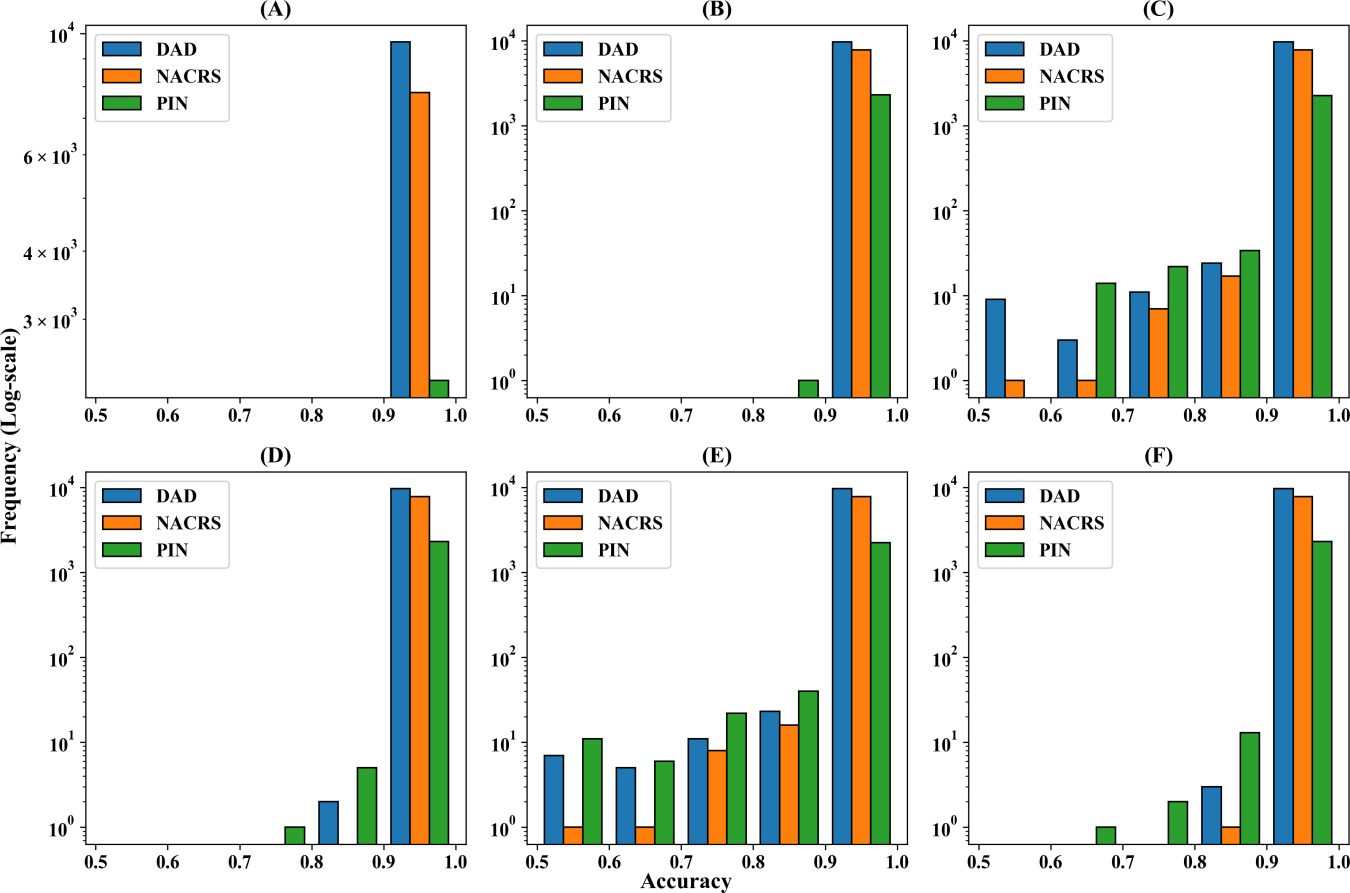
Log-scale histograms of the initial feature space reconstruction accuracy in each International Classification of Diseases (ICD) code for different feature selection methods and different data sets: (A) concrete autoencoder with weight adjustment (CAEWW), (B) concrete autoencoder with no weight adjustment (CAENW), (C) autoencoder-inspired unsupervised feature selection (AEFS), (D) unsupervised feature selection for multicluster data (MCFS), (E) principal feature analysis (PFA), and (F) Laplacian score (LS). DAD: Discharge Abstract Database; NACRS: National Ambulatory Care Reporting System; PIN: Pharmaceutical Information Network.

Figure 1 shows the log-scale histograms of the original feature space reconstruction accuracy in each *ICD-10-CA* or ATC code for different feature selection methods. It shows that CAEWW and CAENW were the best methods in terms of reconstructing the majority of the features with high accuracy. The other methods, despite having high average accuracy, performed poorly in reconstructing some of the features.

### Characteristics of the Selected Features

**Figure 2.**
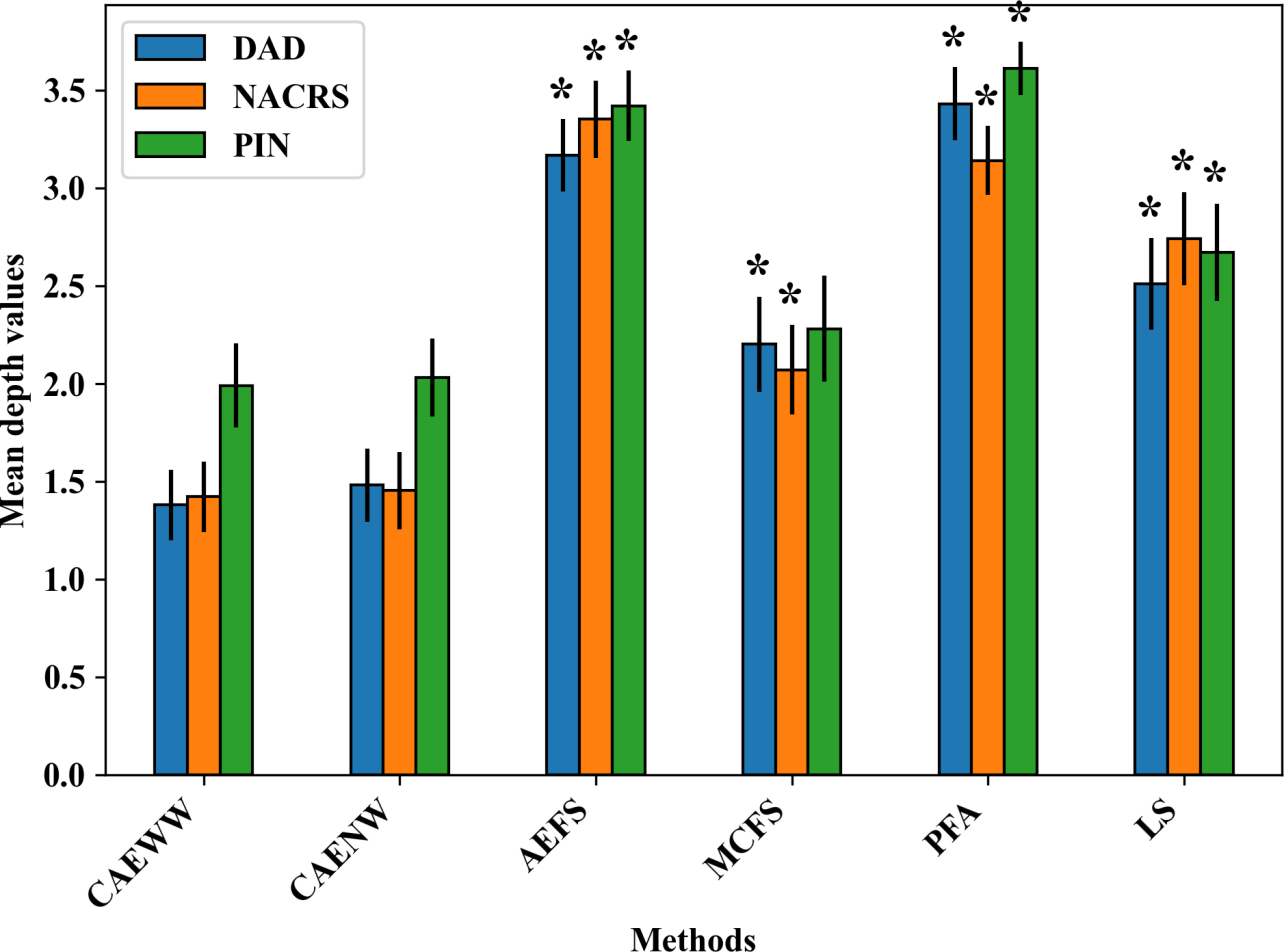
Average depths of the selected codes by each method in the *ICD-10-CA* or ATC tree. Methods with average depths significantly (*P*<.05) larger than the CAEWW method in their corresponding data set are marked with asterisks (*). AEFS: autoencoder-inspired unsupervised feature selection; ATC: Anatomical Therapeutic Chemical CAENW: concrete autoencoder with no weight adjustment; CAEWW: concrete autoencoder with weight adjustment; DAD: Discharge Abstract Database; *ICD-10-CA*: *International Classification of Diseases, Tenth Revision, Canada*; LS: Laplacian score; MCFS: unsupervised feature selection for multicluster data; NACRS: National Ambulatory Care Reporting System; PFA: principal feature analysis; PIN: Pharmaceutical Information Network.

We also calculated the average depths of the codes selected by each method and compared them (using 2-tailed *t* test analysis) against the CAEWW method that is intended to select more general codes (ie, smaller depths). The CAEWW method selected codes with average depths of 1.38, 1.42, and 1.99 for the DAD, NACRS, and PIN data sets, respectively. Although CAEWW’s code depths were significantly lower than the depths of the codes selected by the other methods (all *P*<.05), there was no significant difference between the CAEWW and CAENW methods (with average depths of 1.48, 1.45, 2.03; all *P*>.05). The *P* values can be found in Table S3 of Multimedia Appendix 2. Figure 2 illustrates the difference in average code depth among the different methods.

**Figure 3.**
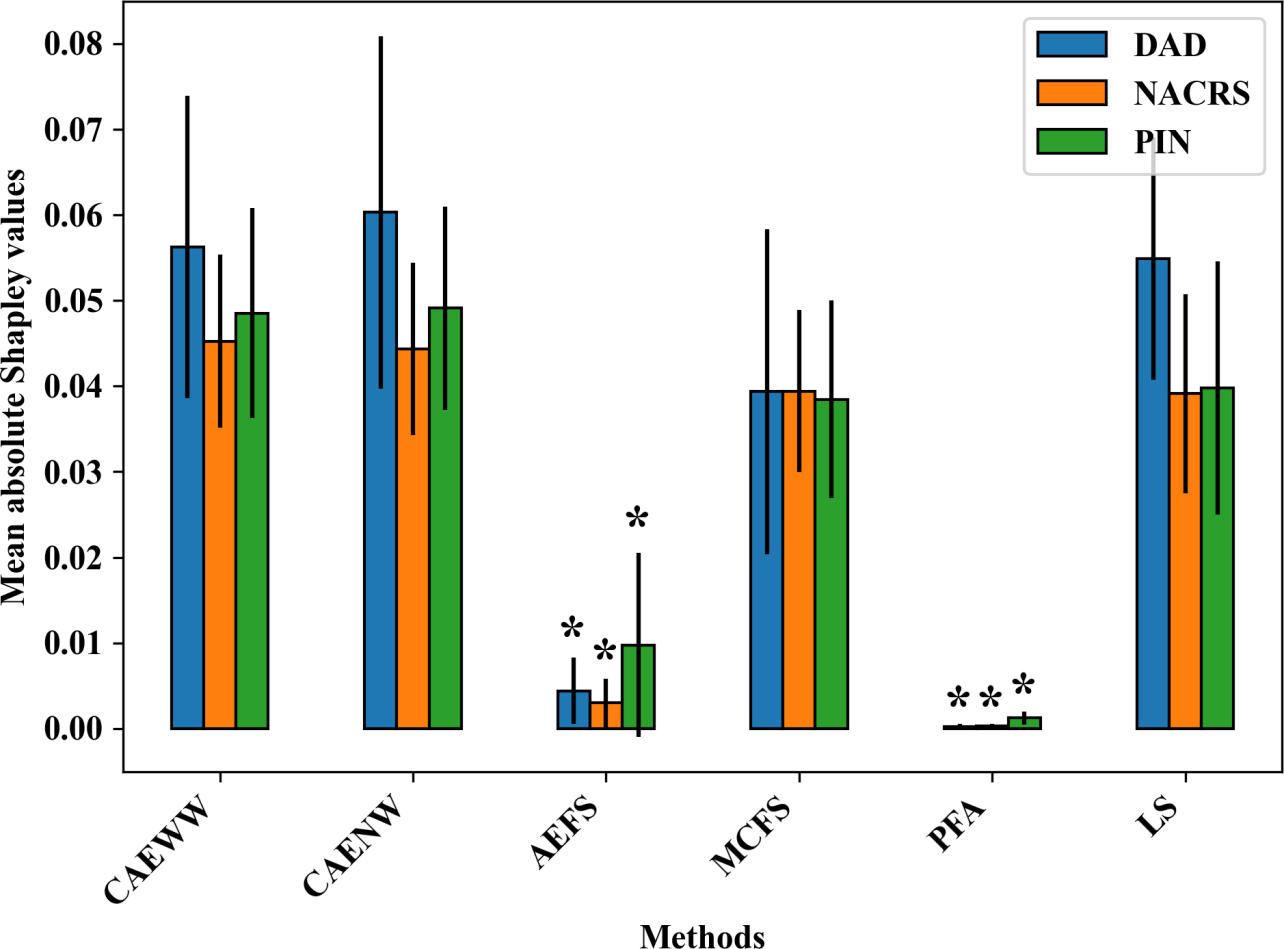
Average of mean absolute Shapley values of features in each mortality prediction model. Methods with average values significantly (*P*<.05) smaller than the CAEWW method in their corresponding data set are marked with asterisks (*). AEFS: autoencoder-inspired unsupervised feature selection; CAENW: concrete autoencoder with no weight adjustment; CAEWW: concrete autoencoder with weight adjustment; DAD: Discharge Abstract Database; LS: Laplacian score; MCFS: unsupervised feature selection for multicluster data; NACRS: National Ambulatory Care Reporting System; PFA: principal feature analysis; PIN: Pharmaceutical Information Network.

**Figure 4.**
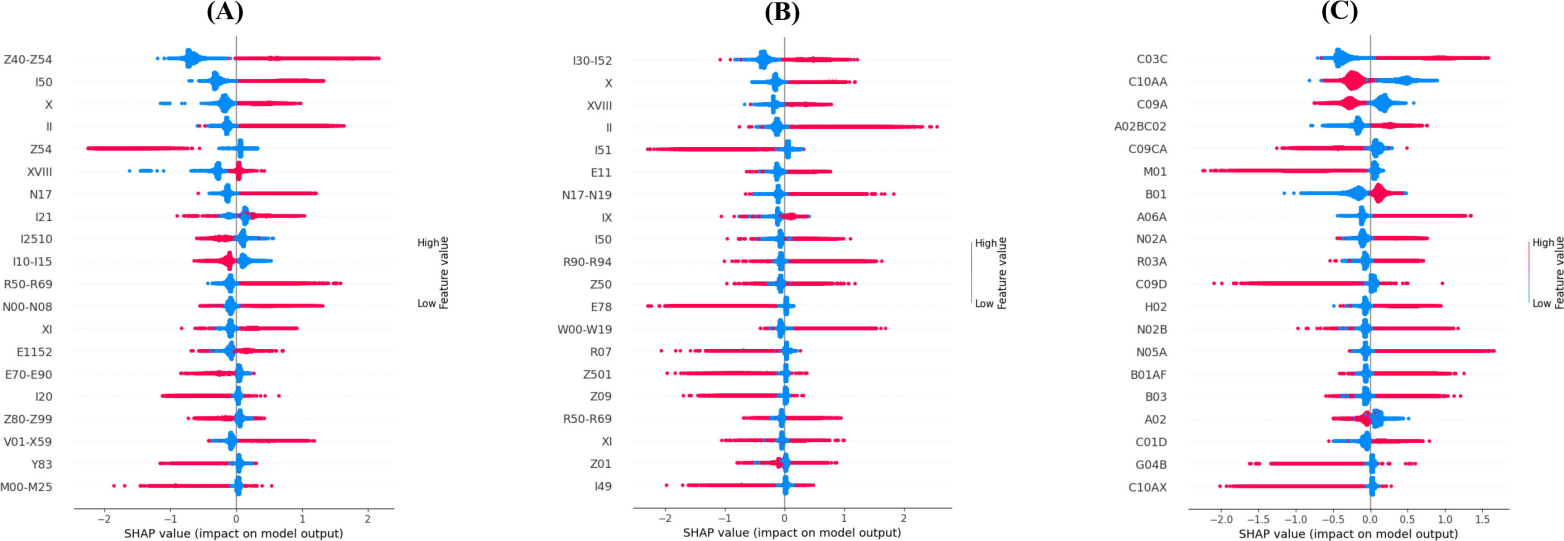
SHAP values of the features selected by the CAEWW method across different data sets (20 most important features): (A) Discharge Abstract Database (DAD), (B) National Ambulatory Care Reporting System (NACRS), and (C) Pharmaceutical Information Network (PIN). CAEWW: concrete autoencoder with weight adjustment; SHAP: Shapley additive explanations.

We used the average of the mean absolute Shapley values from each mortality prediction model as an index for the importance of the features selected by each method. To compare the CAEWW method with the other methods across the 3 different data sets, we conducted a 2-tailed *t* test analysis. The CAEWW method did not show any significant difference from the CAENW methods across all data sets (all *P*>.05). However, the CAEWW method did yield significantly higher mean absolute Shapley values compared to the other methods (AEFS and PFA; all *P*<.001). The AEFS and PFA methods had the lowest Shapley values compared to all the other methods, indicating that they selected lower-quality features for this task. The *P* values are available in Table S4 in Multimedia Appendix 2. Figure 3 illustrates the aforementioned differences in mean absolute Shapley values. Figure 4 shows the Shapley plots of the 20 most important features selected by the CAEWW method across different data sets. The corresponding Shapley plots for the other methods can be found in Multimedia Appendix 3 (Figures S1-S5). Additionally, Multimedia Appendix 4 (Tables S1-S18) includes all chosen features, detailed descriptions, and the average absolute Shapley values across all data sets and methods.

## Discussion

### Principal Findings

The high dimensionality of the ICD and ATC code databases necessitates the use of dimensionality reduction techniques to feed the data into machine learning models. Due to interpretability concerns in the health domain, selecting from original features, rather than transforming them into new features, is an essential step in reducing dimensionality. In this study, we demonstrated that the CAE methods performed the best in selecting the most informative ICD and ATC codes in an unsupervised setting. Using a clinical outcome as a case study, we also demonstrated that ICD and ATC code features selected by the CAE methods were able to predict the outcome variable with better accuracy than the other methods in the study, even though they were derived from an unsupervised setting in the absence of the target variable. This indicates that the selected features can be considered unbiased toward a specific target variable and explain the phenomenon appropriately. We also showed that the AEFS and PFA methods did not select high-quality features in our data set and were not suitable for both tasks of reconstructing the feature space and predicting 90-day mortality. The LS and MCFS methods, however, showed better performance in both tasks (slightly lower than the CAE methods). Furthermore, the features selected by the CAE methods (especially CAEWW) were generally higher-level codes (ie, lower depth in the hierarchical structure), which helps the study to find generalized solutions.

It is worth mentioning that our methodology code is publicly shared, allowing other researchers to use the desired methods for selecting the most informative features within cohorts with large ICD, ATC, or other hierarchical-coded health databases [19].

### Computational Cost

The MCFS, PFA, and LS methods had multiple special matrix operations that made them computationally expensive. Considering our large-scale, high-dimensional data set, these algorithms were not possible to run on a personal computer and we had to optimize the operations for an advanced computing cluster with 40 Intel Xeon Gold 6342 2.80 GHz CPUs and 2048 GB RAM. The MCFS and LS feature selection experiments had some shared operations and together took over 2 days to complete. The PFA method also needed less than a day for the entire feature selection experiments. The AEFS and CAE methods, however, had the advantage of using GPUs and optimized deep learning libraries for training the neural networks and were faster. Each of these algorithms took less than 4 hours on an Nvidia A100 80 GB GPU.

### Selected Features

The Shapely analysis of the 20 most important features selected in each data set using the CAEWW method for predicting mortality revealed the multidimensional capabilities of this method in identifying relevant information. In the DAD and NACRS data sets, it selected disease codes relevant to mortality among patients with CAD. In both data sets, diseases related to cardiovascular conditions, hypertensive and circulatory disorders, metabolic disorders, renal failures [21], and cancer [22] were selected, which are important factors in the outcomes of patients with CAD. Furthermore, DAD-based features included accidents, arthropathies, and hospitalization-specific conditions, whereas the NACRS data set resulted in falls [23]; digestive disorders [24]; and codes related to the rehabilitations, management, or complexity of the diseases. In the PIN data set, direct interventions for CAD and related risk factors were mainly selected, including high-ceiling diuretics, statins, ace inhibitors, angiotensin II receptor blockers (plain and combinations), direct factor Xa inhibitors, vasodilators, antianemic preparations, antithrombotic agents, and other lipid-modifying agents, addressing heart failure, cholesterol management, blood pressure control, anticoagulation, anemia, and blood flow. Also, drugs related to accompanying diseases or conditions with CAD were selected: gastrointestinal issues (pantoprazole and general drugs for acid-related disorders [25,26] and drugs for constipation [27]), pain management (opioids, other analgesics, and antipyretics) [28], inflammatory conditions and immune responses (anti-inflammatory and antirheumatic products and corticosteroids for systemic use) [29,30], mental and behavioral health (antipsychotics) [31], respiratory conditions (adrenergic inhalants [32]), and urological issues [33].

Previous studies typically selected codes as machine learning model features based on expert opinions, the presence of high-level codes (eg, categories or chapters), or a combination of both [34,35]. To the best of our knowledge, only 1 study [3] attempted to offer a sophisticated feature selection method using tree-lasso regularization for ICD code data sets, but it was in a supervised setting that required an outcome variable. Our study provides a general tool for health researchers to select the most informative ICD and ATC codes without biasing the study toward a specific outcome variable. We also introduced a unique target weight adjustment function to the CAE model to guide the model to select higher levels of the ICD table compared to the model without adjustment.

### Limitations and Future Work

One of the limitations of this study was the incapability of the CAE method to select an exact number of desired features. Since the neurons in the concrete selector layer work independently, there is a possibility of selecting duplicate features. Therefore, the number of final selected features can be fewer than the desired number. Although it indicates that the decoder model is still capable of reconstructing the initial feature space with a smaller number of features, some researchers may prefer to have an exact number of features they desire for their models. One previous study [36] has used a special regularization term in the training step to enforce the model not to select duplicate features. This method can be investigated for the ICD and ATC codes in the future.

The aggregation of codes should be viewed as a trade-off in this study. We needed to select a reasonable aggregation period that covers both long-term and short-term diseases. A shorter period could skew the results by including multiple correlated records from the same patient. Conversely, longer periods could weigh short-term diseases equally with long-term ones, and the codes of the patients with fewer records (eg, recent patients in the cohort) would have a lower chance of selection.

Another limitation was that we only used 3 data sets of a specific disease cohort to choose the features. Therefore, the selected features in this study may not generalize to other patient cohorts or diseases. Furthermore, we selected the 100 best features, but other data sets or patient cohorts may require a different number of features. Future studies may investigate the impact of the number of features on the results. Moreover, our hyperparameter analysis was conducted within a constrained scope due to limited computational resources. Future studies could further explore the impact of a broader range of hyperparameter values. We anticipate that the CAEs hold potential for this area due to their flexible neural network structure and optimized algorithms. A similar limitation also applies to the mortality prediction case study, where we only trained XGBoost and did not explore other model types.

### Conclusions

In this study, we investigated 5 different methods for selecting the best features in ICD and ATC code data sets in an unsupervised setting. We demonstrated that the CAE method can select better features representing the whole data set that can be useful in further machine learning studies. We also introduced weight adjustment of the CAE method for ICD and ATC code data sets that can be useful in the generalizability and interpretability of the models, given that it prioritizes selecting high-level definitions of diseases.

The CAEWW method outperformed all other methods in reconstructing the initial feature space across all data sets. We validated the selected features through a supervised learning task, predicting 90-day mortality after discharge using 3 distinct data sets. Features selected via the CAEWW method demonstrated significantly improved performance on this task, as evidenced by the DeLong and McNemar tests. Given the advantages of the CAE method, we recommend its use in the feature selection phase of EHD analysis with ICD or ATC codes.

## Supplementary material

10.2196/52896 Multimedia Appendix 1 The percentages of the 20 most common *ICD-10-CA* and ATC codes present in the processed data sets. ATC: Anatomical Therapeutic Chemical; *ICD-10-CA*: *International Classification of Diseases, Tenth Revision, Canada*.

10.2196/52896 Multimedia Appendix 2 *P* values associated with Tables3  4, and characteristics of the selected features.

10.2196/52896 Multimedia Appendix 3 Shapley value plots of the features selected by our methods across the different data sets (20 most important features).

10.2196/52896 Multimedia Appendix 4 Tables of the features selected by our methods across the different data sets with each feature’s description, rank, and average absolute Shapley score.

## Abbreviations

AEFS: autoencoder-inspired unsupervised feature selection
APPROACH: Alberta Provincial Project for Outcome Assessment in Coronary Heart Disease
ATC: Anatomical Therapeutic Chemical
AUC-ROC: area under the receiver operating characteristic curve
CAD: coronary artery disease
CAE: concrete autoencoder
CAENW: concrete autoencoder with no weight adjustment
CAEWW: concrete autoencoder with weight adjustment
DAD: Discharge Abstract Database
EHD: electronic health data
ICD: International Classification of Diseases
ICD-10: International Classification of Diseases, Tenth Revision
ICD-10-CA: International Classification of Diseases, Tenth Revision, Canada
LS: Laplacian score
MCFS: unsupervised feature selection for multicluster data
NACRS: National Ambulatory Care Reporting System
PCA: principal component analysis
PFA: principal feature analysis
PIN: Pharmaceutical Information Network
XGBoost: extreme gradient boosting
