# Supplementary figures and images for: Unsupervised Feature Selection to Identify Important ICD-10 and ATC Codes for Machine Learning on a Cohort of Patients With Coronary Heart Disease: Retrospective Study
Source: JMIR Med Inform. 2024 Jul 26;12:e52896. doi: 10.2196/52896 (PMC11295113; doi:10.2196/52896)

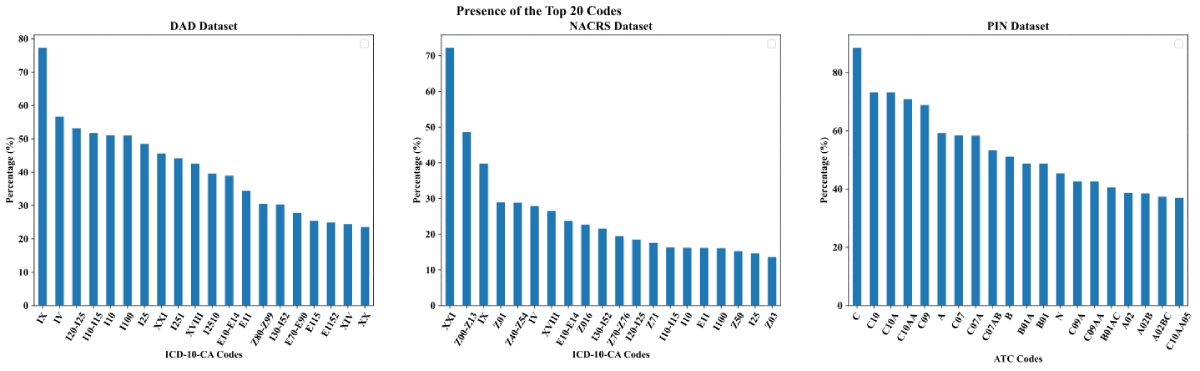

Supplement: Multimedia Appendix 1 [file medinform-v12-e52896-s001.png]
