# Supplementary material for: Unsupervised Feature Selection to Identify Important ICD-10 and ATC Codes for Machine Learning on a Cohort of Patients With Coronary Heart Disease: Retrospective Study
Source: JMIR Med Inform. 2024 Jul 26;12:e52896. doi: 10.2196/52896 (PMC11295113; doi:10.2196/52896)
Supplement: Multimedia Appendix 2 [file medinform-v12-e52896-s002.docx]

## Multimedia Appendix 1

Table S1. P-values of the difference between each model and the baseline model that outputs the mode of each class (supplement to table 3 in the manuscript)

|  | DAD | | NACRS | | PIN | |
| --- | --- | --- | --- | --- | --- | --- |
| Feature Selection Method | **Accuracy** | **BCE** | **Accuracy** | **BCE** | **Accuracy** | **BCE** |
| CAEWW | P<.001 | P<.001 | P<.001 | P<.001 | P<.001 | P<.001 |
| CAENW | P<.001 | P<.001 | P<.001 | P<.001 | P<.001 | P<.001 |
| AEFS | P=.744 | P=.650 | P=.564 | P=.430 | P=.286 | P=.154 |
| MCFS | P<.001 | P<.001 | P<.001 | P<.001 | P<.001 | P<.001 |
| PFA | P=.950 | P=.930 | P=.821 | P=.751 | P=.893 | P=.843 |
| LS | P<.001 | P<.001 | P<.001 | P<.001 | P<.001 | P<.001 |

Table S2. P-values of the difference between CAEWW and other methods using McNemar's test for the overall performance and DeLong's test for AUC-ROC (supplement to table 4 in the manuscript)

|  | DAD |  | NACRS |  | PIN |  |
| --- | --- | --- | --- | --- | --- | --- |
| Feature Selection Method | McNemar’s Test | DeLong’s test for AUC-ROC | McNemar’s Test | DeLong’s test for AUC-ROC | McNemar’s Test | DeLong’s test for AUC-ROC |
| CAENW | P<.001 | P=.019 | P=.002 | P=.417 | P<.001 | P=.665 |
| AEFS | P<.001 | P<.001 | P<.001 | P<.001 | P<.001 | P<.001 |
| MCFS | P=.145 | P<.001 | P<.001 | P=.396 | P<.001 | P<.001 |
| PFA | P<.001 | P<.001 | P<.001 | P<.001 | P<.001 | P<.001 |
| LS | P<.001 | P<.001 | P<.001 | P=.001 | P<.001 | P=.297 |

Table S3. P-values of the difference between CAEWW and other methods for the mean depth values of the selected codes by each method in the ICD-10-CA or ATC tree

|  | DAD | NACRS | PIN |
| --- | --- | --- | --- |
| Feature Selection Method |  |  |  |
| CAENW | P=0.444 | P=0.809 | P=0.787 |
| AEFS | P<.001 | P<.001 | P<.001 |
| MCFS | P<.001 | P<.001 | P=0.098 |
| PFA | P<.001 | P<.001 | P<.001 |
| LS | P<.001 | P<.001 | P<.001 |

Table S4. P-values of the difference between CAEWW and other methods for mean absolute Shapely values of features in each mortality prediction model

|  | DAD | NACRS | PIN |
| --- | --- | --- | --- |
| Feature Selection Method |  |  |  |
| CAENW | P=0.768 | P=0.895 | P=0.947 |
| AEFS | P<.001 | P<.001 | P<.001 |
| MCFS | P=0.198 | P=0.402 | P=0.237 |
| PFA | P<.001 | P<.001 | P<.001 |
| LS | P=0.907 | P=0.433 | P=0.367 |
