# Supplementary material for: Unsupervised Feature Selection to Identify Important ICD-10 and ATC Codes for Machine Learning on a Cohort of Patients With Coronary Heart Disease: Retrospective Study
Source: JMIR Med Inform. 2024 Jul 26;12:e52896. doi: 10.2196/52896 (PMC11295113; doi:10.2196/52896)
Supplement: Multimedia Appendix 3 [file medinform-v12-e52896-s003.docx]

## Multimedia Appendix 2


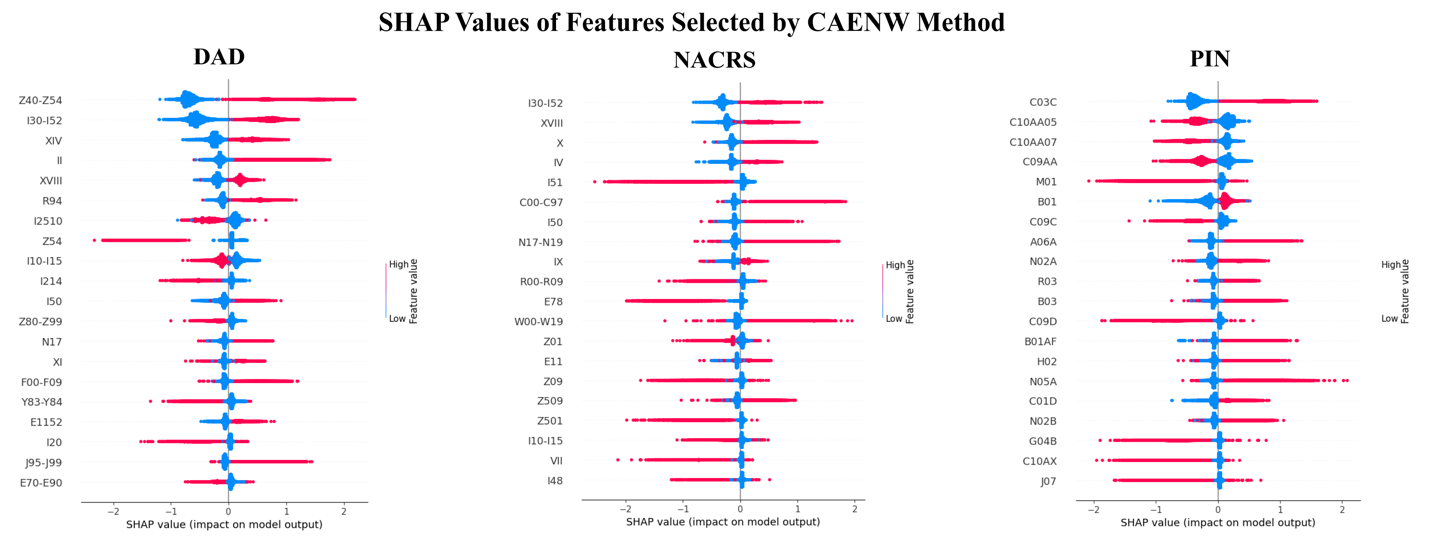


Figure S1. SHAP values of the features selected by CAENW across different datasets (20 most important features)


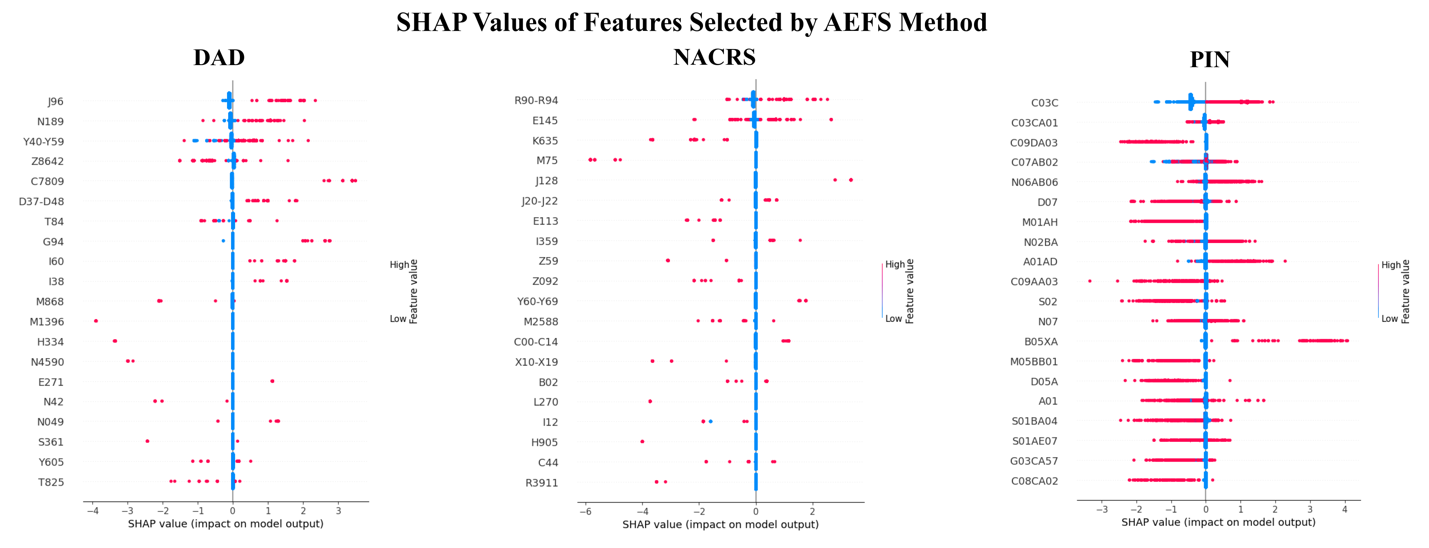


Figure S2. SHAP values of the features selected by AEFS across different datasets (20 most important features)


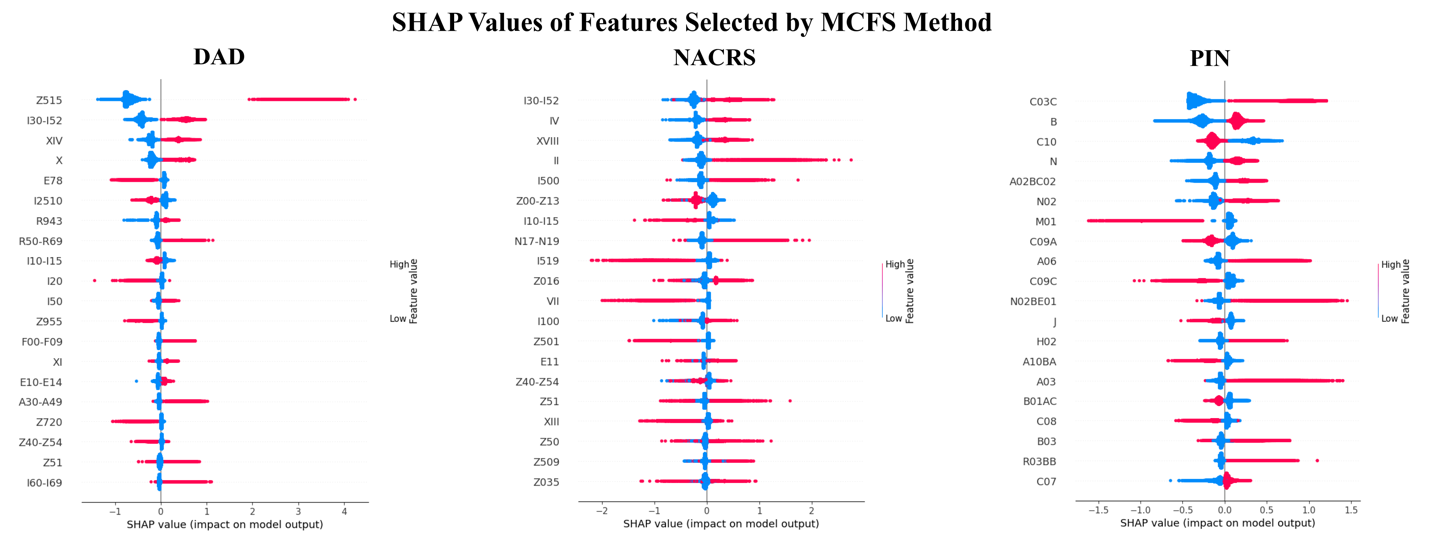


Figure S3. SHAP values of the features selected by MCFS across different datasets (20 most important features)


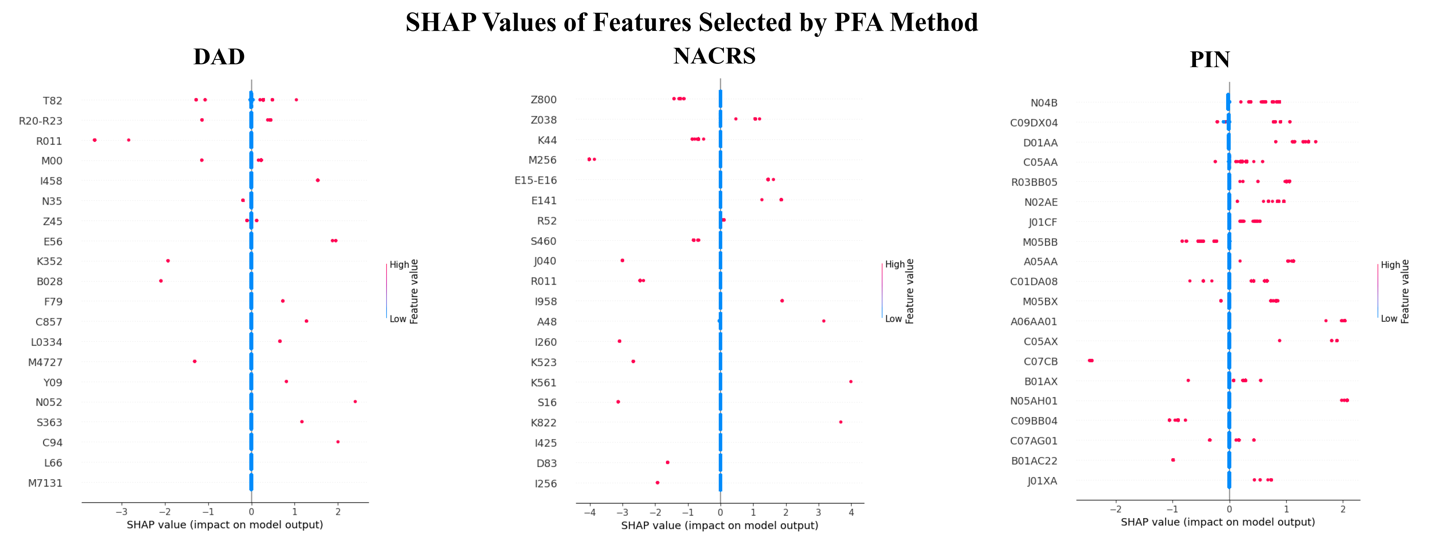


Figure S4. SHAP values of the features selected by PFA across different datasets (20 most important features)


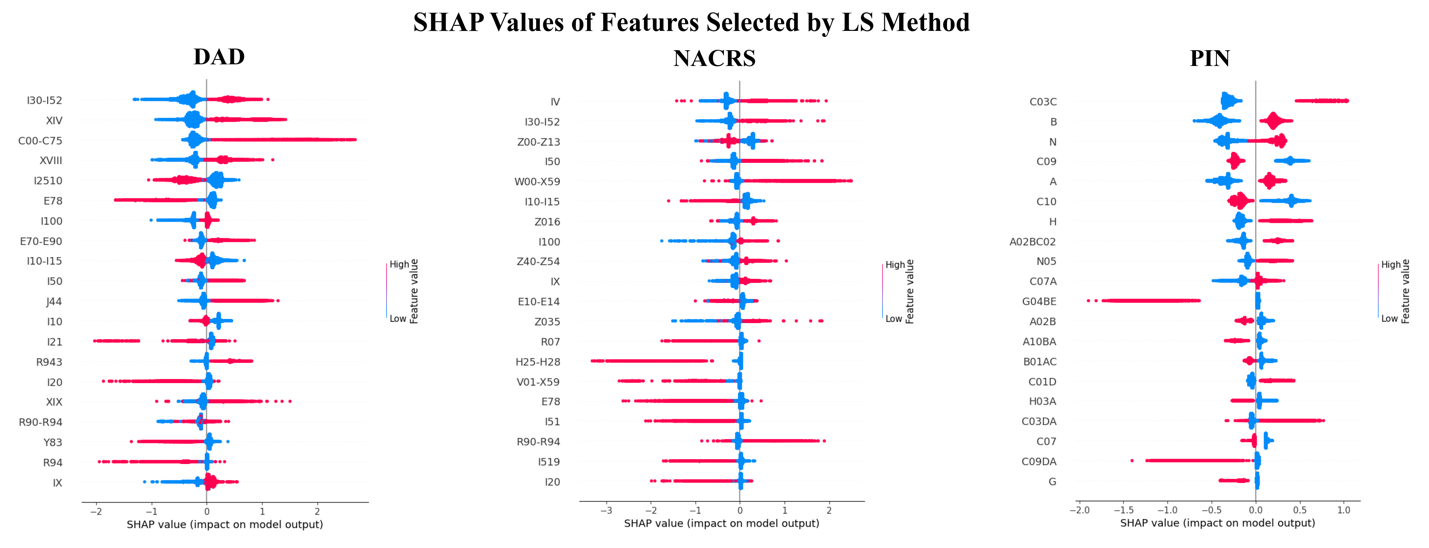


Figure S5. SHAP values of the features selected by LS across different datasets (20 most important features)
