# Supplementary material for: Unsupervised Feature Selection to Identify Important ICD-10 and ATC Codes for Machine Learning on a Cohort of Patients With Coronary Heart Disease: Retrospective Study
Source: JMIR Med Inform. 2024 Jul 26;12:e52896. doi: 10.2196/52896 (PMC11295113; doi:10.2196/52896)
Supplement: Multimedia Appendix 4 [file medinform-v12-e52896-s004.docx]

## Multimedia Appendix 3

**Table S1. Features Selected by CAEWW in the DAD Dataset**

|  | **Code** | **Description** | **Chapter** | **Mean Absolute SHAP Score** |
| --- | --- | --- | --- | --- |
| 0 | Z40-Z54 | Persons encountering health services for specific procedures and health care | XXI | 0.73 |
| 1 | I50 | Heart failure | IX | 0.37 |
| 2 | X | Diseases of the respiratory system | X | 0.21 |
| 3 | II | Neoplasms | II | 0.19 |
| 4 | Z54 | Convalescence | XXI | 0.19 |
| 5 | XVIII | Symptoms, signs and abnormal clinical and laboratory findings, not elsewhere classified | XVIII | 0.19 |
| 6 | N17 | Acute renal failure | XIV | 0.17 |
| 7 | I21 | Acute myocardial infarction | IX | 0.16 |
| 8 | I2510 | Atherosclerotic heart disease of native coronary artery | IX | 0.13 |
| 9 | I10-I15 | Hypertensive diseases | IX | 0.13 |
| 10 | R50-R69 | General symptoms and signs | XVIII | 0.13 |
| 11 | N00-N08 | Glomerular diseases | XIV | 0.11 |
| 12 | XI | Diseases of the digestive system | XI | 0.11 |
| 13 | E1152 | Type 2 diabetes mellitus with certain circulatory complications | IV | 0.1 |
| 14 | E70-E90 | Metabolic disorders | IV | 0.09 |
| 15 | I20 | Angina pectoris | IX | 0.09 |
| 16 | Z80-Z99 | Persons with potential health hazards related to family and personal history and certain conditions influencing health status | XXI | 0.09 |
| 17 | V01-X59 | Accidents | XX | 0.09 |
| 18 | Y83 | Surgical operation and other surgical procedures as the cause of abnormal reaction of the patient, or of later complication, without mention of misadventure at the time of the procedure | XX | 0.09 |
| 19 | M00-M25 | Arthropathies | XIII | 0.08 |
| 20 | V | Mental and behavioural disorders | V | 0.08 |
| 21 | E87 | Other disorders of fluid, electrolyte and acid-base balance | IV | 0.07 |
| 22 | A30-A49 | Other bacterial diseases | I | 0.07 |
| 23 | C76-C80 | Malignant neoplasms of ill-defined, secondary and unspecified sites | II | 0.07 |
| 24 | I60-I69 | Cerebrovascular diseases | IX | 0.07 |
| 25 | J95-J99 | Other diseases of the respiratory system | X | 0.06 |
| 26 | N18 | Chronic kidney disease | XIV | 0.06 |
| 27 | I25 | Chronic ischaemic heart disease | IX | 0.06 |
| 28 | I489 | Atrial fibrillation and atrial flutter, unspecified | IX | 0.06 |
| 29 | G40-G47 | Episodic and paroxysmal disorders | VI | 0.05 |
| 30 | VI | Diseases of the nervous system | VI | 0.05 |
| 31 | N30-N39 | Other diseases of urinary system | XIV | 0.05 |
| 32 | E65-E68 | Obesity and other hyperalimentation | IV | 0.05 |
| 33 | Z70-Z76 | Persons encountering health services in other circumstances | XXI | 0.05 |
| 34 | I95-I99 | Other and unspecified disorders of the circulatory system | IX | 0.05 |
| 35 | B95-B98 | Bacterial, viral and other infectious agents | I | 0.04 |
| 36 | III | Diseases of the blood and blood-forming organs and certain disorders involving the immune mechanism | III | 0.04 |
| 37 | R00-R09 | Symptoms and signs involving the circulatory and respiratory systems | XVIII | 0.04 |
| 38 | I35 | Nonrheumatic aortic valve disorders | IX | 0.04 |
| 39 | I70-I79 | Diseases of arteries, arterioles and capillaries | IX | 0.04 |
| 40 | I | Certain infectious and parasitic diseases | I | 0.04 |
| 41 | M40-M54 | Dorsopathies | XIII | 0.03 |
| 42 | E11 | Type 2 diabetes mellitus | IV | 0.03 |
| 43 | XIX | Injury, poisoning and certain other consequences of external causes | XIX | 0.03 |
| 44 | M17 | Gonarthrosis [arthrosis of knee] | XIII | 0.03 |
| 45 | Z82 | Family history of certain disabilities and chronic diseases leading to disablement | XXI | 0.03 |
| 46 | N40-N51 | Diseases of male genital organs | XIV | 0.03 |
| 47 | R9430 | Electrocardiogram suggestive of st segment elevation myocardial infarction [STEMI] | XVIII | 0.03 |
| 48 | Z86 | Personal history of certain other diseases | XXI | 0.03 |
| 49 | Y83-Y84 | Surgical and other medical procedures as the cause of abnormal reaction of the patient, or of later complication, without mention of misadventure at the time of the procedure | XX | 0.03 |
| 50 | XXII | Codes for special purposes | XXII | 0.03 |
| 51 | K80-K87 | Disorders of gallbladder, biliary tract and pancreas | XI | 0.03 |
| 52 | VII | Diseases of the eye and adnexa | VII | 0.03 |
| 53 | J09-J18 | Influenza and pneumonia | X | 0.02 |
| 54 | K20-K31 | Diseases of oesophagus, stomach and duodenum | XI | 0.02 |
| 55 | I100 | Benign hypertension | IX | 0.02 |
| 56 | I47 | Paroxysmal tachycardia | IX | 0.02 |
| 57 | Z95 | Presence of cardiac and vascular implants and grafts | XXI | 0.02 |
| 58 | N20-N23 | Urolithiasis | XIV | 0.02 |
| 59 | E10 | Type 1 diabetes mellitus | IV | 0.02 |
| 60 | I26-I28 | Pulmonary heart disease and diseases of pulmonary circulation | IX | 0.02 |
| 61 | F10-F19 | Mental and behavioural disorders due to psychoactive substance use | V | 0.02 |
| 62 | M60-M79 | Soft tissue disorders | XIII | 0.02 |
| 63 | I49 | Other cardiac arrhythmias | IX | 0.02 |
| 64 | D10-D36 | Benign neoplasms | II | 0.02 |
| 65 | E117 | Type 2 diabetes mellitus with multiple complications | IV | 0.02 |
| 66 | E00-E07 | Disorders of thyroid gland | IV | 0.02 |
| 67 | R70-R79 | Abnormal findings on examination of blood, without diagnosis | XVIII | 0.02 |
| 68 | Y40-Y59 | Drugs, medicaments and biological substances causing adverse effects in therapeutic use | XX | 0.02 |
| 69 | Z85 | Personal history of malignant neoplasm | XXI | 0.02 |
| 70 | S00-S09 | Injuries to the head | XIX | 0.02 |
| 71 | I480 | Paroxysmal atrial fibrillation | IX | 0.02 |
| 72 | R10-R19 | Symptoms and signs involving the digestive system and abdomen | XVIII | 0.02 |
| 73 | K40-K46 | Hernia | XI | 0.02 |
| 74 | M80-M94 | Osteopathies and chondropathies | XIII | 0.02 |
| 75 | Z55-Z65 | Persons with potential health hazards related to socioeconomic and psychosocial circumstances | XXI | 0.01 |
| 76 | XVII | Congenital malformations, deformations and chromosomal abnormalities | XVII | 0.01 |
| 77 | J44 | Other chronic obstructive pulmonary disease | X | 0.01 |
| 78 | R40-R46 | Symptoms and signs involving cognition, perception, emotional state and behaviour | XVIII | 0.01 |
| 79 | E14 | Unspecified diabetes mellitus | IV | 0.01 |
| 80 | N80-N98 | Noninflammatory disorders of female genital tract | XIV | 0.01 |
| 81 | I42 | Cardiomyopathy | IX | 0.01 |
| 82 | XII | Diseases of the skin and subcutaneous tissue | XII | 0.01 |
| 83 | D50-D53 | Nutritional anaemias | III | 0.01 |
| 84 | K55-K64 | Other diseases of intestines | XI | 0.01 |
| 85 | Z20-Z29 | Persons with potential health hazards related to communicable diseases | XXI | 0.01 |
| 86 | V01-V99 | Transport accidents | XX | 0.01 |
| 87 | R30-R39 | Symptoms and signs involving the urinary system | XVIII | 0.01 |
| 88 | N10-N16 | Renal tubulo-interstitial diseases | XIV | 0.01 |
| 89 | I44 | Atrioventricular and left bundle-branch block | IX | 0.01 |
| 90 | Y60-Y69 | Misadventures to patients during surgical and medical care | XX | 0.01 |
| 91 | VIII | Diseases of the ear and mastoid process | VIII | 0.01 |
| 92 | N99 | Postprocedural disorders of genitourinary system, not elsewhere classified | XIV | 0.01 |
| 93 | R25-R29 | Symptoms and signs involving the nervous and musculoskeletal systems | XVIII | 0.01 |
| 94 | G99 | Other disorders of nervous system in diseases classified elsewhere | VI | 0.01 |
| 95 | I51 | Complications and ill-defined descriptions of heart disease | IX | 0.01 |
| 96 | T36-T50 | Poisoning by drugs, medicaments and biological substances | XIX | 0.0 |
| 97 | I24 | Other acute ischaemic heart diseases | IX | 0.0 |
| 98 | C81-C96 | Malignant neoplasms, stated or presumed to be primary, of lymphoid, haematopoietic and related tissue | II | 0.0 |
| 99 | E20-E35 | Disorders of other endocrine glands | IV | 0.0 |
|  |  |  |  |  |

**Table S2. Features Selected by CAENW in the DAD Dataset**

|  | **Code** | **Description** | **Chapter** | **Mean Absolute SHAP Score** |
| --- | --- | --- | --- | --- |
| 0 | Z40-Z54 | Persons encountering health services for specific procedures and health care | XXI | 0.76 |
| 1 | I30-I52 | Other forms of heart disease | IX | 0.59 |
| 2 | XIV | Diseases of the genitourinary system | XIV | 0.28 |
| 3 | II | Neoplasms | II | 0.21 |
| 4 | XVIII | Symptoms, signs and abnormal clinical and laboratory findings, not elsewhere classified | XVIII | 0.2 |
| 5 | R94 | Abnormal results of function studies | XVIII | 0.2 |
| 6 | I2510 | Atherosclerotic heart disease of native coronary artery | IX | 0.2 |
| 7 | Z54 | Convalescence | XXI | 0.19 |
| 8 | I10-I15 | Hypertensive diseases | IX | 0.14 |
| 9 | I214 | Acute subendocardial myocardial infarction | IX | 0.13 |
| 10 | I50 | Heart failure | IX | 0.12 |
| 11 | Z80-Z99 | Persons with potential health hazards related to family and personal history and certain conditions influencing health status | XXI | 0.1 |
| 12 | N17 | Acute renal failure | XIV | 0.1 |
| 13 | XI | Diseases of the digestive system | XI | 0.1 |
| 14 | F00-F09 | Organic, including symptomatic, mental disorders | V | 0.1 |
| 15 | Y83-Y84 | Surgical and other medical procedures as the cause of abnormal reaction of the patient, or of later complication, without mention of misadventure at the time of the procedure | XX | 0.09 |
| 16 | E1152 | Type 2 diabetes mellitus with certain circulatory complications | IV | 0.09 |
| 17 | I20 | Angina pectoris | IX | 0.09 |
| 18 | J95-J99 | Other diseases of the respiratory system | X | 0.08 |
| 19 | E70-E90 | Metabolic disorders | IV | 0.08 |
| 20 | V01-X59 | Accidents | XX | 0.08 |
| 21 | J40-J47 | Chronic lower respiratory diseases | X | 0.08 |
| 22 | A30-A49 | Other bacterial diseases | I | 0.08 |
| 23 | J09-J18 | Influenza and pneumonia | X | 0.08 |
| 24 | I48 | Atrial fibrillation and flutter | IX | 0.07 |
| 25 | I25 | Chronic ischaemic heart disease | IX | 0.07 |
| 26 | M00-M25 | Arthropathies | XIII | 0.07 |
| 27 | I60-I69 | Cerebrovascular diseases | IX | 0.07 |
| 28 | E87 | Other disorders of fluid, electrolyte and acid-base balance | IV | 0.07 |
| 29 | E65-E68 | Obesity and other hyperalimentation | IV | 0.05 |
| 30 | VI | Diseases of the nervous system | VI | 0.05 |
| 31 | R07 | Pain in throat and chest | XVIII | 0.05 |
| 32 | III | Diseases of the blood and blood-forming organs and certain disorders involving the immune mechanism | III | 0.05 |
| 33 | G40-G47 | Episodic and paroxysmal disorders | VI | 0.05 |
| 34 | I95-I99 | Other and unspecified disorders of the circulatory system | IX | 0.04 |
| 35 | Z70-Z76 | Persons encountering health services in other circumstances | XXI | 0.04 |
| 36 | N40-N51 | Diseases of male genital organs | XIV | 0.04 |
| 37 | M17 | Gonarthrosis [arthrosis of knee] | XIII | 0.04 |
| 38 | E11 | Type 2 diabetes mellitus | IV | 0.04 |
| 39 | J60-J70 | Lung diseases due to external agents | X | 0.04 |
| 40 | E1123 | Type 2 diabetes mellitus with established or advanced kidney disease | IV | 0.04 |
| 41 | R00-R09 | Symptoms and signs involving the circulatory and respiratory systems | XVIII | 0.04 |
| 42 | I100 | Benign hypertension | IX | 0.03 |
| 43 | M40-M54 | Dorsopathies | XIII | 0.03 |
| 44 | I51 | Complications and ill-defined descriptions of heart disease | IX | 0.03 |
| 45 | I70-I79 | Diseases of arteries, arterioles and capillaries | IX | 0.03 |
| 46 | C30-C39 | Malignant neoplasms of respiratory and intrathoracic organs | II | 0.03 |
| 47 | J45 | Asthma | X | 0.03 |
| 48 | K80-K87 | Disorders of gallbladder, biliary tract and pancreas | XI | 0.03 |
| 49 | E10 | Type 1 diabetes mellitus | IV | 0.03 |
| 50 | VII | Diseases of the eye and adnexa | VII | 0.03 |
| 51 | F40-F48 | Neurotic, stress-related and somatoform disorders | V | 0.03 |
| 52 | Z85 | Personal history of malignant neoplasm | XXI | 0.03 |
| 53 | Z82 | Family history of certain disabilities and chronic diseases leading to disablement | XXI | 0.03 |
| 54 | R70-R79 | Abnormal findings on examination of blood, without diagnosis | XVIII | 0.03 |
| 55 | I252 | Old myocardial infarction | IX | 0.03 |
| 56 | XXII | Codes for special purposes | XXII | 0.02 |
| 57 | Y40-Y59 | Drugs, medicaments and biological substances causing adverse effects in therapeutic use | XX | 0.02 |
| 58 | K20-K31 | Diseases of oesophagus, stomach and duodenum | XI | 0.02 |
| 59 | T80-T88 | Complications of surgical and medical care, not elsewhere classified | XIX | 0.02 |
| 60 | F30-F39 | Mood [affective] disorders | V | 0.02 |
| 61 | I26-I28 | Pulmonary heart disease and diseases of pulmonary circulation | IX | 0.02 |
| 62 | E00-E07 | Disorders of thyroid gland | IV | 0.02 |
| 63 | I | Certain infectious and parasitic diseases | I | 0.02 |
| 64 | M60-M79 | Soft tissue disorders | XIII | 0.02 |
| 65 | N00-N08 | Glomerular diseases | XIV | 0.02 |
| 66 | Z86 | Personal history of certain other diseases | XXI | 0.02 |
| 67 | E117 | Type 2 diabetes mellitus with multiple complications | IV | 0.02 |
| 68 | K40-K46 | Hernia | XI | 0.02 |
| 69 | R30-R39 | Symptoms and signs involving the urinary system | XVIII | 0.02 |
| 70 | E14 | Unspecified diabetes mellitus | IV | 0.02 |
| 71 | E116 | Type 2 diabetes mellitus with other specified complications | IV | 0.02 |
| 72 | E1128 | Type 2 diabetes mellitus with other specified kidney complication not elsewhere classified | IV | 0.02 |
| 73 | Z55-Z65 | Persons with potential health hazards related to socioeconomic and psychosocial circumstances | XXI | 0.02 |
| 74 | F10-F19 | Mental and behavioural disorders due to psychoactive substance use | V | 0.01 |
| 75 | R10-R19 | Symptoms and signs involving the digestive system and abdomen | XVIII | 0.01 |
| 76 | I35 | Nonrheumatic aortic valve disorders | IX | 0.01 |
| 77 | T81 | Complications of procedures, not elsewhere classified | XIX | 0.01 |
| 78 | G47 | Sleep disorders | VI | 0.01 |
| 79 | N30-N39 | Other diseases of urinary system | XIV | 0.01 |
| 80 | Z20-Z29 | Persons with potential health hazards related to communicable diseases | XXI | 0.01 |
| 81 | XII | Diseases of the skin and subcutaneous tissue | XII | 0.01 |
| 82 | I208 | Other forms of angina pectoris | IX | 0.01 |
| 83 | M05-M14 | Inflammatory polyarthropathies | XIII | 0.01 |
| 84 | S30-S39 | Injuries to the abdomen, lower back, lumbar spine and pelvis | XIX | 0.01 |
| 85 | L03 | Cellulitis | XII | 0.01 |
| 86 | K55-K64 | Other diseases of intestines | XI | 0.01 |
| 87 | D50-D53 | Nutritional anaemias | III | 0.01 |
| 88 | R25-R29 | Symptoms and signs involving the nervous and musculoskeletal systems | XVIII | 0.01 |
| 89 | Z91 | Personal history of risk-factors, not elsewhere classified | XXI | 0.01 |
| 90 | M80-M94 | Osteopathies and chondropathies | XIII | 0.01 |
| 91 | I79 | Disorders of arteries, arterioles and capillaries in diseases classified elsewhere | IX | 0.01 |
| 92 | I24 | Other acute ischaemic heart diseases | IX | 0.01 |
| 93 | I47 | Paroxysmal tachycardia | IX | 0.01 |
| 94 | V01-V99 | Transport accidents | XX | 0.01 |
| 95 | S20-S29 | Injuries to the thorax | XIX | 0.0 |
| 96 | VIII | Diseases of the ear and mastoid process | VIII | 0.0 |
| 97 | C81-C96 | Malignant neoplasms, stated or presumed to be primary, of lymphoid, haematopoietic and related tissue | II | 0.0 |
| 98 | M20-M25 | Other joint disorders | XIII | 0.0 |
| 99 | X60-X84 | Intentional self-harm | XX | 0.0 |
|  |  |  |  |  |

**Table S3. Features Selected by AEFS in the DAD Dataset**

|  | **Code** | **Description** | **Chapter** | **Mean Absolute SHAP Score** |
| --- | --- | --- | --- | --- |
| 0 | J96 | Respiratory failure, not elsewhere classified | X | 0.14 |
| 1 | N189 | Chronic kidney disease, unspecified | XIV | 0.1 |
| 2 | Y40-Y59 | Drugs, medicaments and biological substances causing adverse effects in therapeutic use | XX | 0.06 |
| 3 | Z8642 | Personal history of tobacco abuse | XXI | 0.05 |
| 4 | C7809 | Secondary malignant neoplasm of lung, unspecified side | II | 0.03 |
| 5 | D37-D48 | Neoplasms of uncertain or unknown behaviour | II | 0.02 |
| 6 | T84 | Complications of internal orthopaedic prosthetic devices, implants and grafts | XIX | 0.01 |
| 7 | G94 | Other disorders of brain in diseases classified elsewhere | VI | 0.0 |
| 8 | I60 | Subarachnoid haemorrhage | IX | 0.0 |
| 9 | I38 | Endocarditis, valve unspecified | IX | 0.0 |
| 10 | M868 | Other osteomyelitis | XIII | 0.0 |
| 11 | M1396 | Arthritis, unspecified, lower leg | XIII | 0.0 |
| 12 | H334 | Traction detachment of retina | VII | 0.0 |
| 13 | N4590 | Epididymitis | XIV | 0.0 |
| 14 | E271 | Primary adrenocortical insufficiency | IV | 0.0 |
| 15 | N42 | Other disorders of prostate | XIV | 0.0 |
| 16 | N049 | Nephrotic syndrome : unspecified | XIV | 0.0 |
| 17 | S361 | Injury of liver or gallbladder | XIX | 0.0 |
| 18 | Y605 | During heart catheterization | XX | 0.0 |
| 19 | T825 | Mechanical complication of other cardiac and vascular devices and implants | XIX | 0.0 |
| 20 | Z76802 | Kidney transplant candidate | XXI | 0.0 |
| 21 | S52 | Fracture of forearm | XIX | 0.0 |
| 22 | Z982 | Presence of cerebrospinal fluid drainage device | XXI | 0.0 |
| 23 | G82490 | Spastic quadriplegia, unspecified, at cervical spine level C1 to C4 | VI | 0.0 |
| 24 | Q63 | Other congenital malformations of kidney | XVII | 0.0 |
| 25 | V234 | Motorcycle rider injured in collision with car, pick-up truck or van : driver injured in traffic accident | XX | 0.0 |
| 26 | S09 | Other and unspecified injuries of head | XIX | 0.0 |
| 27 | Y531 | Other antacids and anti-gastric-secretion drugs | XX | 0.0 |
| 28 | K598 | Other specified functional intestinal disorders | XI | 0.0 |
| 29 | U988 | Other specified place of occurrence | U988 | 0.0 |
| 30 | I2512 | Atherosclerotic heart disease of nonautologous biological bypass graft | IX | 0.0 |
| 31 | N008 | Acute nephritic syndrome : other | XIV | 0.0 |
| 32 | K45 | Other abdominal hernia | XI | 0.0 |
| 33 | L010 | Impetigo [any organism] [any site] | XII | 0.0 |
| 34 | S0626 | Diffuse brain injury with open intracranial wound | XIX | 0.0 |
| 35 | B348 | Other viral infections of unspecified site | I | 0.0 |
| 36 | S62610 | Fracture of distal phalanx of finger, closed | XIX | 0.0 |
| 37 | N701 | Chronic salpingitis and oophoritis | XIV | 0.0 |
| 38 | S005 | Superficial injury of lip and oral cavity | XIX | 0.0 |
| 39 | R293 | Abnormal posture | XVIII | 0.0 |
| 40 | M402 | Other and unspecified kyphosis | XIII | 0.0 |
| 41 | L020 | Cutaneous abscess, furuncle and carbuncle of face | XII | 0.0 |
| 42 | C864 | Blastic NK-cell lymphoma | II | 0.0 |
| 43 | T4040 | Poisoning by fentanyl and derivatives | XIX | 0.0 |
| 44 | G418 | Other status epilepticus | VI | 0.0 |
| 45 | D731 | Hypersplenism | III | 0.0 |
| 46 | M873 | Other secondary osteonecrosis | XIII | 0.0 |
| 47 | J340 | Abscess, furuncle and carbuncle of nose | X | 0.0 |
| 48 | N251 | Nephrogenic diabetes insipidus | XIV | 0.0 |
| 49 | Y750 | Neurological devices associated with adverse incidents : diagnostic and monitoring devices | XX | 0.0 |
| 50 | Y08 | Assault by other specified means | XX | 0.0 |
| 51 | S42800 | Fracture of other parts of shoulder and upper arm, closed | XIX | 0.0 |
| 52 | S4408 | Other and unspecified injury of ulnar nerve at upper arm level | XIX | 0.0 |
| 53 | V385 | Occupant of three-wheeled motor vehicle injured in noncollision transport accident : driver injured in traffic accident | XX | 0.0 |
| 54 | T912 | Sequelae of other fracture of thorax and pelvis | XIX | 0.0 |
| 55 | X95 | Assault by other and unspecified firearm discharge | XX | 0.0 |
| 56 | S52020 | Monteggia's fracture, closed | XIX | 0.0 |
| 57 | Z9221 | Personal history of long-term (current) use of postmenopausal hormone replacement therapy | XXI | 0.0 |
| 58 | S527 | Multiple fractures of forearm | XIX | 0.0 |
| 59 | S62111 | Fracture of triquetral bone, open | XIX | 0.0 |
| 60 | T524 | Toxic effect: Ketones | XIX | 0.0 |
| 61 | S636 | Sprain and strain of finger(s) | XIX | 0.0 |
| 62 | S767 | Injury of multiple muscles and tendons at hip and thigh level | XIX | 0.0 |
| 63 | T335 | Superficial frostbite of wrist and hand | XIX | 0.0 |
| 64 | Z352 | Supervision of pregnancy with other poor reproductive or obstetric history | XXI | 0.0 |
| 65 | S36911 | Laceration of unspecified intra-abdominal organ, with open wound into cavity | XIX | 0.0 |
| 66 | 80723 | Squamous cell carcinoma, large cell, nonkeratinizing | NEOPLASM | 0.0 |
| 67 | S36020 | Subcapsular haematoma of spleen involving >50% of surface, or intraparenchymal haematoma > 5cm, capsular laceration > 3cm or involving trabecular vessel (grade III) without open wound into cavity | XIX | 0.0 |
| 68 | S26890 | Unspecified injury of heart without open wound into thoracic cavity | XIX | 0.0 |
| 69 | D035 | Melanoma in situ of trunk | II | 0.0 |
| 70 | D129 | Benign neoplasm: Anus and anal canal | II | 0.0 |
| 71 | D407 | Neoplasm of uncertain or unknown behaviour: Other male genital organs | II | 0.0 |
| 72 | E348 | Other specified endocrine disorders | IV | 0.0 |
| 73 | F408 | Other phobic anxiety disorders | V | 0.0 |
| 74 | G631 | Polyneuropathy in neoplastic disease | VI | 0.0 |
| 75 | H264 | After-cataract | VII | 0.0 |
| 76 | I378 | Other pulmonary valve disorders | IX | 0.0 |
| 77 | J206 | Acute bronchitis due to rhinovirus | X | 0.0 |
| 78 | J35 | Chronic diseases of tonsils and adenoids | X | 0.0 |
| 79 | K229 | Disease of oesophagus, unspecified | XI | 0.0 |
| 80 | L059 | Pilonidal cyst without abscess | XII | 0.0 |
| 81 | L729 | Follicular cyst of skin and subcutaneous tissue, unspecified | XII | 0.0 |
| 82 | M052 | Rheumatoid vasculitis | XIII | 0.0 |
| 83 | M2461 | Ankylosis of joint, shoulder region | XIII | 0.0 |
| 84 | M4645 | Discitis, unspecified, thoracolumbar region | XIII | 0.0 |
| 85 | M763 | Iliotibial band syndrome | XIII | 0.0 |
| 86 | 85623 | Epithelial-myoepithelial carcinoma | NEOPLASM | 0.0 |
| 87 | O008 | Other ectopic pregnancy | XV | 0.0 |
| 88 | O99201 | Endocrine, nutritional and metabolic diseases complicating pregnancy, childbirth and the puerperium, delivered, with or without mention of antepartum condition | XV | 0.0 |
| 89 | Q06 | Other congenital malformations of spinal cord | XVII | 0.0 |
| 90 | Q182 | Other branchial cleft malformations | XVII | 0.0 |
| 91 | Q249 | Congenital malformation of heart, unspecified | XVII | 0.0 |
| 92 | Q796 | Ehlers-Danlos syndrome | XVII | 0.0 |
| 93 | R700 | Elevated erythrocyte sedimentation rate | XVIII | 0.0 |
| 94 | R770 | Abnormality of albumin | XVIII | 0.0 |
| 95 | S101 | Other and unspecified superficial injuries of throat | XIX | 0.0 |
| 96 | N511 | Disorders of testis and epididymis in diseases classified elsewhere | XIV | 0.0 |
|  |  |  |  |  |

**Table S4. Features Selected by MCFS in the DAD Dataset**

|  | **Code** | **Description** | **Chapter** | **Mean Absolute SHAP Score** |
| --- | --- | --- | --- | --- |
| 0 | Z515 | Palliative care | XXI | 0.77 |
| 1 | I30-I52 | Other forms of heart disease | IX | 0.46 |
| 2 | XIV | Diseases of the genitourinary system | XIV | 0.26 |
| 3 | X | Diseases of the respiratory system | X | 0.24 |
| 4 | E78 | Disorders of lipoprotein metabolism and other lipidaemias | IV | 0.17 |
| 5 | I2510 | Atherosclerotic heart disease of native coronary artery | IX | 0.13 |
| 6 | R943 | Abnormal results of cardiovascular function studies | XVIII | 0.1 |
| 7 | R50-R69 | General symptoms and signs | XVIII | 0.1 |
| 8 | I10-I15 | Hypertensive diseases | IX | 0.08 |
| 9 | I20 | Angina pectoris | IX | 0.07 |
| 10 | I50 | Heart failure | IX | 0.07 |
| 11 | Z955 | Presence of coronary angioplasty implant and graft | XXI | 0.06 |
| 12 | F00-F09 | Organic, including symptomatic, mental disorders | V | 0.06 |
| 13 | XI | Diseases of the digestive system | XI | 0.05 |
| 14 | E10-E14 | Diabetes mellitus | IV | 0.05 |
| 15 | A30-A49 | Other bacterial diseases | I | 0.05 |
| 16 | Z720 | Tobacco use | XXI | 0.05 |
| 17 | Z40-Z54 | Persons encountering health services for specific procedures and health care | XXI | 0.05 |
| 18 | Z51 | Other medical care | XXI | 0.05 |
| 19 | I60-I69 | Cerebrovascular diseases | IX | 0.04 |
| 20 | E70-E90 | Metabolic disorders | IV | 0.04 |
| 21 | I500 | Congestive heart failure | IX | 0.04 |
| 22 | I95-I99 | Other and unspecified disorders of the circulatory system | IX | 0.04 |
| 23 | E65-E68 | Obesity and other hyperalimentation | IV | 0.04 |
| 24 | I21 | Acute myocardial infarction | IX | 0.04 |
| 25 | I48 | Atrial fibrillation and flutter | IX | 0.04 |
| 26 | E115 | Type 2 diabetes mellitus with peripheral circulatory complications | IV | 0.03 |
| 27 | Z54 | Convalescence | XXI | 0.03 |
| 28 | E87 | Other disorders of fluid, electrolyte and acid-base balance | IV | 0.03 |
| 29 | E119 | Type 2 diabetes mellitus without complications | IV | 0.03 |
| 30 | R00-R09 | Symptoms and signs involving the circulatory and respiratory systems | XVIII | 0.03 |
| 31 | Z85 | Personal history of malignant neoplasm | XXI | 0.03 |
| 32 | XVIII | Symptoms, signs and abnormal clinical and laboratory findings, not elsewhere classified | XVIII | 0.02 |
| 33 | Y83-Y84 | Surgical and other medical procedures as the cause of abnormal reaction of the patient, or of later complication, without mention of misadventure at the time of the procedure | XX | 0.02 |
| 34 | Z8642 | Personal history of tobacco abuse | XXI | 0.02 |
| 35 | R10-R19 | Symptoms and signs involving the digestive system and abdomen | XVIII | 0.02 |
| 36 | I219 | Acute myocardial infarction, unspecified | IX | 0.02 |
| 37 | Y40-Y84 | Complications of medical and surgical care | XX | 0.02 |
| 38 | Z80-Z99 | Persons with potential health hazards related to family and personal history and certain conditions influencing health status | XXI | 0.02 |
| 39 | I51 | Complications and ill-defined descriptions of heart disease | IX | 0.02 |
| 40 | Z82 | Family history of certain disabilities and chronic diseases leading to disablement | XXI | 0.02 |
| 41 | I2519 | Atherosclerotic heart disease of unspecified type of vessel, native or graft | IX | 0.02 |
| 42 | R9430 | Electrocardiogram suggestive of st segment elevation myocardial infarction [STEMI] | XVIII | 0.02 |
| 43 | E789 | Disorder of lipoprotein metabolism, unspecified | IV | 0.02 |
| 44 | Z70-Z76 | Persons encountering health services in other circumstances | XXI | 0.02 |
| 45 | I200 | Unstable angina | IX | 0.02 |
| 46 | Y832 | Surgical operation with anastomosis, bypass or graft | XX | 0.02 |
| 47 | E1164 | Type 2 diabetes mellitus with poor control, so described | IV | 0.02 |
| 48 | XXI | Factors influencing health status and contact with health services | XXI | 0.02 |
| 49 | R73 | Elevated blood glucose level | XVIII | 0.01 |
| 50 | E10 | Type 1 diabetes mellitus | IV | 0.01 |
| 51 | N083 | Glomerular disorders in diabetes mellitus | XIV | 0.01 |
| 52 | Z50 | Care involving use of rehabilitation procedures | XXI | 0.01 |
| 53 | Z751 | Person awaiting admission to adequate facility elsewhere | XXI | 0.01 |
| 54 | N0835 | Glomerular disorders in diabetes mellitus, chronic kidney disease, stage 5 | XIV | 0.01 |
| 55 | E145 | Unspecified diabetes mellitus with peripheral circulatory complications | IV | 0.01 |
| 56 | R40-R46 | Symptoms and signs involving cognition, perception, emotional state and behaviour | XVIII | 0.01 |
| 57 | Z950 | Presence of electronic cardiac devices | XXI | 0.01 |
| 58 | I4890 | Atrial fibrillation, unspecified | IX | 0.01 |
| 59 | I252 | Old myocardial infarction | IX | 0.01 |
| 60 | I25 | Chronic ischaemic heart disease | IX | 0.01 |
| 61 | E00-E07 | Disorders of thyroid gland | IV | 0.01 |
| 62 | E1123 | Type 2 diabetes mellitus with established or advanced kidney disease | IV | 0.01 |
| 63 | E11 | Type 2 diabetes mellitus | IV | 0.01 |
| 64 | E112 | Type 2 diabetes mellitus with renal complications | IV | 0.01 |
| 65 | E1128 | Type 2 diabetes mellitus with other specified kidney complication not elsewhere classified | IV | 0.01 |
| 66 | I | Certain infectious and parasitic diseases | I | 0.01 |
| 67 | E14 | Unspecified diabetes mellitus | IV | 0.01 |
| 68 | IX | Diseases of the circulatory system | IX | 0.01 |
| 69 | E1178 | Type 2 diabetes mellitus with multiple other complications | IV | 0.01 |
| 70 | I251 | Atherosclerotic heart disease | IX | 0.01 |
| 71 | Y840 | Cardiac catheterization | XX | 0.01 |
| 72 | R30-R39 | Symptoms and signs involving the urinary system | XVIII | 0.01 |
| 73 | I20-I25 | Ischaemic heart diseases | IX | 0.01 |
| 74 | Z20-Z29 | Persons with potential health hazards related to communicable diseases | XXI | 0.01 |
| 75 | R9431 | Abnormal cardiovascular function studies (biomarkers or ECG) suggestive of non st segment elevation myocardial infarction [NSTEMI] | XVIII | 0.01 |
| 76 | Z75 | Problems related to medical facilities and other health care | XXI | 0.0 |
| 77 | E117 | Type 2 diabetes mellitus with multiple complications | IV | 0.0 |
| 78 | VI | Diseases of the nervous system | VI | 0.0 |
| 79 | J449 | Chronic obstructive pulmonary disease, unspecified | X | 0.0 |
| 80 | Z92 | Personal history of medical treatment | XXI | 0.0 |
| 81 | XII | Diseases of the skin and subcutaneous tissue | XII | 0.0 |
| 82 | I100 | Benign hypertension | IX | 0.0 |
| 83 | Y40-Y59 | Drugs, medicaments and biological substances causing adverse effects in therapeutic use | XX | 0.0 |
| 84 | E86 | Volume depletion | IV | 0.0 |
| 85 | I214 | Acute subendocardial myocardial infarction | IX | 0.0 |
| 86 | Z951 | Presence of aortocoronary bypass graft | XXI | 0.0 |
| 87 | E116 | Type 2 diabetes mellitus with other specified complications | IV | 0.0 |
| 88 | I2511 | Atherosclerotic heart disease of autologous vein bypass graft | IX | 0.0 |
| 89 | I24 | Other acute ischaemic heart diseases | IX | 0.0 |
| 90 | I2149 | Acute subendocardial myocardial infarction, unspecified site | IX | 0.0 |
| 91 | E102 | Type 1 diabetes mellitus with renal complications | IV | 0.0 |
| 92 | T814 | Infection following a procedure, not elsewhere classified | XIX | 0.0 |
| 93 | N0839 | Unspecified glomerular disorders in diabetes mellitus | XIV | 0.0 |
| 94 | N00-N08 | Glomerular diseases | XIV | 0.0 |
| 95 | I10 | Essential (primary) hypertension | IX | 0.0 |
| 96 | Z91 | Personal history of risk-factors, not elsewhere classified | XXI | 0.0 |
| 97 | N08 | Glomerular disorders in diseases classified elsewhere | XIV | 0.0 |
| 98 | I2514 | Atherosclerotic heart disease of unspecified type of bypass graft | IX | 0.0 |
| 99 | I518 | Other ill-defined heart diseases | IX | 0.0 |
|  |  |  |  |  |

**Table S5. Features Selected by PFA in the DAD Dataset**

|  | **Code** | **Description** | **Chapter** | **Mean Absolute SHAP Score** |
| --- | --- | --- | --- | --- |
| 0 | T82 | Complications of cardiac and vascular prosthetic devices, implants and grafts | XIX | 0.01 |
| 1 | R20-R23 | Symptoms and signs involving the skin and subcutaneous tissue | XVIII | 0.0 |
| 2 | R011 | Cardiac murmur, unspecified | XVIII | 0.0 |
| 3 | M00 | Pyogenic arthritis | XIII | 0.0 |
| 4 | I458 | Other specified conduction disorders | IX | 0.0 |
| 5 | N35 | Urethral stricture | XIV | 0.0 |
| 6 | Z45 | Adjustment and management of implanted device | XXI | 0.0 |
| 7 | E56 | Other vitamin deficiencies | IV | 0.0 |
| 8 | K352 | Acute appendicitis with generalized peritonitis | XI | 0.0 |
| 9 | B028 | Zoster with other complications | I | 0.0 |
| 10 | F79 | Unspecified mental retardation | V | 0.0 |
| 11 | C857 | Other specified types of non-Hodgkin lymphoma | II | 0.0 |
| 12 | L0334 | Cellulitis of back [any part except buttock] | XII | 0.0 |
| 13 | M4727 | Other spondylosis with radiculopathy, lumbosacral region | XIII | 0.0 |
| 14 | Y09 | Assault by unspecified means | XX | 0.0 |
| 15 | N052 | Unspecified nephritic syndrome : diffuse membranous glomerulonephritis | XIV | 0.0 |
| 16 | S363 | Injury of stomach | XIX | 0.0 |
| 17 | C94 | Other leukaemias of specified cell type | II | 0.0 |
| 18 | L66 | Cicatricial alopecia [scarring hair loss] | XII | 0.0 |
| 19 | M7131 | Other bursal cyst, shoulder region | XIII | 0.0 |
| 20 | S62601 | Fracture of middle or proximal phalanx of finger, open | XIX | 0.0 |
| 21 | S43390 | Dislocation of unspecified part of shoulder girdle, closed | XIX | 0.0 |
| 22 | K286 | Gastrojejunal ulcer : chronic or unspecified with both haemorrhage and perforation | XI | 0.0 |
| 23 | K400 | Bilateral inguinal hernia, with obstruction, without gangrene | XI | 0.0 |
| 24 | Z863 | Personal history of endocrine, nutritional and metabolic diseases | XXI | 0.0 |
| 25 | N211 | Calculus in urethra | XIV | 0.0 |
| 26 | C500 | Malignant neoplasm: Nipple and areola | II | 0.0 |
| 27 | K37 | Unspecified appendicitis | XI | 0.0 |
| 28 | G403 | Generalized idiopathic epilepsy and epileptic syndromes | VI | 0.0 |
| 29 | M8440 | Pathological fracture, not elsewhere classified, multiple sites | XIII | 0.0 |
| 30 | M8414 | Nonunion of fracture [pseudarthrosis], hand | XIII | 0.0 |
| 31 | M8581 | Other specified disorders of bone density and structure, shoulder region | XIII | 0.0 |
| 32 | N032 | Chronic nephritic syndrome : diffuse membranous glomerulonephritis | XIV | 0.0 |
| 33 | N369 | Urethral disorder, unspecified | XIV | 0.0 |
| 34 | M8410 | Nonunion of fracture [pseudarthrosis], multiple sites | XIII | 0.0 |
| 35 | M8405 | Malunion of fracture, pelvic region and thigh | XIII | 0.0 |
| 36 | S32201 | Fracture of coccyx, open | XIX | 0.0 |
| 37 | N414 | Granulomatous prostatitis | XIV | 0.0 |
| 38 | Q780 | Osteogenesis imperfecta | XVII | 0.0 |
| 39 | Z76807 | Multiple transplant candidate | XXI | 0.0 |
| 40 | Z593 | Problems related to living in residential institution | XXI | 0.0 |
| 41 | Z4180 | Procedures for transgender reassignment | XXI | 0.0 |
| 42 | Z004 | General psychiatric examination, not elsewhere classified | XXI | 0.0 |
| 43 | Y791 | Orthopaedic devices associated with adverse incidents : therapeutic (nonsurgical) and rehabilitative devices | XX | 0.0 |
| 44 | Y485 | Therapeutic gases | XX | 0.0 |
| 45 | V599 | Occupant [any] of pick-up truck or van injured in unspecified traffic accident | XX | 0.0 |
| 46 | V38 | Occupant of three-wheeled motor vehicle injured in noncollision transport accident | XX | 0.0 |
| 47 | T889 | Complication of surgical and medical care, unspecified | XIX | 0.0 |
| 48 | T8458 | Infection and inflammatory reaction due to other joint prosthesis | XIX | 0.0 |
| 49 | S968 | Injury of other muscles and tendons at ankle and foot level | XIX | 0.0 |
| 50 | S932 | Rupture of ligaments at ankle and foot level | XIX | 0.0 |
| 51 | S72120 | New code | XIX | 0.0 |
| 52 | M320 | Drug-induced systemic lupus erythematosus | XIII | 0.0 |
| 53 | S2908 | Other and unspecified injury of muscle and tendon at thorax level | XIX | 0.0 |
| 54 | S032 | Dislocation of tooth | XIX | 0.0 |
| 55 | R064 | Hyperventilation | XVIII | 0.0 |
| 56 | M413 | Thoracogenic scoliosis | XIII | 0.0 |
| 57 | 80903 | Basal cell carcinoma NOS | NEOPLASM | 0.0 |
| 58 | M2585 | Other specified joint disorders, pelvic region and thigh | XIII | 0.0 |
| 59 | E11782 | Type 2 diabetes mellitus with other multiple complications, inadequately controlled with diet or oral agents (and insulin not used to stabilize) | IV | 0.0 |
| 60 | G254 | Drug-induced chorea | VI | 0.0 |
| 61 | G112 | Late-onset cerebellar ataxia | VI | 0.0 |
| 62 | G020 | Meningitis in viral diseases classified elsewhere | VI | 0.0 |
| 63 | F508 | Other eating disorders | V | 0.0 |
| 64 | F453 | Somatoform autonomic dysfunction | V | 0.0 |
| 65 | F024 | Dementia in human immunodeficiency virus [HIV] disease | V | 0.0 |
| 66 | E539 | Vitamin B deficiency, unspecified | IV | 0.0 |
| 67 | E11789 | Type 2 diabetes mellitus with other multiple complications, level of control unspecified | IV | 0.0 |
| 68 | E11409 | Type 2 diabetes mellitus with mononeuropathy nec, level of control unspecified | IV | 0.0 |
| 69 | M2467 | Ankylosis of joint, ankle and foot | XIII | 0.0 |
| 70 | E11299 | Type 2 diabetes mellitus with renal complication unspecified, level of control unspecified | IV | 0.0 |
| 71 | E11283 | Type 2 diabetes mellitus with other specified renal complication, inadequately controlled with diet or oral agents but adequately controlled with insulin | IV | 0.0 |
| 72 | D6930 | Evans' syndrome | III | 0.0 |
| 73 | D110 | Benign neoplasm: Parotid gland | II | 0.0 |
| 74 | C835 | Lymphoblastic (diffuse) lymphoma | II | 0.0 |
| 75 | C540 | Malignant neoplasm: Isthmus uteri | II | 0.0 |
| 76 | C468 | Kaposi sarcoma of multiple organs | II | 0.0 |
| 77 | C441 | Malignant neoplasm: Skin of eyelid, including canthus | II | 0.0 |
| 78 | G578 | Other mononeuropathies of lower limb | VI | 0.0 |
| 79 | G998 | Other specified disorders of nervous system in diseases classified elsewhere | VI | 0.0 |
| 80 | H401 | Primary open-angle glaucoma | VII | 0.0 |
| 81 | H603 | Other infective otitis externa | VIII | 0.0 |
| 82 | M1383 | Other specified arthritis, forearm | XIII | 0.0 |
| 83 | M1046 | Other secondary gout, lower leg | XIII | 0.0 |
| 84 | 99601 | Chronic myeloproliferative disease | NEOPLASM | 0.0 |
| 85 | K669 | Disorder of peritoneum, unspecified | XI | 0.0 |
| 86 | K275 | Peptic ulcer, site unspecified : chronic or unspecified with perforation | XI | 0.0 |
| 87 | K229 | Disease of oesophagus, unspecified | XI | 0.0 |
| 88 | K146 | Glossodynia | XI | 0.0 |
| 89 | K111 | Hypertrophy of salivary gland | XI | 0.0 |
| 90 | K021 | Caries of dentine | XI | 0.0 |
| 91 | J341 | Cyst and mucocele of nose and nasal sinus | X | 0.0 |
| 92 | J206 | Acute bronchitis due to rhinovirus | X | 0.0 |
| 93 | J202 | Acute bronchitis due to streptococcus | X | 0.0 |
| 94 | J028 | Acute pharyngitis due to other specified organisms | X | 0.0 |
| 95 | J018 | Other acute sinusitis | X | 0.0 |
| 96 | I692 | Sequelae of other nontraumatic intracranial haemorrhage | IX | 0.0 |
| 97 | I1591 | Secondary hypertension, unspecified, malignant | IX | 0.0 |
| 98 | I1500 | Renovascular hypertension, benign or unspecified | IX | 0.0 |
| 99 | K8080 | Other cholelithiasis without mention of obstruction | XI | 0.0 |
|  |  |  |  |  |

**Table S6. Features Selected by LS in the DAD Dataset**

|  | **Code** | **Description** | **Chapter** | **Mean Absolute SHAP Score** |
| --- | --- | --- | --- | --- |
| 0 | I30-I52 | Other forms of heart disease | IX | 0.37 |
| 1 | XIV | Diseases of the genitourinary system | XIV | 0.32 |
| 2 | C00-C75 | Malignant neoplasms, stated or presumed to be primary, of specified sites, except of lymphoid, haematopoietic and related tissue | II | 0.29 |
| 3 | XVIII | Symptoms, signs and abnormal clinical and laboratory findings, not elsewhere classified | XVIII | 0.27 |
| 4 | I2510 | Atherosclerotic heart disease of native coronary artery | IX | 0.25 |
| 5 | E78 | Disorders of lipoprotein metabolism and other lipidaemias | IV | 0.23 |
| 6 | I100 | Benign hypertension | IX | 0.14 |
| 7 | E70-E90 | Metabolic disorders | IV | 0.14 |
| 8 | I10-I15 | Hypertensive diseases | IX | 0.13 |
| 9 | I50 | Heart failure | IX | 0.13 |
| 10 | J44 | Other chronic obstructive pulmonary disease | X | 0.12 |
| 11 | I10 | Essential (primary) hypertension | IX | 0.11 |
| 12 | I21 | Acute myocardial infarction | IX | 0.11 |
| 13 | R943 | Abnormal results of cardiovascular function studies | XVIII | 0.11 |
| 14 | I20 | Angina pectoris | IX | 0.11 |
| 15 | XIX | Injury, poisoning and certain other consequences of external causes | XIX | 0.11 |
| 16 | R90-R94 | Abnormal findings on diagnostic imaging and in function studies, without diagnosis | XVIII | 0.11 |
| 17 | Y83 | Surgical operation and other surgical procedures as the cause of abnormal reaction of the patient, or of later complication, without mention of misadventure at the time of the procedure | XX | 0.1 |
| 18 | R94 | Abnormal results of function studies | XVIII | 0.09 |
| 19 | IX | Diseases of the circulatory system | IX | 0.09 |
| 20 | N17-N19 | Renal failure | XIV | 0.09 |
| 21 | J18 | Pneumonia, organism unspecified | X | 0.09 |
| 22 | R07 | Pain in throat and chest | XVIII | 0.08 |
| 23 | I214 | Acute subendocardial myocardial infarction | IX | 0.08 |
| 24 | Z955 | Presence of coronary angioplasty implant and graft | XXI | 0.07 |
| 25 | N17 | Acute renal failure | XIV | 0.07 |
| 26 | J40-J47 | Chronic lower respiratory diseases | X | 0.07 |
| 27 | I251 | Atherosclerotic heart disease | IX | 0.06 |
| 28 | Z864 | Personal history of psychoactive substance abuse | XXI | 0.06 |
| 29 | Z80-Z99 | Persons with potential health hazards related to family and personal history and certain conditions influencing health status | XXI | 0.06 |
| 30 | E112 | Type 2 diabetes mellitus with renal complications | IV | 0.06 |
| 31 | Z54 | Convalescence | XXI | 0.06 |
| 32 | E115 | Type 2 diabetes mellitus with peripheral circulatory complications | IV | 0.06 |
| 33 | I500 | Congestive heart failure | IX | 0.05 |
| 34 | N179 | Acute renal failure, unspecified | XIV | 0.05 |
| 35 | Z72 | Problems related to lifestyle | XXI | 0.05 |
| 36 | I200 | Unstable angina | IX | 0.05 |
| 37 | Z82 | Family history of certain disabilities and chronic diseases leading to disablement | XXI | 0.05 |
| 38 | E10-E14 | Diabetes mellitus | IV | 0.05 |
| 39 | IV | Endocrine, nutritional and metabolic diseases | IV | 0.05 |
| 40 | E11 | Type 2 diabetes mellitus | IV | 0.05 |
| 41 | Y40-Y84 | Complications of medical and surgical care | XX | 0.05 |
| 42 | Y83-Y84 | Surgical and other medical procedures as the cause of abnormal reaction of the patient, or of later complication, without mention of misadventure at the time of the procedure | XX | 0.04 |
| 43 | M17 | Gonarthrosis [arthrosis of knee] | XIII | 0.04 |
| 44 | M15-M19 | Arthrosis | XIII | 0.04 |
| 45 | Z95 | Presence of cardiac and vascular implants and grafts | XXI | 0.04 |
| 46 | N0835 | Glomerular disorders in diabetes mellitus, chronic kidney disease, stage 5 | XIV | 0.04 |
| 47 | E1152 | Type 2 diabetes mellitus with certain circulatory complications | IV | 0.03 |
| 48 | Z540 | Convalescence following surgery | XXI | 0.03 |
| 49 | I20-I25 | Ischaemic heart diseases | IX | 0.03 |
| 50 | I25 | Chronic ischaemic heart disease | IX | 0.03 |
| 51 | E1123 | Type 2 diabetes mellitus with established or advanced kidney disease | IV | 0.03 |
| 52 | I4890 | Atrial fibrillation, unspecified | IX | 0.03 |
| 53 | I489 | Atrial fibrillation and atrial flutter, unspecified | IX | 0.03 |
| 54 | Z720 | Tobacco use | XXI | 0.03 |
| 55 | R9431 | Abnormal cardiovascular function studies (biomarkers or ECG) suggestive of non st segment elevation myocardial infarction [NSTEMI] | XVIII | 0.02 |
| 56 | M16 | Coxarthrosis [arthrosis of hip] | XIII | 0.02 |
| 57 | T80-T88 | Complications of surgical and medical care, not elsewhere classified | XIX | 0.02 |
| 58 | E1128 | Type 2 diabetes mellitus with other specified kidney complication not elsewhere classified | IV | 0.02 |
| 59 | T81 | Complications of procedures, not elsewhere classified | XIX | 0.02 |
| 60 | J128 | Other viral pneumonia | X | 0.02 |
| 61 | T82 | Complications of cardiac and vascular prosthetic devices, implants and grafts | XIX | 0.02 |
| 62 | Z8642 | Personal history of tobacco abuse | XXI | 0.02 |
| 63 | R9430 | Electrocardiogram suggestive of st segment elevation myocardial infarction [STEMI] | XVIII | 0.02 |
| 64 | N083 | Glomerular disorders in diabetes mellitus | XIV | 0.02 |
| 65 | I2088 | Other forms of angina pectoris | IX | 0.02 |
| 66 | N201 | Calculus of ureter | XIV | 0.02 |
| 67 | I208 | Other forms of angina pectoris | IX | 0.01 |
| 68 | C67 | Malignant neoplasm of bladder | II | 0.01 |
| 69 | H33 | Retinal detachments and breaks | VII | 0.01 |
| 70 | N0839 | Unspecified glomerular disorders in diabetes mellitus | XIV | 0.01 |
| 71 | M4806 | Spinal stenosis, lumbar region | XIII | 0.01 |
| 72 | S72 | Fracture of femur | XIX | 0.01 |
| 73 | H43-H45 | Disorders of vitreous body and globe | VII | 0.01 |
| 74 | K35-K38 | Diseases of appendix | XI | 0.01 |
| 75 | N00-N08 | Glomerular diseases | XIV | 0.01 |
| 76 | G55 | Nerve root and plexus compressions in diseases classified elsewhere | VI | 0.01 |
| 77 | M179 | Gonarthrosis, unspecified | XIII | 0.01 |
| 78 | E102 | Type 1 diabetes mellitus with renal complications | IV | 0.01 |
| 79 | E1023 | Type 1 diabetes mellitus with established or advanced kidney disease | IV | 0.0 |
| 80 | M170 | Primary gonarthrosis, bilateral | XIII | 0.0 |
| 81 | N08 | Glomerular disorders in diseases classified elsewhere | XIV | 0.0 |
| 82 | Z824 | Family history of ischaemic heart disease and other diseases of the circulatory system | XXI | 0.0 |
| 83 | M169 | Coxarthrosis, unspecified | XIII | 0.0 |
| 84 | K35 | Acute appendicitis | XI | 0.0 |
| 85 | G551 | Nerve root and plexus compressions in intervertebral disc disorders | VI | 0.0 |
| 86 | G553 | Nerve root and plexus compressions in other dorsopathies | VI | 0.0 |
| 87 | M471 | Other spondylosis with myelopathy | XIII | 0.0 |
| 88 | N993 | Prolapse of vaginal vault after hysterectomy | XIV | 0.0 |
| 89 | M511 | Lumbar and other intervertebral disc disorders with radiculopathy | XIII | 0.0 |
| 90 | E1433 | Unspecified diabetes mellitus with other retinopathy | IV | 0.0 |
| 91 | M501 | Cervical disc disorder with radiculopathy | XIII | 0.0 |
| 92 | Z37 | Outcome of delivery | XXI | 0.0 |
| 93 | 82463 | Neuroendocrine carcinoma | NEOPLASM | 0.0 |
| 94 | M4712 | Other spondylosis with myelopathy, cervical region | XIII | 0.0 |
| 95 | K359 | Acute appendicitis, unspecified | XI | 0.0 |
| 96 | H43 | Disorders of vitreous body | VII | 0.0 |
| 97 | H330 | Retinal detachment with retinal break | VII | 0.0 |
| 98 | H431 | Vitreous haemorrhage | VII | 0.0 |
| 99 | Z370 | Single live birth | XXI | 0.0 |
|  |  |  |  |  |

**Table S7. Features Selected by CAEWW in the NACRS Dataset**

|  | **Code** | **Description** | **Chapter** | **Mean Absolute SHAP Score** |
| --- | --- | --- | --- | --- |
| 0 | I30-I52 | Other forms of heart disease | IX | 0.36 |
| 1 | X | Diseases of the respiratory system | X | 0.19 |
| 2 | XVIII | Symptoms, signs and abnormal clinical and laboratory findings, not elsewhere classified | XVIII | 0.17 |
| 3 | II | Neoplasms | II | 0.16 |
| 4 | I51 | Complications and ill-defined descriptions of heart disease | IX | 0.14 |
| 5 | E11 | Type 2 diabetes mellitus | IV | 0.14 |
| 6 | N17-N19 | Renal failure | XIV | 0.12 |
| 7 | IX | Diseases of the circulatory system | IX | 0.1 |
| 8 | I50 | Heart failure | IX | 0.09 |
| 9 | R90-R94 | Abnormal findings on diagnostic imaging and in function studies, without diagnosis | XVIII | 0.09 |
| 10 | Z50 | Care involving use of rehabilitation procedures | XXI | 0.09 |
| 11 | E78 | Disorders of lipoprotein metabolism and other lipidaemias | IV | 0.08 |
| 12 | W00-W19 | Falls | XX | 0.08 |
| 13 | R07 | Pain in throat and chest | XVIII | 0.08 |
| 14 | Z501 | Other physical therapy | XXI | 0.08 |
| 15 | Z09 | Follow-up examination after treatment for conditions other than malignant neoplasms | XXI | 0.07 |
| 16 | R50-R69 | General symptoms and signs | XVIII | 0.06 |
| 17 | XI | Diseases of the digestive system | XI | 0.06 |
| 18 | Z01 | Other special examinations and investigations of persons without complaint or reported diagnosis | XXI | 0.06 |
| 19 | I49 | Other cardiac arrhythmias | IX | 0.06 |
| 20 | I10 | Essential (primary) hypertension | IX | 0.06 |
| 21 | XIV | Diseases of the genitourinary system | XIV | 0.05 |
| 22 | E70-E90 | Metabolic disorders | IV | 0.05 |
| 23 | VII | Diseases of the eye and adnexa | VII | 0.05 |
| 24 | Z55-Z65 | Persons with potential health hazards related to socioeconomic and psychosocial circumstances | XXI | 0.05 |
| 25 | I48 | Atrial fibrillation and flutter | IX | 0.05 |
| 26 | M00-M25 | Arthropathies | XIII | 0.05 |
| 27 | Z49 | Care involving dialysis | XXI | 0.05 |
| 28 | Z51 | Other medical care | XXI | 0.05 |
| 29 | I25 | Chronic ischaemic heart disease | IX | 0.05 |
| 30 | H25-H28 | Disorders of lens | VII | 0.05 |
| 31 | Z018 | Other specified special examinations | XXI | 0.05 |
| 32 | I20 | Angina pectoris | IX | 0.04 |
| 33 | Z718 | Other specified counselling | XXI | 0.04 |
| 34 | V | Mental and behavioural disorders | V | 0.04 |
| 35 | Z70-Z76 | Persons encountering health services in other circumstances | XXI | 0.04 |
| 36 | Z80-Z99 | Persons with potential health hazards related to family and personal history and certain conditions influencing health status | XXI | 0.04 |
| 37 | I | Certain infectious and parasitic diseases | I | 0.04 |
| 38 | E115 | Type 2 diabetes mellitus with peripheral circulatory complications | IV | 0.04 |
| 39 | J09-J18 | Influenza and pneumonia | X | 0.04 |
| 40 | Z13 | Special screening examination for other diseases and disorders | XXI | 0.04 |
| 41 | Z713 | Dietary counselling and surveillance | XXI | 0.04 |
| 42 | Z47 | Other orthopaedic follow-up care | XXI | 0.04 |
| 43 | Z03 | Medical observation and evaluation for suspected diseases and conditions, ruled out | XXI | 0.04 |
| 44 | E14 | Unspecified diabetes mellitus | IV | 0.03 |
| 45 | Z12 | Special screening examination for neoplasms | XXI | 0.03 |
| 46 | Z45 | Adjustment and management of implanted device | XXI | 0.03 |
| 47 | G40-G47 | Episodic and paroxysmal disorders | VI | 0.03 |
| 48 | D10-D36 | Benign neoplasms | II | 0.03 |
| 49 | I60-I69 | Cerebrovascular diseases | IX | 0.03 |
| 50 | Z40-Z54 | Persons encountering health services for specific procedures and health care | XXI | 0.03 |
| 51 | I251 | Atherosclerotic heart disease | IX | 0.03 |
| 52 | M60-M79 | Soft tissue disorders | XIII | 0.03 |
| 53 | Z48 | Other surgical follow-up care | XXI | 0.03 |
| 54 | K57 | Diverticular disease of intestine | XI | 0.03 |
| 55 | K20-K31 | Diseases of oesophagus, stomach and duodenum | XI | 0.03 |
| 56 | V01-X59 | Accidents | XX | 0.02 |
| 57 | S60-S69 | Injuries to the wrist and hand | XIX | 0.02 |
| 58 | I509 | Heart failure, unspecified | IX | 0.02 |
| 59 | R00-R09 | Symptoms and signs involving the circulatory and respiratory systems | XVIII | 0.02 |
| 60 | R10-R19 | Symptoms and signs involving the digestive system and abdomen | XVIII | 0.02 |
| 61 | J40-J47 | Chronic lower respiratory diseases | X | 0.02 |
| 62 | Z95 | Presence of cardiac and vascular implants and grafts | XXI | 0.02 |
| 63 | K63 | Other diseases of intestine | XI | 0.02 |
| 64 | W20-W49 | Exposure to inanimate mechanical forces | XX | 0.02 |
| 65 | XIX | Injury, poisoning and certain other consequences of external causes | XIX | 0.02 |
| 66 | Y83 | Surgical operation and other surgical procedures as the cause of abnormal reaction of the patient, or of later complication, without mention of misadventure at the time of the procedure | XX | 0.02 |
| 67 | III | Diseases of the blood and blood-forming organs and certain disorders involving the immune mechanism | III | 0.02 |
| 68 | T80-T88 | Complications of surgical and medical care, not elsewhere classified | XIX | 0.02 |
| 69 | E10 | Type 1 diabetes mellitus | IV | 0.02 |
| 70 | XII | Diseases of the skin and subcutaneous tissue | XII | 0.02 |
| 71 | K55-K64 | Other diseases of intestines | XI | 0.02 |
| 72 | VIII | Diseases of the ear and mastoid process | VIII | 0.02 |
| 73 | M40-M54 | Dorsopathies | XIII | 0.02 |
| 74 | Y40-Y84 | Complications of medical and surgical care | XX | 0.02 |
| 75 | K929 | Disease of digestive system, unspecified | XI | 0.02 |
| 76 | K90-K93 | Other diseases of the digestive system | XI | 0.01 |
| 77 | I20-I25 | Ischaemic heart diseases | IX | 0.01 |
| 78 | N18 | Chronic kidney disease | XIV | 0.01 |
| 79 | Z08 | Follow-up examination after treatment for malignant neoplasms | XXI | 0.01 |
| 80 | R40-R46 | Symptoms and signs involving cognition, perception, emotional state and behaviour | XVIII | 0.01 |
| 81 | I21 | Acute myocardial infarction | IX | 0.01 |
| 82 | VI | Diseases of the nervous system | VI | 0.01 |
| 83 | I80-I89 | Diseases of veins, lymphatic vessels and lymph nodes, not elsewhere classified | IX | 0.01 |
| 84 | R30-R39 | Symptoms and signs involving the urinary system | XVIII | 0.01 |
| 85 | Y83-Y84 | Surgical and other medical procedures as the cause of abnormal reaction of the patient, or of later complication, without mention of misadventure at the time of the procedure | XX | 0.01 |
| 86 | N30-N39 | Other diseases of urinary system | XIV | 0.01 |
| 87 | N28 | Other disorders of kidney and ureter, not elsewhere classified | XIV | 0.01 |
| 88 | I70-I79 | Diseases of arteries, arterioles and capillaries | IX | 0.01 |
| 89 | J95-J99 | Other diseases of the respiratory system | X | 0.01 |
| 90 | Z85 | Personal history of malignant neoplasm | XXI | 0.01 |
| 91 | H30-H36 | Disorders of choroid and retina | VII | 0.01 |
| 92 | M80-M94 | Osteopathies and chondropathies | XIII | 0.01 |
| 93 | K40-K46 | Hernia | XI | 0.01 |
| 94 | K80-K87 | Disorders of gallbladder, biliary tract and pancreas | XI | 0.0 |
|  |  |  |  |  |

**Table S8. Features Selected by CAENW in the NACRS Dataset**

|  | **Code** | **Description** | **Chapter** | **Mean Absolute SHAP Score** |
| --- | --- | --- | --- | --- |
| 0 | I30-I52 | Other forms of heart disease | IX | 0.34 |
| 1 | XVIII | Symptoms, signs and abnormal clinical and laboratory findings, not elsewhere classified | XVIII | 0.24 |
| 2 | X | Diseases of the respiratory system | X | 0.18 |
| 3 | IV | Endocrine, nutritional and metabolic diseases | IV | 0.16 |
| 4 | I51 | Complications and ill-defined descriptions of heart disease | IX | 0.14 |
| 5 | C00-C97 | Malignant neoplasms | II | 0.12 |
| 6 | I50 | Heart failure | IX | 0.12 |
| 7 | N17-N19 | Renal failure | XIV | 0.12 |
| 8 | IX | Diseases of the circulatory system | IX | 0.1 |
| 9 | R00-R09 | Symptoms and signs involving the circulatory and respiratory systems | XVIII | 0.1 |
| 10 | E78 | Disorders of lipoprotein metabolism and other lipidaemias | IV | 0.09 |
| 11 | W00-W19 | Falls | XX | 0.08 |
| 12 | Z01 | Other special examinations and investigations of persons without complaint or reported diagnosis | XXI | 0.07 |
| 13 | E11 | Type 2 diabetes mellitus | IV | 0.07 |
| 14 | Z09 | Follow-up examination after treatment for conditions other than malignant neoplasms | XXI | 0.07 |
| 15 | Z509 | Care involving use of rehabilitation procedure, unspecified | XXI | 0.07 |
| 16 | Z501 | Other physical therapy | XXI | 0.07 |
| 17 | I10-I15 | Hypertensive diseases | IX | 0.06 |
| 18 | VII | Diseases of the eye and adnexa | VII | 0.06 |
| 19 | I48 | Atrial fibrillation and flutter | IX | 0.06 |
| 20 | Z49 | Care involving dialysis | XXI | 0.05 |
| 21 | Z80-Z99 | Persons with potential health hazards related to family and personal history and certain conditions influencing health status | XXI | 0.05 |
| 22 | XI | Diseases of the digestive system | XI | 0.05 |
| 23 | Z03 | Medical observation and evaluation for suspected diseases and conditions, ruled out | XXI | 0.05 |
| 24 | H25-H28 | Disorders of lens | VII | 0.05 |
| 25 | M00-M25 | Arthropathies | XIII | 0.05 |
| 26 | Z713 | Dietary counselling and surveillance | XXI | 0.05 |
| 27 | XIV | Diseases of the genitourinary system | XIV | 0.05 |
| 28 | I20-I25 | Ischaemic heart diseases | IX | 0.05 |
| 29 | Z55-Z65 | Persons with potential health hazards related to socioeconomic and psychosocial circumstances | XXI | 0.05 |
| 30 | I | Certain infectious and parasitic diseases | I | 0.05 |
| 31 | I49 | Other cardiac arrhythmias | IX | 0.04 |
| 32 | R94 | Abnormal results of function studies | XVIII | 0.04 |
| 33 | Z13 | Special screening examination for other diseases and disorders | XXI | 0.04 |
| 34 | Z51 | Other medical care | XXI | 0.04 |
| 35 | E70-E90 | Metabolic disorders | IV | 0.04 |
| 36 | Z50 | Care involving use of rehabilitation procedures | XXI | 0.04 |
| 37 | V | Mental and behavioural disorders | V | 0.04 |
| 38 | I2510 | Atherosclerotic heart disease of native coronary artery | IX | 0.04 |
| 39 | E14 | Unspecified diabetes mellitus | IV | 0.04 |
| 40 | G40-G47 | Episodic and paroxysmal disorders | VI | 0.04 |
| 41 | J09-J18 | Influenza and pneumonia | X | 0.04 |
| 42 | Z45 | Adjustment and management of implanted device | XXI | 0.03 |
| 43 | Z12 | Special screening examination for neoplasms | XXI | 0.03 |
| 44 | Z40-Z54 | Persons encountering health services for specific procedures and health care | XXI | 0.03 |
| 45 | E115 | Type 2 diabetes mellitus with peripheral circulatory complications | IV | 0.03 |
| 46 | Z47 | Other orthopaedic follow-up care | XXI | 0.03 |
| 47 | I60-I69 | Cerebrovascular diseases | IX | 0.03 |
| 48 | Z71 | Persons encountering health services for other counselling and medical advice, not elsewhere classified | XXI | 0.03 |
| 49 | Z70-Z76 | Persons encountering health services in other circumstances | XXI | 0.03 |
| 50 | M60-M79 | Soft tissue disorders | XIII | 0.03 |
| 51 | K20-K31 | Diseases of oesophagus, stomach and duodenum | XI | 0.03 |
| 52 | I251 | Atherosclerotic heart disease | IX | 0.03 |
| 53 | XIX | Injury, poisoning and certain other consequences of external causes | XIX | 0.03 |
| 54 | Y83 | Surgical operation and other surgical procedures as the cause of abnormal reaction of the patient, or of later complication, without mention of misadventure at the time of the procedure | XX | 0.02 |
| 55 | I509 | Heart failure, unspecified | IX | 0.02 |
| 56 | S60-S69 | Injuries to the wrist and hand | XIX | 0.02 |
| 57 | R10 | Abdominal and pelvic pain | XVIII | 0.02 |
| 58 | T80-T88 | Complications of surgical and medical care, not elsewhere classified | XIX | 0.02 |
| 59 | Y40-Y84 | Complications of medical and surgical care | XX | 0.02 |
| 60 | J40-J47 | Chronic lower respiratory diseases | X | 0.02 |
| 61 | R90-R94 | Abnormal findings on diagnostic imaging and in function studies, without diagnosis | XVIII | 0.02 |
| 62 | W20-W49 | Exposure to inanimate mechanical forces | XX | 0.02 |
| 63 | XII | Diseases of the skin and subcutaneous tissue | XII | 0.02 |
| 64 | Z95 | Presence of cardiac and vascular implants and grafts | XXI | 0.02 |
| 65 | R688 | Other specified general symptoms and signs | XVIII | 0.02 |
| 66 | C00-C75 | Malignant neoplasms, stated or presumed to be primary, of specified sites, except of lymphoid, haematopoietic and related tissue | II | 0.02 |
| 67 | V01-X59 | Accidents | XX | 0.02 |
| 68 | D10-D36 | Benign neoplasms | II | 0.02 |
| 69 | R10-R19 | Symptoms and signs involving the digestive system and abdomen | XVIII | 0.02 |
| 70 | XIII | Diseases of the musculoskeletal system and connective tissue | XIII | 0.02 |
| 71 | N40-N51 | Diseases of male genital organs | XIV | 0.02 |
| 72 | II | Neoplasms | II | 0.02 |
| 73 | VIII | Diseases of the ear and mastoid process | VIII | 0.02 |
| 74 | R30-R39 | Symptoms and signs involving the urinary system | XVIII | 0.01 |
| 75 | III | Diseases of the blood and blood-forming organs and certain disorders involving the immune mechanism | III | 0.01 |
| 76 | K64 | Haemorrhoids and perianal venous thrombosis | XI | 0.01 |
| 77 | N28 | Other disorders of kidney and ureter, not elsewhere classified | XIV | 0.01 |
| 78 | K90-K93 | Other diseases of the digestive system | XI | 0.01 |
| 79 | N00-N08 | Glomerular diseases | XIV | 0.01 |
| 80 | K55-K64 | Other diseases of intestines | XI | 0.01 |
| 81 | R9431 | Abnormal cardiovascular function studies (biomarkers or ECG) suggestive of non st segment elevation myocardial infarction [NSTEMI] | XVIII | 0.01 |
| 82 | VI | Diseases of the nervous system | VI | 0.01 |
| 83 | E10 | Type 1 diabetes mellitus | IV | 0.01 |
| 84 | N30-N39 | Other diseases of urinary system | XIV | 0.01 |
| 85 | D64 | Other anaemias | III | 0.01 |
| 86 | M54 | Dorsalgia | XIII | 0.01 |
| 87 | M40-M54 | Dorsopathies | XIII | 0.01 |
| 88 | Z080 | Follow-up examination after surgery for malignant neoplasm | XXI | 0.01 |
| 89 | I70-I79 | Diseases of arteries, arterioles and capillaries | IX | 0.01 |
| 90 | A00-A09 | Intestinal infectious diseases | I | 0.01 |
| 91 | L00-L08 | Infections of the skin and subcutaneous tissue | XII | 0.01 |
| 92 | M20-M25 | Other joint disorders | XIII | 0.01 |
| 93 | Z08 | Follow-up examination after treatment for malignant neoplasms | XXI | 0.01 |
| 94 | H30-H36 | Disorders of choroid and retina | VII | 0.01 |
| 95 | Z85 | Personal history of malignant neoplasm | XXI | 0.01 |
| 96 | I21 | Acute myocardial infarction | IX | 0.01 |
|  |  |  |  |  |

**Table S9. Features Selected by AEFS in the NACRS Dataset**

|  | **Code** | **Description** | **Chapter** | **Mean Absolute SHAP Score** |
| --- | --- | --- | --- | --- |
| 0 | R90-R94 | Abnormal findings on diagnostic imaging and in function studies, without diagnosis | XVIII | 0.12 |
| 1 | E145 | Unspecified diabetes mellitus with peripheral circulatory complications | IV | 0.06 |
| 2 | K635 | Polyp of colon | XI | 0.03 |
| 3 | M75 | Shoulder lesions | XIII | 0.02 |
| 4 | J128 | Other viral pneumonia | X | 0.01 |
| 5 | J20-J22 | Other acute lower respiratory infections | X | 0.0 |
| 6 | E113 | Type 2 diabetes mellitus with ophthalmic complications | IV | 0.0 |
| 7 | I359 | Aortic valve disorder, unspecified | IX | 0.0 |
| 8 | Z59 | Problems related to housing and economic circumstances | XXI | 0.0 |
| 9 | Z092 | Follow-up examination after chemotherapy for other conditions | XXI | 0.0 |
| 10 | Y60-Y69 | Misadventures to patients during surgical and medical care | XX | 0.0 |
| 11 | M2588 | Other specified joint disorders, other site | XIII | 0.0 |
| 12 | C00-C14 | Malignant neoplasms of lip, oral cavity and pharynx | II | 0.0 |
| 13 | X10-X19 | Contact with heat and hot substances | XX | 0.0 |
| 14 | B02 | Zoster [herpes zoster] | I | 0.0 |
| 15 | L270 | Generalized skin eruption due to drugs and medicaments | XII | 0.0 |
| 16 | I12 | Hypertensive renal disease | IX | 0.0 |
| 17 | H905 | Sensorineural hearing loss, unspecified | VIII | 0.0 |
| 18 | C44 | Other malignant neoplasms of skin | II | 0.0 |
| 19 | R3911 | Poor urinary stream | XVIII | 0.0 |
| 20 | R298 | Other and unspecified symptoms and signs involving the nervous and musculoskeletal systems | XVIII | 0.0 |
| 21 | S8190 | Open wound of lower leg, part unspecified, uncomplicated | XIX | 0.0 |
| 22 | D759 | Disease of blood and blood-forming organs, unspecified | III | 0.0 |
| 23 | N0838 | Other glomerular disorders in diabetes mellitus | XIV | 0.0 |
| 24 | G25 | Other extrapyramidal and movement disorders | VI | 0.0 |
| 25 | C781 | Secondary malignant neoplasm of mediastinum | II | 0.0 |
| 26 | C240 | Malignant neoplasm: Extrahepatic bile duct | II | 0.0 |
| 27 | G57 | Mononeuropathies of lower limb | VI | 0.0 |
| 28 | I958 | Other hypotension | IX | 0.0 |
| 29 | H521 | Myopia | VII | 0.0 |
| 30 | A048 | Other specified bacterial intestinal infections | I | 0.0 |
| 31 | M49 | Spondylopathies in diseases classified elsewhere | XIII | 0.0 |
| 32 | B374 | Candidiasis of other urogenital sites | I | 0.0 |
| 33 | Y82 | Other and unspecified medical devices associated with adverse incidents | XX | 0.0 |
| 34 | B36 | Other superficial mycoses | I | 0.0 |
| 35 | L88 | Pyoderma gangrenosum | XII | 0.0 |
| 36 | E1432 | Unspecified diabetes mellitus with proliferative retinopathy | IV | 0.0 |
| 37 | I330 | Acute and subacute infective endocarditis | IX | 0.0 |
| 38 | J10 | Influenza due to identified seasonal influenza virus | X | 0.0 |
| 39 | S8698 | Other and unspecified injury of unspecified muscle and tendon at lower leg level | XIX | 0.0 |
| 40 | S82800 | Bimalleolar fracture of ankle, closed | XIX | 0.0 |
| 41 | M7116 | Other infective bursitis, lower leg | XIII | 0.0 |
| 42 | S12000 | Fracture of first cervical vertebra, closed | XIX | 0.0 |
| 43 | Q03 | Congenital hydrocephalus | XVII | 0.0 |
| 44 | B9548 | Other streptococcus as the cause of diseases classified to other chapters | I | 0.0 |
| 45 | F639 | Habit and impulse disorder, unspecified | V | 0.0 |
| 46 | D56 | Thalassaemia | III | 0.0 |
| 47 | M462 | Osteomyelitis of vertebra | XIII | 0.0 |
| 48 | M2429 | Disorder of ligament, unspecified site | XIII | 0.0 |
| 49 | S8170 | Multiple open wounds of lower leg, uncomplicated | XIX | 0.0 |
| 50 | T369 | Poisoning: Systemic antibiotic, unspecified | XIX | 0.0 |
| 51 | S410 | Open wound of shoulder | XIX | 0.0 |
| 52 | Y580 | BCG vaccine | XX | 0.0 |
| 53 | V483 | Car occupant injured in noncollision transport accident : unspecified car occupant injured in nontraffic accident | XX | 0.0 |
| 54 | S119 | Open wound of neck, part unspecified | XIX | 0.0 |
| 55 | S36800 | Haematoma of other intra-abdominal organs without open wound into cavity | XIX | 0.0 |
| 56 | S62320 | Fracture of head and neck of other metacarpal bone, closed | XIX | 0.0 |
| 57 | S53100 | Anterior dislocation of elbow, closed | XIX | 0.0 |
| 58 | V280 | Motorcycle rider injured in noncollision transport accident : driver injured in nontraffic accident | XX | 0.0 |
| 59 | T0130 | Open wounds of multiple regions of lower limb(s), uncomplicated | XIX | 0.0 |
| 60 | Z550 | Illiteracy and low-level literacy | XXI | 0.0 |
| 61 | V114 | Pedal cyclist injured in collision with other pedal cycle : driver injured in traffic accident | XX | 0.0 |
| 62 | T430 | Poisoning: Tricyclic and tetracyclic antidepressants | XIX | 0.0 |
| 63 | X48 | Accidental poisoning by and exposure to pesticides | XX | 0.0 |
| 64 | Z122 | Special screening examination for neoplasm of respiratory organs | XXI | 0.0 |
| 65 | Y546 | Electrolytic, caloric and water-balance agents | XX | 0.0 |
| 66 | M1253 | Traumatic arthropathy, forearm | XIII | 0.0 |
| 67 | N469 | Unspecified male infertility | XIV | 0.0 |
| 68 | M8619 | Other acute osteomyelitis, unspecified site | XIII | 0.0 |
| 69 | B368 | Other specified superficial mycoses | I | 0.0 |
| 70 | B964 | Proteus (mirabilis)(morganii) as the cause of diseases classified to other chapters | I | 0.0 |
| 71 | D06 | Carcinoma in situ of cervix uteri | II | 0.0 |
| 72 | D3612 | Benign neoplasm of peripheral nerves and autonomic nervous system of lower limb, including hip | II | 0.0 |
| 73 | E1131 | Type 2 diabetes mellitus with preproliferative retinopathy | IV | 0.0 |
| 74 | E283 | Primary ovarian failure | IV | 0.0 |
| 75 | E539 | Vitamin B deficiency, unspecified | IV | 0.0 |
| 76 | F120 | Mental and behavioural disorders due to use of cannabinoids : acute intoxication | V | 0.0 |
| 77 | F454 | Persistent somatoform pain disorder | V | 0.0 |
| 78 | F84 | Pervasive developmental disorders | V | 0.0 |
| 79 | G129 | Spinal muscular atrophy, unspecified | VI | 0.0 |
| 80 | G902 | Horner syndrome | VI | 0.0 |
| 81 | I651 | Occlusion and stenosis of basilar artery | IX | 0.0 |
| 82 | J158 | Other bacterial pneumonia | X | 0.0 |
| 83 | K131 | Cheek and lip biting | XI | 0.0 |
| 84 | K460 | Unspecified abdominal hernia with obstruction, without gangrene | XI | 0.0 |
| 85 | K911 | Postgastric surgery syndromes | XI | 0.0 |
| 86 | L13 | Other bullous disorders | XII | 0.0 |
| 87 | L251 | Unspecified contact dermatitis due to drugs in contact with skin | XII | 0.0 |
| 88 | M351 | Other overlap syndromes | XIII | 0.0 |
| 89 | M485 | Collapsed vertebra, not elsewhere classified | XIII | 0.0 |
| 90 | M6227 | Ischaemic infarction of muscle, ankle and foot | XIII | 0.0 |
| 91 | M6262 | Muscle strain, upper arm | XIII | 0.0 |
| 92 | M6296 | Disorder of muscle, unspecified, lower leg | XIII | 0.0 |
| 93 | M7195 | Bursopathy, unspecified, pelvic region and thigh | XIII | 0.0 |
| 94 | M7266 | Necrotizing fasciitis, lower leg | XIII | 0.0 |
| 95 | M7921 | Neuralgia and neuritis, unspecified, shoulder region | XIII | 0.0 |
| 96 | Z918 | Personal history of other specified risk-factors, not elsewhere classified | XXI | 0.0 |
|  |  |  |  |  |

**Table S10. Features Selected by MCFS in the NACRS Dataset**

|  | **Code** | **Description** | **Chapter** | **Mean Absolute SHAP Score** |
| --- | --- | --- | --- | --- |
| 0 | I30-I52 | Other forms of heart disease | IX | 0.28 |
| 1 | IV | Endocrine, nutritional and metabolic diseases | IV | 0.22 |
| 2 | XVIII | Symptoms, signs and abnormal clinical and laboratory findings, not elsewhere classified | XVIII | 0.2 |
| 3 | II | Neoplasms | II | 0.15 |
| 4 | I500 | Congestive heart failure | IX | 0.14 |
| 5 | Z00-Z13 | Persons encountering health services for examination and investigation | XXI | 0.14 |
| 6 | I10-I15 | Hypertensive diseases | IX | 0.12 |
| 7 | N17-N19 | Renal failure | XIV | 0.12 |
| 8 | I519 | Heart disease, unspecified | IX | 0.12 |
| 9 | Z016 | Radiological examination, not elsewhere classified | XXI | 0.09 |
| 10 | VII | Diseases of the eye and adnexa | VII | 0.08 |
| 11 | I100 | Benign hypertension | IX | 0.08 |
| 12 | Z501 | Other physical therapy | XXI | 0.07 |
| 13 | E11 | Type 2 diabetes mellitus | IV | 0.07 |
| 14 | Z40-Z54 | Persons encountering health services for specific procedures and health care | XXI | 0.07 |
| 15 | Z51 | Other medical care | XXI | 0.07 |
| 16 | XIII | Diseases of the musculoskeletal system and connective tissue | XIII | 0.06 |
| 17 | Z50 | Care involving use of rehabilitation procedures | XXI | 0.06 |
| 18 | Z509 | Care involving use of rehabilitation procedure, unspecified | XXI | 0.06 |
| 19 | Z035 | Observation for other suspected cardiovascular diseases | XXI | 0.06 |
| 20 | V01-X59 | Accidents | XX | 0.06 |
| 21 | XXI | Factors influencing health status and contact with health services | XXI | 0.05 |
| 22 | J96 | Respiratory failure, not elsewhere classified | X | 0.05 |
| 23 | Z713 | Dietary counselling and surveillance | XXI | 0.05 |
| 24 | Z09 | Follow-up examination after treatment for conditions other than malignant neoplasms | XXI | 0.05 |
| 25 | XX | External causes of morbidity and mortality | XX | 0.05 |
| 26 | I10 | Essential (primary) hypertension | IX | 0.05 |
| 27 | Z659 | Problem related to unspecified psychosocial circumstances | XXI | 0.04 |
| 28 | Z955 | Presence of coronary angioplasty implant and graft | XXI | 0.04 |
| 29 | I208 | Other forms of angina pectoris | IX | 0.04 |
| 30 | IX | Diseases of the circulatory system | IX | 0.04 |
| 31 | E14 | Unspecified diabetes mellitus | IV | 0.04 |
| 32 | I499 | Cardiac arrhythmia, unspecified | IX | 0.04 |
| 33 | R94 | Abnormal results of function studies | XVIII | 0.04 |
| 34 | E119 | Type 2 diabetes mellitus without complications | IV | 0.04 |
| 35 | XIV | Diseases of the genitourinary system | XIV | 0.04 |
| 36 | Z72 | Problems related to lifestyle | XXI | 0.04 |
| 37 | V | Mental and behavioural disorders | V | 0.04 |
| 38 | Z491 | Extracorporeal dialysis | XXI | 0.03 |
| 39 | Z13 | Special screening examination for other diseases and disorders | XXI | 0.03 |
| 40 | Z500 | Cardiac rehabilitation | XXI | 0.03 |
| 41 | XI | Diseases of the digestive system | XI | 0.03 |
| 42 | R90-R94 | Abnormal findings on diagnostic imaging and in function studies, without diagnosis | XVIII | 0.03 |
| 43 | Z018 | Other specified special examinations | XXI | 0.03 |
| 44 | Z01 | Other special examinations and investigations of persons without complaint or reported diagnosis | XXI | 0.03 |
| 45 | Z718 | Other specified counselling | XXI | 0.03 |
| 46 | Z80-Z99 | Persons with potential health hazards related to family and personal history and certain conditions influencing health status | XXI | 0.03 |
| 47 | Z86 | Personal history of certain other diseases | XXI | 0.03 |
| 48 | VI | Diseases of the nervous system | VI | 0.02 |
| 49 | K92 | Other diseases of digestive system | XI | 0.02 |
| 50 | Z71 | Persons encountering health services for other counselling and medical advice, not elsewhere classified | XXI | 0.02 |
| 51 | N08 | Glomerular disorders in diseases classified elsewhere | XIV | 0.02 |
| 52 | Z098 | Follow-up examination after other treatment for other conditions | XXI | 0.02 |
| 53 | E115 | Type 2 diabetes mellitus with peripheral circulatory complications | IV | 0.02 |
| 54 | E10-E14 | Diabetes mellitus | IV | 0.02 |
| 55 | Z719 | Counselling, unspecified | XXI | 0.02 |
| 56 | H269 | Cataract, unspecified | VII | 0.02 |
| 57 | Z49 | Care involving dialysis | XXI | 0.02 |
| 58 | K64 | Haemorrhoids and perianal venous thrombosis | XI | 0.02 |
| 59 | XIX | Injury, poisoning and certain other consequences of external causes | XIX | 0.02 |
| 60 | X58-X59 | Accidental exposure to other and unspecified factors | XX | 0.02 |
| 61 | Z48 | Other surgical follow-up care | XXI | 0.02 |
| 62 | K929 | Disease of digestive system, unspecified | XI | 0.02 |
| 63 | R52 | Pain, not elsewhere classified | XVIII | 0.02 |
| 64 | J95-J99 | Other diseases of the respiratory system | X | 0.01 |
| 65 | Z76 | Persons encountering health services in other circumstances | XXI | 0.01 |
| 66 | I95-I99 | Other and unspecified disorders of the circulatory system | IX | 0.01 |
| 67 | E1178 | Type 2 diabetes mellitus with multiple other complications | IV | 0.01 |
| 68 | Z46 | Fitting and adjustment of other devices | XXI | 0.01 |
| 69 | M799 | Soft tissue disorder, unspecified | XIII | 0.01 |
| 70 | T80-T88 | Complications of surgical and medical care, not elsewhere classified | XIX | 0.01 |
| 71 | D75 | Other diseases of blood and blood-forming organs | III | 0.01 |
| 72 | Z712 | Person consulting for explanation of investigation findings | XXI | 0.01 |
| 73 | G45 | Transient cerebral ischaemic attacks and related syndromes | VI | 0.01 |
| 74 | Z507 | Occupational therapy and vocational rehabilitation, not elsewhere classified | XXI | 0.01 |
| 75 | N18 | Chronic kidney disease | XIV | 0.01 |
| 76 | Y40-Y84 | Complications of medical and surgical care | XX | 0.01 |
| 77 | Z20-Z29 | Persons with potential health hazards related to communicable diseases | XXI | 0.01 |
| 78 | E10 | Type 1 diabetes mellitus | IV | 0.01 |
| 79 | M069 | Rheumatoid arthritis, unspecified | XIII | 0.01 |
| 80 | E112 | Type 2 diabetes mellitus with renal complications | IV | 0.01 |
| 81 | Z4500 | Adjustment and management of cardiac pacemaker | XXI | 0.01 |
| 82 | I516 | Cardiovascular disease, unspecified | IX | 0.01 |
| 83 | M80-M94 | Osteopathies and chondropathies | XIII | 0.01 |
| 84 | E1128 | Type 2 diabetes mellitus with other specified kidney complication not elsewhere classified | IV | 0.01 |
| 85 | N189 | Chronic kidney disease, unspecified | XIV | 0.01 |
| 86 | N00-N08 | Glomerular diseases | XIV | 0.01 |
| 87 | Z940 | Kidney transplant status | XXI | 0.01 |
| 88 | J69 | Pneumonitis due to solids and liquids | X | 0.01 |
| 89 | I518 | Other ill-defined heart diseases | IX | 0.0 |
| 90 | I493 | Ventricular premature depolarization | IX | 0.0 |
| 91 | Z000 | General medical examination | XXI | 0.0 |
| 92 | N399 | Disorder of urinary system, unspecified | XIV | 0.0 |
| 93 | Z717 | Human immunodeficiency virus [HIV] counselling | XXI | 0.0 |
| 94 | D70-D77 | Other diseases of blood and blood-forming organs | III | 0.0 |
| 95 | I498 | Other specified cardiac arrhythmias | IX | 0.0 |
| 96 | I495 | Sick sinus syndrome | IX | 0.0 |
| 97 | I4900 | Ventricular fibrillation | IX | 0.0 |
| 98 | I491 | Atrial premature depolarization | IX | 0.0 |
| 99 | I490 | Ventricular fibrillation and flutter | IX | 0.0 |
|  |  |  |  |  |

**Table S11. Features Selected by PFA in the NACRS Dataset**

|  | **Code** | **Description** | **Chapter** | **Mean Absolute SHAP Score** |
| --- | --- | --- | --- | --- |
| 0 | Z800 | Family history of malignant neoplasm of digestive organs | XXI | 0.01 |
| 1 | Z038 | Observation for other suspected diseases and conditions | XXI | 0.0 |
| 2 | K44 | Diaphragmatic hernia | XI | 0.0 |
| 3 | M256 | Stiffness of joint, not elsewhere classified | XIII | 0.0 |
| 4 | E15-E16 | Other disorders of glucose regulation and pancreatic internal secretion | IV | 0.0 |
| 5 | E141 | Unspecified diabetes mellitus with ketoacidosis | IV | 0.0 |
| 6 | R52 | Pain, not elsewhere classified | XVIII | 0.0 |
| 7 | S460 | Injury of muscle(s) and tendon(s) of the rotator cuff of shoulder | XIX | 0.0 |
| 8 | J040 | Acute laryngitis | X | 0.0 |
| 9 | R011 | Cardiac murmur, unspecified | XVIII | 0.0 |
| 10 | I958 | Other hypotension | IX | 0.0 |
| 11 | A48 | Other bacterial diseases, not elsewhere classified | I | 0.0 |
| 12 | I260 | Pulmonary embolism with mention of acute cor pulmonale | IX | 0.0 |
| 13 | K523 | Indeterminate colitis | XI | 0.0 |
| 14 | K561 | Intussusception | XI | 0.0 |
| 15 | S16 | Injury of muscle and tendon at neck level | XIX | 0.0 |
| 16 | K822 | Perforation of gallbladder | XI | 0.0 |
| 17 | I425 | Other restrictive cardiomyopathy | IX | 0.0 |
| 18 | D83 | Common variable immunodeficiency | III | 0.0 |
| 19 | I256 | Silent myocardial ischaemia | IX | 0.0 |
| 20 | C97-C97 | Malignant neoplasms of independent (primary) multiple sites | II | 0.0 |
| 21 | E26 | Hyperaldosteronism | IV | 0.0 |
| 22 | N281 | Cyst of kidney | XIV | 0.0 |
| 23 | C82 | Follicular lymphoma | II | 0.0 |
| 24 | F70-F79 | Mental retardation | V | 0.0 |
| 25 | S82200 | Fracture of shaft of tibia with or without fibula, closed | XIX | 0.0 |
| 26 | A08 | Viral and other specified intestinal infections | I | 0.0 |
| 27 | G52 | Disorders of other cranial nerves | VI | 0.0 |
| 28 | T180 | Foreign body in mouth | XIX | 0.0 |
| 29 | J62 | Pneumoconiosis due to dust containing silica | X | 0.0 |
| 30 | R203 | Hyperaesthesia | XVIII | 0.0 |
| 31 | S52900 | Fracture of forearm, part unspecified, closed | XIX | 0.0 |
| 32 | B972 | Coronavirus as the cause of diseases classified to other chapters | I | 0.0 |
| 33 | C07 | Malignant neoplasm of parotid gland | II | 0.0 |
| 34 | S1438 | Other and unspecified injury of brachial plexus | XIX | 0.0 |
| 35 | C162 | Malignant neoplasm: Body of stomach | II | 0.0 |
| 36 | R416 | Neurological neglect syndrome | XVIII | 0.0 |
| 37 | F321 | Moderate depressive episode | V | 0.0 |
| 38 | B964 | Proteus (mirabilis)(morganii) as the cause of diseases classified to other chapters | I | 0.0 |
| 39 | N600 | Solitary cyst of breast | XIV | 0.0 |
| 40 | N423 | Dysplasia of prostate | XIV | 0.0 |
| 41 | N420 | Calculus of prostate | XIV | 0.0 |
| 42 | N303 | Trigonitis | XIV | 0.0 |
| 43 | C759 | Malignant neoplasm: Endocrine gland, unspecified | II | 0.0 |
| 44 | M843 | Stress fracture, not elsewhere classified | XIII | 0.0 |
| 45 | M7914 | Myalgia, hand | XIII | 0.0 |
| 46 | C505 | Malignant neoplasm: Lower-outer quadrant of breast | II | 0.0 |
| 47 | T115 | Injury of unspecified muscle and tendon of upper limb, level unspecified | XIX | 0.0 |
| 48 | M6592 | Synovitis and tenosynovitis, unspecified, upper arm | XIII | 0.0 |
| 49 | T54 | Toxic effect of corrosive substances | XIX | 0.0 |
| 50 | T66 | Unspecified effects of radiation | XIX | 0.0 |
| 51 | T8465 | Infection and inflammatory reaction due to internal fixation device of bones of foot | XIX | 0.0 |
| 52 | T864 | Liver transplant failure and rejection | XIX | 0.0 |
| 53 | T8738 | Neuroma of other amputation stump | XIX | 0.0 |
| 54 | T889 | Complication of surgical and medical care, unspecified | XIX | 0.0 |
| 55 | V02 | Pedestrian injured in collision with two- or three-wheeled motor vehicle | XX | 0.0 |
| 56 | V87 | Traffic accident of specified type but victim's mode of transport unknown | XX | 0.0 |
| 57 | V88 | Nontraffic accident of specified type but victim's mode of transport unknown | XX | 0.0 |
| 58 | Y410 | Sulfonamides | XX | 0.0 |
| 59 | Y780 | Radiological devices associated with adverse incidents : diagnostic and monitoring devices | XX | 0.0 |
| 60 | B027 | Disseminated zoster | I | 0.0 |
| 61 | A630 | Anogenital (venereal) warts | I | 0.0 |
| 62 | Z916 | Personal history of other physical trauma | XXI | 0.0 |
| 63 | M7191 | Bursopathy, unspecified, shoulder region | XIII | 0.0 |
| 64 | M484 | Fatigue fracture of vertebra | XIII | 0.0 |
| 65 | M6212 | Other rupture of muscle (nontraumatic), upper arm | XIII | 0.0 |
| 66 | E283 | Primary ovarian failure | IV | 0.0 |
| 67 | D486 | Neoplasm of uncertain or unknown behaviour: Breast | II | 0.0 |
| 68 | I840 | Internal thrombosed haemorrhoids | I | 0.0 |
| 69 | I673 | Progressive vascular leukoencephalopathy | IX | 0.0 |
| 70 | I658 | Occlusion and stenosis of other precerebral artery | IX | 0.0 |
| 71 | I613 | Intracerebral haemorrhage in brain stem | IX | 0.0 |
| 72 | E260 | Primary hyperaldosteronism | IV | 0.0 |
| 73 | E783 | Hyperchylomicronaemia | IV | 0.0 |
| 74 | J206 | Acute bronchitis due to rhinovirus | X | 0.0 |
| 75 | I150 | Renovascular hypertension | IX | 0.0 |
| 76 | H404 | Glaucoma secondary to eye inflammation | VII | 0.0 |
| 77 | H162 | Keratoconjunctivitis | VII | 0.0 |
| 78 | G723 | Periodic paralysis | VI | 0.0 |
| 79 | G578 | Other mononeuropathies of lower limb | VI | 0.0 |
| 80 | G06 | Intracranial and intraspinal abscess and granuloma | VI | 0.0 |
| 81 | D315 | Benign neoplasm: Lacrimal gland and duct | II | 0.0 |
| 82 | J91 | Pleural effusion in conditions classified elsewhere | X | 0.0 |
| 83 | F314 | Bipolar affective disorder, current episode severe depression without psychotic symptoms | V | 0.0 |
| 84 | K823 | Fistula of gallbladder | XI | 0.0 |
| 85 | M2586 | Other specified joint disorders, lower leg | XIII | 0.0 |
| 86 | D136 | Benign neoplasm: Pancreas | II | 0.0 |
| 87 | M2446 | Recurrent dislocation and subluxation of joint, lower leg | XIII | 0.0 |
| 88 | L52 | Erythema nodosum | XII | 0.0 |
| 89 | L449 | Papulosquamous disorder, unspecified | XII | 0.0 |
| 90 | L040 | Acute lymphadenitis of face, head and neck | XII | 0.0 |
| 91 | D151 | Benign neoplasm: Heart | II | 0.0 |
| 92 | K2210 | Ulcer of oesophagus, acute with haemorrhage | XI | 0.0 |
| 93 | K605 | Anorectal fistula | XI | 0.0 |
| 94 | K563 | Gallstone ileus | XI | 0.0 |
| 95 | D181 | Lymphangioma, any site | II | 0.0 |
| 96 | D227 | Melanocytic naevi of lower limb, including hip | II | 0.0 |
| 97 | A400 | Sepsis due to streptococcus, group A | I | 0.0 |
| 98 | K2213 | Ulcer of oesophagus, acute without haemorrhage or perforation | XI | 0.0 |
| 99 | Z9481 | Intestine transplant status | XXI | 0.0 |
|  |  |  |  |  |

**Table S12. Features Selected by LS in the NACRS Dataset**

|  | **Code** | **Description** | **Chapter** | **Mean Absolute SHAP Score** |
| --- | --- | --- | --- | --- |
| 0 | IV | Endocrine, nutritional and metabolic diseases | IV | 0.33 |
| 1 | I30-I52 | Other forms of heart disease | IX | 0.26 |
| 2 | Z00-Z13 | Persons encountering health services for examination and investigation | XXI | 0.23 |
| 3 | I50 | Heart failure | IX | 0.17 |
| 4 | W00-X59 | Other external causes of accidental injury | XX | 0.16 |
| 5 | I10-I15 | Hypertensive diseases | IX | 0.16 |
| 6 | Z016 | Radiological examination, not elsewhere classified | XXI | 0.15 |
| 7 | I100 | Benign hypertension | IX | 0.14 |
| 8 | Z40-Z54 | Persons encountering health services for specific procedures and health care | XXI | 0.14 |
| 9 | IX | Diseases of the circulatory system | IX | 0.12 |
| 10 | E10-E14 | Diabetes mellitus | IV | 0.1 |
| 11 | Z035 | Observation for other suspected cardiovascular diseases | XXI | 0.09 |
| 12 | R07 | Pain in throat and chest | XVIII | 0.08 |
| 13 | H25-H28 | Disorders of lens | VII | 0.08 |
| 14 | V01-X59 | Accidents | XX | 0.08 |
| 15 | E78 | Disorders of lipoprotein metabolism and other lipidaemias | IV | 0.08 |
| 16 | I51 | Complications and ill-defined descriptions of heart disease | IX | 0.08 |
| 17 | R90-R94 | Abnormal findings on diagnostic imaging and in function studies, without diagnosis | XVIII | 0.07 |
| 18 | I519 | Heart disease, unspecified | IX | 0.07 |
| 19 | I20 | Angina pectoris | IX | 0.06 |
| 20 | E11 | Type 2 diabetes mellitus | IV | 0.05 |
| 21 | N18 | Chronic kidney disease | XIV | 0.05 |
| 22 | E115 | Type 2 diabetes mellitus with peripheral circulatory complications | IV | 0.05 |
| 23 | Z71 | Persons encountering health services for other counselling and medical advice, not elsewhere classified | XXI | 0.05 |
| 24 | Z450 | Adjustment and management of cardiac devices | XXI | 0.05 |
| 25 | Z03 | Medical observation and evaluation for suspected diseases and conditions, ruled out | XXI | 0.05 |
| 26 | I499 | Cardiac arrhythmia, unspecified | IX | 0.05 |
| 27 | Z01 | Other special examinations and investigations of persons without complaint or reported diagnosis | XXI | 0.05 |
| 28 | XXI | Factors influencing health status and contact with health services | XXI | 0.04 |
| 29 | Z13 | Special screening examination for other diseases and disorders | XXI | 0.04 |
| 30 | I251 | Atherosclerotic heart disease | IX | 0.04 |
| 31 | Z50 | Care involving use of rehabilitation procedures | XXI | 0.03 |
| 32 | I25 | Chronic ischaemic heart disease | IX | 0.03 |
| 33 | Z70-Z76 | Persons encountering health services in other circumstances | XXI | 0.03 |
| 34 | I509 | Heart failure, unspecified | IX | 0.03 |
| 35 | Z121 | Special screening examination for neoplasm of intestinal tract | XXI | 0.03 |
| 36 | Z491 | Extracorporeal dialysis | XXI | 0.03 |
| 37 | I10 | Essential (primary) hypertension | IX | 0.03 |
| 38 | I20-I25 | Ischaemic heart diseases | IX | 0.02 |
| 39 | S61 | Open wound of wrist and hand | XIX | 0.02 |
| 40 | R9431 | Abnormal cardiovascular function studies (biomarkers or ECG) suggestive of non st segment elevation myocardial infarction [NSTEMI] | XVIII | 0.02 |
| 41 | N083 | Glomerular disorders in diabetes mellitus | XIV | 0.02 |
| 42 | H26 | Other cataract | VII | 0.02 |
| 43 | R074 | Chest pain, unspecified | XVIII | 0.02 |
| 44 | I214 | Acute subendocardial myocardial infarction | IX | 0.02 |
| 45 | X58-X59 | Accidental exposure to other and unspecified factors | XX | 0.02 |
| 46 | I2510 | Atherosclerotic heart disease of native coronary artery | IX | 0.02 |
| 47 | M799 | Soft tissue disorder, unspecified | XIII | 0.02 |
| 48 | Z136 | Special screening examination for cardiovascular disorders | XXI | 0.02 |
| 49 | R943 | Abnormal results of cardiovascular function studies | XVIII | 0.02 |
| 50 | N25-N29 | Other disorders of kidney and ureter | XIV | 0.02 |
| 51 | R80-R82 | Abnormal findings on examination of urine, without diagnosis | XVIII | 0.02 |
| 52 | J984 | Other disorders of lung | X | 0.01 |
| 53 | E1128 | Type 2 diabetes mellitus with other specified kidney complication not elsewhere classified | IV | 0.01 |
| 54 | Z860 | Personal history of other neoplasms | XXI | 0.01 |
| 55 | E112 | Type 2 diabetes mellitus with renal complications | IV | 0.01 |
| 56 | X59 | Exposure to unspecified factor | XX | 0.01 |
| 57 | M259 | Joint disorder, unspecified | XIII | 0.01 |
| 58 | I21 | Acute myocardial infarction | IX | 0.01 |
| 59 | R94 | Abnormal results of function studies | XVIII | 0.01 |
| 60 | W44 | Foreign body entering into or through eye or natural orifice | XX | 0.01 |
| 61 | N289 | Disorder of kidney and ureter, unspecified | XIV | 0.01 |
| 62 | Z4500 | Adjustment and management of cardiac pacemaker | XXI | 0.01 |
| 63 | Z94 | Transplanted organ and tissue status | XXI | 0.01 |
| 64 | E831 | Disorders of iron metabolism | IV | 0.01 |
| 65 | E1152 | Type 2 diabetes mellitus with certain circulatory complications | IV | 0.01 |
| 66 | N28 | Other disorders of kidney and ureter, not elsewhere classified | XIV | 0.01 |
| 67 | R195 | Other faecal abnormalities | XVIII | 0.01 |
| 68 | Z45 | Adjustment and management of implanted device | XXI | 0.01 |
| 69 | Z49 | Care involving dialysis | XXI | 0.01 |
| 70 | X599 | Exposure to unspecified factor causing other and unspecified injury | XX | 0.01 |
| 71 | M8999 | Disorder of bone, unspecified, unspecified site | XIII | 0.0 |
| 72 | H269 | Cataract, unspecified | VII | 0.0 |
| 73 | T143 | Dislocation, sprain and strain of unspecified body region | XIX | 0.0 |
| 74 | X20-X29 | Contact with venomous animals and plants | XX | 0.0 |
| 75 | M2588 | Other specified joint disorders, other site | XIII | 0.0 |
| 76 | E1070 | Type 1 diabetes mellitus with foot ulcer (angiopathic) (neuropathic) | IV | 0.0 |
| 77 | S6100 | Open wound of finger(s) without damage to nail, uncomplicated | XIX | 0.0 |
| 78 | M256 | Stiffness of joint, not elsewhere classified | XIII | 0.0 |
| 79 | T15 | Foreign body on external eye | XIX | 0.0 |
| 80 | M899 | Disorder of bone, unspecified | XIII | 0.0 |
| 81 | T14 | Injury of unspecified body region | XIX | 0.0 |
| 82 | M2598 | Joint disorder, unspecified, other site | XIII | 0.0 |
| 83 | T1420 | Fracture of unspecified body region, closed | XIX | 0.0 |
| 84 | Z8601 | Personal history of benign neoplasms | XXI | 0.0 |
| 85 | E8310 | Haemochromatosis | IV | 0.0 |
| 86 | R80 | Isolated proteinuria | XVIII | 0.0 |
| 87 | S610 | Open wound of finger(s) without damage to nail | XIX | 0.0 |
| 88 | F318 | Other bipolar affective disorders | V | 0.0 |
| 89 | R1950 | Positive faecal occult blood test | XVIII | 0.0 |
| 90 | F203 | Undifferentiated schizophrenia | V | 0.0 |
| 91 | C830 | Small cell B-cell lymphoma | II | 0.0 |
| 92 | G8111 | Spastic hemiplegia of non-dominant side | VI | 0.0 |
| 93 | M2429 | Disorder of ligament, unspecified site | XIII | 0.0 |
| 94 | M2569 | Stiffness of joint, not elsewhere classified, unspecified site | XIII | 0.0 |
| 95 | X23 | Contact with hornets, wasps and bees | XX | 0.0 |
| 96 | T634 | Toxic effect: Venom of other arthropods | XIX | 0.0 |
| 97 | T63 | Toxic effect of contact with venomous animals | XIX | 0.0 |
| 98 | T142 | Fracture of unspecified body region | XIX | 0.0 |
| 99 | M7998 | Soft tissue disorder, unspecified, other site | XIII | 0.0 |
|  |  |  |  |  |

**Table S13. Features Selected by CAEWW in the PIN Dataset**

|  | **Code** | **Description** | **Rank** | **Mean Absolute SHAP Score** |
| --- | --- | --- | --- | --- |
| 0 | C03C | HIGH-CEILING DIURETICS | 2.0 | 0.43 |
| 1 | C10AA | HMG CoA reductase inhibitors | 3.0 | 0.28 |
| 2 | C09A | ACE INHIBITORS, PLAIN | 2.0 | 0.21 |
| 3 | A02BC02 | pantoprazole | 4.0 | 0.2 |
| 4 | C09CA | Angiotensin II receptor blockers (ARBs), plain | 3.0 | 0.16 |
| 5 | M01 | ANTIINFLAMMATORY AND ANTIRHEUMATIC PRODUCTS | 1.0 | 0.16 |
| 6 | B01 | ANTITHROMBOTIC AGENTS | 1.0 | 0.15 |
| 7 | A06A | DRUGS FOR CONSTIPATION | 2.0 | 0.14 |
| 8 | N02A | OPIOIDS | 2.0 | 0.12 |
| 9 | R03A | ADRENERGICS, INHALANTS | 2.0 | 0.1 |
| 10 | C09D | ANGIOTENSIN II RECEPTOR BLOCKERS (ARBs), COMBINATIONS | 2.0 | 0.09 |
| 11 | H02 | CORTICOSTEROIDS FOR SYSTEMIC USE | 1.0 | 0.09 |
| 12 | N02B | OTHER ANALGESICS AND ANTIPYRETICS | 2.0 | 0.09 |
| 13 | N05A | ANTIPSYCHOTICS | 2.0 | 0.09 |
| 14 | B01AF | Direct factor Xa inhibitors | 3.0 | 0.08 |
| 15 | B03 | ANTIANEMIC PREPARATIONS | 1.0 | 0.08 |
| 16 | A02 | DRUGS FOR ACID RELATED DISORDERS | 1.0 | 0.08 |
| 17 | C01D | VASODILATORS USED IN CARDIAC DISEASES | 2.0 | 0.08 |
| 18 | G04B | UROLOGICALS | 2.0 | 0.07 |
| 19 | C10AX | Other lipid modifying agents | 3.0 | 0.07 |
| 20 | J07 | VACCINES | 1.0 | 0.07 |
| 21 | A10BA02 | metformin | 4.0 | 0.07 |
| 22 | A03 | DRUGS FOR FUNCTIONAL GASTROINTESTINAL DISORDERS | 1.0 | 0.07 |
| 23 | C03D | POTASSIUM-SPARING AGENTS | 2.0 | 0.07 |
| 24 | N03 | ANTIEPILEPTICS | 1.0 | 0.06 |
| 25 | A10A | INSULINS AND ANALOGUES | 2.0 | 0.06 |
| 26 | N06 | PSYCHOANALEPTICS | 1.0 | 0.06 |
| 27 | R03B | OTHER DRUGS FOR OBSTRUCTIVE AIRWAY DISEASES, INHALANTS | 2.0 | 0.06 |
| 28 | C09B | ACE INHIBITORS, COMBINATIONS | 2.0 | 0.05 |
| 29 | D07A | CORTICOSTEROIDS, PLAIN | 2.0 | 0.05 |
| 30 | G04CA | Alpha-adrenoreceptor antagonists | 3.0 | 0.05 |
| 31 | A12 | MINERAL SUPPLEMENTS | 1.0 | 0.05 |
| 32 | C07 | BETA BLOCKING AGENTS | 1.0 | 0.05 |
| 33 | L | ANTINEOPLASTIC AND IMMUNOMODULATING AGENTS | 0.0 | 0.05 |
| 34 | C03AA | Thiazides, plain | 3.0 | 0.05 |
| 35 | J01F | MACROLIDES, LINCOSAMIDES AND STREPTOGRAMINS | 2.0 | 0.05 |
| 36 | G03 | SEX HORMONES AND MODULATORS OF THE GENITAL SYSTEM | 1.0 | 0.04 |
| 37 | C08C | SELECTIVE CALCIUM CHANNEL BLOCKERS WITH MAINLY VASCULAR EFFECTS | 2.0 | 0.04 |
| 38 | C07AG | Alpha and beta blocking agents | 3.0 | 0.04 |
| 39 | J01C | BETA-LACTAM ANTIBACTERIALS, PENICILLINS | 2.0 | 0.04 |
| 40 | N02AA | Natural opium alkaloids | 3.0 | 0.04 |
| 41 | M | MUSCULO-SKELETAL SYSTEM | 0.0 | 0.04 |
| 42 | A04 | ANTIEMETICS AND ANTINAUSEANTS | 1.0 | 0.04 |
| 43 | C07AB02 | metoprolol | 4.0 | 0.04 |
| 44 | C10AB | Fibrates | 3.0 | 0.04 |
| 45 | R01 | NASAL PREPARATIONS | 1.0 | 0.03 |
| 46 | C08D | SELECTIVE CALCIUM CHANNEL BLOCKERS WITH DIRECT CARDIAC EFFECTS | 2.0 | 0.03 |
| 47 | M03 | MUSCLE RELAXANTS | 1.0 | 0.03 |
| 48 | N05C | HYPNOTICS AND SEDATIVES | 2.0 | 0.03 |
| 49 | C09AA04 | perindopril | 4.0 | 0.03 |
| 50 | A10BB | Sulfonylureas | 3.0 | 0.03 |
| 51 | J01D | OTHER BETA-LACTAM ANTIBACTERIALS | 2.0 | 0.03 |
| 52 | B01AC06 | acetylsalicylic acid | 4.0 | 0.03 |
| 53 | B01AC04 | clopidogrel | 4.0 | 0.03 |
| 54 | C10AA05 | atorvastatin | 4.0 | 0.03 |
| 55 | H03 | THYROID THERAPY | 1.0 | 0.03 |
| 56 | C09AA05 | ramipril | 4.0 | 0.02 |
| 57 | A11 | VITAMINS | 1.0 | 0.02 |
| 58 | B01AA | Vitamin K antagonists | 3.0 | 0.02 |
| 59 | S01AE | Fluoroquinolones | 3.0 | 0.02 |
| 60 | S02 | OTOLOGICALS | 1.0 | 0.02 |
| 61 | A07 | ANTIDIARRHEALS, INTESTINAL ANTIINFLAMMATORY/ANTIINFECTIVE AGENTS | 1.0 | 0.02 |
| 62 | C01B | ANTIARRHYTHMICS, CLASS I AND III | 2.0 | 0.02 |
| 63 | M05 | DRUGS FOR TREATMENT OF BONE DISEASES | 1.0 | 0.02 |
| 64 | R05 | COUGH AND COLD PREPARATIONS | 1.0 | 0.01 |
| 65 | A10BD | Combinations of oral blood glucose lowering drugs | 3.0 | 0.01 |
| 66 | D01 | ANTIFUNGALS FOR DERMATOLOGICAL USE | 1.0 | 0.01 |
| 67 | N06AB | Selective serotonin reuptake inhibitors | 3.0 | 0.01 |
| 68 | S | SENSORY ORGANS | 0.0 | 0.01 |
| 69 | J01MA | Fluoroquinolones | 3.0 | 0.01 |
| 70 | C01AA | Digitalis glycosides | 3.0 | 0.01 |
| 71 | A10BK | Sodium-glucose co-transporter 2 (SGLT2) inhibitors | 3.0 | 0.01 |
| 72 | P | ANTIPARASITIC PRODUCTS, INSECTICIDES AND REPELLENTS | 0.0 | 0.01 |
| 73 | A10BX | Other blood glucose lowering drugs, excl. insulins | 3.0 | 0.01 |
| 74 | D | DERMATOLOGICALS | 0.0 | 0.01 |
| 75 | N07 | OTHER NERVOUS SYSTEM DRUGS | 1.0 | 0.01 |
| 76 | N04 | ANTI-PARKINSON DRUGS | 1.0 | 0.01 |
| 77 | J01A | TETRACYCLINES | 2.0 | 0.01 |
| 78 | N07CA | Antivertigo preparations | 3.0 | 0.01 |
| 79 | J01E | SULFONAMIDES AND TRIMETHOPRIM | 2.0 | 0.01 |
| 80 | A10BH | Dipeptidyl peptidase 4 (DPP-4) inhibitors | 3.0 | 0.01 |
| 81 | M04A | ANTIGOUT PREPARATIONS | 2.0 | 0.01 |
| 82 | D05 | ANTIPSORIATICS | 1.0 | 0.01 |
| 83 | C03BA | Sulfonamides, plain | 3.0 | 0.01 |
| 84 | C03EA01 | hydrochlorothiazide and potassium-sparing agents | 4.0 | 0.01 |
| 85 | G04CB | Testosterone-5-alpha reductase inhibitors | 3.0 | 0.01 |
| 86 | R03D | OTHER SYSTEMIC DRUGS FOR OBSTRUCTIVE AIRWAY DISEASES | 2.0 | 0.01 |
| 87 | R06 | ANTIHISTAMINES FOR SYSTEMIC USE | 1.0 | 0.01 |
| 88 | M01AB | Acetic acid derivatives and related substances | 3.0 | 0.01 |
| 89 | N05B | ANXIOLYTICS | 2.0 | 0.01 |
| 90 | A02BA | H2-receptor antagonists | 3.0 | 0.01 |
| 91 | C02 | ANTIHYPERTENSIVES | 1.0 | 0.01 |
| 92 | A01 | STOMATOLOGICAL PREPARATIONS | 1.0 | 0.01 |
| 93 | V | VARIOUS | 0.0 | 0.01 |
| 94 | J05A | DIRECT ACTING ANTIVIRALS | 2.0 | 0.01 |
| 95 | S01E | ANTIGLAUCOMA PREPARATIONS AND MIOTICS | 2.0 | 0.01 |
| 96 | D06 | ANTIBIOTICS AND CHEMOTHERAPEUTICS FOR DERMATOLOGICAL USE | 1.0 | 0.01 |
| 97 | J01X | OTHER ANTIBACTERIALS | 2.0 | 0.0 |
| 98 | J02 | ANTIMYCOTICS FOR SYSTEMIC USE | 1.0 | 0.0 |
| 99 | C05 | VASOPROTECTIVES | 1.0 | 0.0 |
|  |  |  |  |  |

**Table S14. Features Selected by CAENW in the PIN Dataset**

|  | **Code** | **Description** | **Rank** | **Mean Absolute SHAP Score** |
| --- | --- | --- | --- | --- |
| 0 | C03C | HIGH-CEILING DIURETICS | 2.0 | 0.44 |
| 1 | C10AA05 | atorvastatin | 4.0 | 0.22 |
| 2 | C10AA07 | rosuvastatin | 4.0 | 0.21 |
| 3 | C09AA | ACE inhibitors, plain | 3.0 | 0.2 |
| 4 | M01 | ANTIINFLAMMATORY AND ANTIRHEUMATIC PRODUCTS | 1.0 | 0.16 |
| 5 | B01 | ANTITHROMBOTIC AGENTS | 1.0 | 0.15 |
| 6 | C09C | ANGIOTENSIN II RECEPTOR BLOCKERS (ARBs), PLAIN | 2.0 | 0.14 |
| 7 | A06A | DRUGS FOR CONSTIPATION | 2.0 | 0.14 |
| 8 | N02A | OPIOIDS | 2.0 | 0.14 |
| 9 | R03 | DRUGS FOR OBSTRUCTIVE AIRWAY DISEASES | 1.0 | 0.1 |
| 10 | B03 | ANTIANEMIC PREPARATIONS | 1.0 | 0.09 |
| 11 | C09D | ANGIOTENSIN II RECEPTOR BLOCKERS (ARBs), COMBINATIONS | 2.0 | 0.09 |
| 12 | B01AF | Direct factor Xa inhibitors | 3.0 | 0.09 |
| 13 | H02 | CORTICOSTEROIDS FOR SYSTEMIC USE | 1.0 | 0.09 |
| 14 | N05A | ANTIPSYCHOTICS | 2.0 | 0.09 |
| 15 | C01D | VASODILATORS USED IN CARDIAC DISEASES | 2.0 | 0.08 |
| 16 | N02B | OTHER ANALGESICS AND ANTIPYRETICS | 2.0 | 0.08 |
| 17 | G04B | UROLOGICALS | 2.0 | 0.08 |
| 18 | C10AX | Other lipid modifying agents | 3.0 | 0.08 |
| 19 | J07 | VACCINES | 1.0 | 0.07 |
| 20 | A03 | DRUGS FOR FUNCTIONAL GASTROINTESTINAL DISORDERS | 1.0 | 0.07 |
| 21 | A10BA02 | metformin | 4.0 | 0.07 |
| 22 | C03D | POTASSIUM-SPARING AGENTS | 2.0 | 0.07 |
| 23 | J01CA | Penicillins with extended spectrum | 3.0 | 0.07 |
| 24 | A02 | DRUGS FOR ACID RELATED DISORDERS | 1.0 | 0.07 |
| 25 | N03A | ANTIEPILEPTICS | 2.0 | 0.06 |
| 26 | A10A | INSULINS AND ANALOGUES | 2.0 | 0.06 |
| 27 | G04C | DRUGS USED IN BENIGN PROSTATIC HYPERTROPHY | 2.0 | 0.06 |
| 28 | N06 | PSYCHOANALEPTICS | 1.0 | 0.06 |
| 29 | C09B | ACE INHIBITORS, COMBINATIONS | 2.0 | 0.06 |
| 30 | D07A | CORTICOSTEROIDS, PLAIN | 2.0 | 0.05 |
| 31 | C07 | BETA BLOCKING AGENTS | 1.0 | 0.05 |
| 32 | L | ANTINEOPLASTIC AND IMMUNOMODULATING AGENTS | 0.0 | 0.05 |
| 33 | J01F | MACROLIDES, LINCOSAMIDES AND STREPTOGRAMINS | 2.0 | 0.05 |
| 34 | G03 | SEX HORMONES AND MODULATORS OF THE GENITAL SYSTEM | 1.0 | 0.05 |
| 35 | C03A | LOW-CEILING DIURETICS, THIAZIDES | 2.0 | 0.05 |
| 36 | A12 | MINERAL SUPPLEMENTS | 1.0 | 0.04 |
| 37 | C08C | SELECTIVE CALCIUM CHANNEL BLOCKERS WITH MAINLY VASCULAR EFFECTS | 2.0 | 0.04 |
| 38 | N02AA | Natural opium alkaloids | 3.0 | 0.04 |
| 39 | C07AG | Alpha and beta blocking agents | 3.0 | 0.04 |
| 40 | A04A | ANTIEMETICS AND ANTINAUSEANTS | 2.0 | 0.04 |
| 41 | C07AB02 | metoprolol | 4.0 | 0.04 |
| 42 | B01AC06 | acetylsalicylic acid | 4.0 | 0.04 |
| 43 | R01 | NASAL PREPARATIONS | 1.0 | 0.04 |
| 44 | C10AA01 | simvastatin | 4.0 | 0.03 |
| 45 | R03AK | Adrenergics in combination with corticosteroids or other drugs, excl. anticholinergics | 3.0 | 0.03 |
| 46 | R03B | OTHER DRUGS FOR OBSTRUCTIVE AIRWAY DISEASES, INHALANTS | 2.0 | 0.03 |
| 47 | C10AB | Fibrates | 3.0 | 0.03 |
| 48 | B01AC04 | clopidogrel | 4.0 | 0.03 |
| 49 | J01 | ANTIBACTERIALS FOR SYSTEMIC USE | 1.0 | 0.03 |
| 50 | C09AA04 | perindopril | 4.0 | 0.03 |
| 51 | C08D | SELECTIVE CALCIUM CHANNEL BLOCKERS WITH DIRECT CARDIAC EFFECTS | 2.0 | 0.03 |
| 52 | R03AC | Selective beta-2-adrenoreceptor agonists | 3.0 | 0.03 |
| 53 | N05C | HYPNOTICS AND SEDATIVES | 2.0 | 0.03 |
| 54 | S01B | ANTIINFLAMMATORY AGENTS | 2.0 | 0.03 |
| 55 | M03 | MUSCLE RELAXANTS | 1.0 | 0.03 |
| 56 | A10BG | Thiazolidinediones | 3.0 | 0.03 |
| 57 | B01AA | Vitamin K antagonists | 3.0 | 0.03 |
| 58 | H03 | THYROID THERAPY | 1.0 | 0.03 |
| 59 | J01CR | Combinations of penicillins, incl. beta-lactamase inhibitors | 3.0 | 0.02 |
| 60 | A10BB | Sulfonylureas | 3.0 | 0.02 |
| 61 | S02 | OTOLOGICALS | 1.0 | 0.02 |
| 62 | J01D | OTHER BETA-LACTAM ANTIBACTERIALS | 2.0 | 0.02 |
| 63 | A10BJ | Glucagon-like peptide-1 (GLP-1) analogues | 3.0 | 0.02 |
| 64 | M04 | ANTIGOUT PREPARATIONS | 1.0 | 0.02 |
| 65 | D | DERMATOLOGICALS | 0.0 | 0.02 |
| 66 | A07 | ANTIDIARRHEALS, INTESTINAL ANTIINFLAMMATORY/ANTIINFECTIVE AGENTS | 1.0 | 0.02 |
| 67 | N07 | OTHER NERVOUS SYSTEM DRUGS | 1.0 | 0.02 |
| 68 | A11 | VITAMINS | 1.0 | 0.02 |
| 69 | N06AB | Selective serotonin reuptake inhibitors | 3.0 | 0.02 |
| 70 | R05 | COUGH AND COLD PREPARATIONS | 1.0 | 0.02 |
| 71 | C01B | ANTIARRHYTHMICS, CLASS I AND III | 2.0 | 0.01 |
| 72 | D01 | ANTIFUNGALS FOR DERMATOLOGICAL USE | 1.0 | 0.01 |
| 73 | M02A | TOPICAL PRODUCTS FOR JOINT AND MUSCULAR PAIN | 2.0 | 0.01 |
| 74 | S | SENSORY ORGANS | 0.0 | 0.01 |
| 75 | M05B | DRUGS AFFECTING BONE STRUCTURE AND MINERALIZATION | 2.0 | 0.01 |
| 76 | N04 | ANTI-PARKINSON DRUGS | 1.0 | 0.01 |
| 77 | C07AA | Beta blocking agents, non-selective | 3.0 | 0.01 |
| 78 | N05B | ANXIOLYTICS | 2.0 | 0.01 |
| 79 | P | ANTIPARASITIC PRODUCTS, INSECTICIDES AND REPELLENTS | 0.0 | 0.01 |
| 80 | R06A | ANTIHISTAMINES FOR SYSTEMIC USE | 2.0 | 0.01 |
| 81 | A10BD | Combinations of oral blood glucose lowering drugs | 3.0 | 0.01 |
| 82 | C03BA | Sulfonamides, plain | 3.0 | 0.01 |
| 83 | C01A | CARDIAC GLYCOSIDES | 2.0 | 0.01 |
| 84 | A02BA | H2-receptor antagonists | 3.0 | 0.01 |
| 85 | A10BX | Other blood glucose lowering drugs, excl. insulins | 3.0 | 0.01 |
| 86 | C03EA | Low-ceiling diuretics and potassium-sparing agents | 3.0 | 0.01 |
| 87 | M01AE | Propionic acid derivatives | 3.0 | 0.01 |
| 88 | J01MA | Fluoroquinolones | 3.0 | 0.01 |
| 89 | S01E | ANTIGLAUCOMA PREPARATIONS AND MIOTICS | 2.0 | 0.01 |
| 90 | A10BH | Dipeptidyl peptidase 4 (DPP-4) inhibitors | 3.0 | 0.01 |
| 91 | J01A | TETRACYCLINES | 2.0 | 0.01 |
| 92 | A01A | STOMATOLOGICAL PREPARATIONS | 2.0 | 0.01 |
| 93 | D06 | ANTIBIOTICS AND CHEMOTHERAPEUTICS FOR DERMATOLOGICAL USE | 1.0 | 0.01 |
| 94 | C02 | ANTIHYPERTENSIVES | 1.0 | 0.01 |
| 95 | J05A | DIRECT ACTING ANTIVIRALS | 2.0 | 0.01 |
| 96 | J01X | OTHER ANTIBACTERIALS | 2.0 | 0.0 |
| 97 | J02 | ANTIMYCOTICS FOR SYSTEMIC USE | 1.0 | 0.0 |
| 98 | C05 | VASOPROTECTIVES | 1.0 | 0.0 |
| 99 | C04 | PERIPHERAL VASODILATORS | 1.0 | 0.0 |
|  |  |  |  |  |

**Table S15. Features Selected by AEFS in the PIN Dataset**

|  | **Code** | **Description** | **Rank** | **Mean Absolute SHAP Score** |
| --- | --- | --- | --- | --- |
| 0 | C03C | HIGH-CEILING DIURETICS | 2.0 | 0.5 |
| 1 | C03CA01 | furosemide | 4.0 | 0.06 |
| 2 | C09DA03 | valsartan and diuretics | 4.0 | 0.04 |
| 3 | C07AB02 | metoprolol | 4.0 | 0.03 |
| 4 | N06AB06 | sertraline | 4.0 | 0.03 |
| 5 | D07 | CORTICOSTEROIDS, DERMATOLOGICAL PREPARATIONS | 1.0 | 0.03 |
| 6 | M01AH | Coxibs | 3.0 | 0.02 |
| 7 | N02BA | Salicylic acid and derivatives | 3.0 | 0.02 |
| 8 | A01AD | Other agents for local oral treatment | 3.0 | 0.02 |
| 9 | C09AA03 | lisinopril | 4.0 | 0.02 |
| 10 | S02 | OTOLOGICALS | 1.0 | 0.02 |
| 11 | N07 | OTHER NERVOUS SYSTEM DRUGS | 1.0 | 0.02 |
| 12 | B05XA | Electrolyte solutions | 3.0 | 0.01 |
| 13 | M05BB01 | etidronic acid and calcium, sequential | 4.0 | 0.01 |
| 14 | D05A | ANTIPSORIATICS FOR TOPICAL USE | 2.0 | 0.01 |
| 15 | A01 | STOMATOLOGICAL PREPARATIONS | 1.0 | 0.01 |
| 16 | S01BA04 | prednisolone | 4.0 | 0.01 |
| 17 | S01AE07 | moxifloxacin | 4.0 | 0.01 |
| 18 | G03CA57 | conjugated estrogens | 4.0 | 0.01 |
| 19 | C08CA02 | felodipine | 4.0 | 0.01 |
| 20 | R03AL04 | indacaterol and glycopyrronium bromide | 4.0 | 0.0 |
| 21 | M03BX01 | baclofen | 4.0 | 0.0 |
| 22 | J01CF02 | cloxacillin | 4.0 | 0.0 |
| 23 | V03AF03 | calcium folinate | 4.0 | 0.0 |
| 24 | A05AA | Bile acids and derivatives | 3.0 | 0.0 |
| 25 | C02AC01 | clonidine | 4.0 | 0.0 |
| 26 | S01A | ANTIINFECTIVES | 2.0 | 0.0 |
| 27 | A10AE06 | insulin degludec | 4.0 | 0.0 |
| 28 | L01BA01 | methotrexate | 4.0 | 0.0 |
| 29 | C09XA02 | aliskiren | 4.0 | 0.0 |
| 30 | G01A | ANTIINFECTIVES AND ANTISEPTICS, EXCL. COMBINATIONS WITH CORTICOSTEROIDS | 2.0 | 0.0 |
| 31 | R03BA08 | ciclesonide | 4.0 | 0.0 |
| 32 | N03AA | Barbiturates and derivatives | 3.0 | 0.0 |
| 33 | C01BB | Antiarrhythmics, class Ib | 3.0 | 0.0 |
| 34 | R05DA09 | dextromethorphan | 4.0 | 0.0 |
| 35 | S02BA08 | fluocinolone acetonide | 4.0 | 0.0 |
| 36 | C09DA02 | eprosartan and diuretics | 4.0 | 0.0 |
| 37 | R03AC03 | terbutaline | 4.0 | 0.0 |
| 38 | N05BE | Azaspirodecanedione derivatives | 3.0 | 0.0 |
| 39 | L01D | CYTOTOXIC ANTIBIOTICS AND RELATED SUBSTANCES | 2.0 | 0.0 |
| 40 | J02AB02 | ketoconazole | 4.0 | 0.0 |
| 41 | C03DA04 | eplerenone | 4.0 | 0.0 |
| 42 | B03AD | Iron in combination with folic acid | 3.0 | 0.0 |
| 43 | L01XE01 | IMATINIB | 4.0 | 0.0 |
| 44 | H04A | GLYCOGENOLYTIC HORMONES | 2.0 | 0.0 |
| 45 | C02CA04 | doxazosin | 4.0 | 0.0 |
| 46 | J05AB14 | valganciclovir | 4.0 | 0.0 |
| 47 | P02CC | Tetrahydropyrimidine derivatives | 3.0 | 0.0 |
| 48 | N05BA04 | oxazepam | 4.0 | 0.0 |
| 49 | R03AC13 | formoterol | 4.0 | 0.0 |
| 50 | R02A | THROAT PREPARATIONS | 2.0 | 0.0 |
| 51 | S01FA05 | homatropine | 4.0 | 0.0 |
| 52 | N02AA02 | opium | 4.0 | 0.0 |
| 53 | L04AA29 | tofacitinib | 4.0 | 0.0 |
| 54 | S01EB | Parasympathomimetics | 3.0 | 0.0 |
| 55 | H01BA | Vasopressin and analogues | 3.0 | 0.0 |
| 56 | J05AE | Protease inhibitors | 3.0 | 0.0 |
| 57 | N02BG10 | cannabinoids | 4.0 | 0.0 |
| 58 | M03BC | Ethers, chemically close to antihistamines | 3.0 | 0.0 |
| 59 | S01BA05 | triamcinolone | 4.0 | 0.0 |
| 60 | R02AA | Antiseptics | 3.0 | 0.0 |
| 61 | G03G | GONADOTROPINS AND OTHER OVULATION STIMULANTS | 2.0 | 0.0 |
| 62 | L01XX45 | CARFILZOMIB | 4.0 | 0.0 |
| 63 | C10B | LIPID MODIFYING AGENTS, COMBINATIONS | 2.0 | 0.0 |
| 64 | A07F | ANTIDIARRHEAL MICROORGANISMS | 2.0 | 0.0 |
| 65 | A16A | OTHER ALIMENTARY TRACT AND METABOLISM PRODUCTS | 2.0 | 0.0 |
| 66 | D02B | PROTECTIVES AGAINST UV-RADIATION | 2.0 | 0.0 |
| 67 | J04AK02 | ethambutol | 4.0 | 0.0 |
| 68 | L01XG01 | bortezomib | 4.0 | 0.0 |
| 69 | A10BB03 | tolbutamide | 4.0 | 0.0 |
| 70 | N02BG04 | floctafenine | 4.0 | 0.0 |
| 71 | N02CA52 | ergotamine, combinations excl. psycholeptics | 4.0 | 0.0 |
| 72 | L01AX04 | dacarbazine | 4.0 | 0.0 |
| 73 | J07BG01 | rabies, inactivated, whole virus | 4.0 | 0.0 |
| 74 | J05AE04 | nelfinavir | 4.0 | 0.0 |
| 75 | J05AG05 | rilpivirine | 4.0 | 0.0 |
| 76 | S01AB04 | sulfacetamide | 4.0 | 0.0 |
| 77 | N05AC04 | pipotiazine | 4.0 | 0.0 |
| 78 | L01XE13 | AFATINIB | 4.0 | 0.0 |
| 79 | D05BA02 | methoxsalen | 4.0 | 0.0 |
| 80 | R06AD01 | alimemazine | 4.0 | 0.0 |
| 81 | M02AX03 | dimethyl sulfoxide | 4.0 | 0.0 |
| 82 | A07EB | Antiallergic agents, excl. corticosteroids | 3.0 | 0.0 |
| 83 | D08AG53 | IODINE, COMBINATIONS | 4.0 | 0.0 |
| 84 | A09AC | Enzyme and acid preparations, combinations | 3.0 | 0.0 |
| 85 | P03AB | Chlorine containing products | 3.0 | 0.0 |
| 86 | P01CX01 | pentamidine isethionate | 4.0 | 0.0 |
| 87 | B05XA01 | potassium chloride | 4.0 | 0.0 |
| 88 | L01XE26 | CABOZANTINIB | 4.0 | 0.0 |
| 89 | L01EA04 | bosutinib | 4.0 | 0.0 |
| 90 | A01AB12 | hexetidine | 4.0 | 0.0 |
| 91 | G03DC02 | norethisterone | 4.0 | 0.0 |
| 92 | M03BC01 | orphenadrine (citrate) | 4.0 | 0.0 |
|  |  |  |  |  |

**Table S16. Features Selected by MCFS in the PIN Dataset**

|  | **Code** | **Description** | **Rank** | **Mean Absolute SHAP Score** |
| --- | --- | --- | --- | --- |
| 0 | C03C | HIGH-CEILING DIURETICS | 2.0 | 0.41 |
| 1 | B | BLOOD AND BLOOD FORMING ORGANS | 0.0 | 0.22 |
| 2 | C10 | LIPID MODIFYING AGENTS | 1.0 | 0.19 |
| 3 | N | NERVOUS SYSTEM | 0.0 | 0.17 |
| 4 | A02BC02 | pantoprazole | 4.0 | 0.15 |
| 5 | N02 | ANALGESICS | 1.0 | 0.15 |
| 6 | M01 | ANTIINFLAMMATORY AND ANTIRHEUMATIC PRODUCTS | 1.0 | 0.14 |
| 7 | C09A | ACE INHIBITORS, PLAIN | 2.0 | 0.12 |
| 8 | A06 | DRUGS FOR CONSTIPATION | 1.0 | 0.11 |
| 9 | C09C | ANGIOTENSIN II RECEPTOR BLOCKERS (ARBs), PLAIN | 2.0 | 0.11 |
| 10 | N02BE01 | paracetamol | 4.0 | 0.09 |
| 11 | J | ANTIINFECTIVES FOR SYSTEMIC USE | 0.0 | 0.08 |
| 12 | H02 | CORTICOSTEROIDS FOR SYSTEMIC USE | 1.0 | 0.08 |
| 13 | A10BA | Biguanides | 3.0 | 0.07 |
| 14 | A03 | DRUGS FOR FUNCTIONAL GASTROINTESTINAL DISORDERS | 1.0 | 0.07 |
| 15 | B01AC | Platelet aggregation inhibitors excl. heparin | 3.0 | 0.07 |
| 16 | C08 | CALCIUM CHANNEL BLOCKERS | 1.0 | 0.07 |
| 17 | B03 | ANTIANEMIC PREPARATIONS | 1.0 | 0.06 |
| 18 | R03BB | Anticholinergics | 3.0 | 0.06 |
| 19 | C07 | BETA BLOCKING AGENTS | 1.0 | 0.06 |
| 20 | C01D | VASODILATORS USED IN CARDIAC DISEASES | 2.0 | 0.06 |
| 21 | C10AA | HMG CoA reductase inhibitors | 3.0 | 0.06 |
| 22 | G04C | DRUGS USED IN BENIGN PROSTATIC HYPERTROPHY | 2.0 | 0.06 |
| 23 | D | DERMATOLOGICALS | 0.0 | 0.05 |
| 24 | B01 | ANTITHROMBOTIC AGENTS | 1.0 | 0.05 |
| 25 | G | GENITO URINARY SYSTEM AND SEX HORMONES | 0.0 | 0.05 |
| 26 | A02B | DRUGS FOR PEPTIC ULCER AND GASTRO-OESOPHAGEAL REFLUX DISEASE (GORD) | 2.0 | 0.05 |
| 27 | N02A | OPIOIDS | 2.0 | 0.05 |
| 28 | L | ANTINEOPLASTIC AND IMMUNOMODULATING AGENTS | 0.0 | 0.05 |
| 29 | N02BE51 | paracetamol, combinations excl. psycholeptics | 4.0 | 0.04 |
| 30 | A12 | MINERAL SUPPLEMENTS | 1.0 | 0.04 |
| 31 | H | SYSTEMIC HORMONAL PREPARATIONS, EXCL. SEX HORMONES AND INSULINS | 0.0 | 0.04 |
| 32 | A04 | ANTIEMETICS AND ANTINAUSEANTS | 1.0 | 0.03 |
| 33 | R | RESPIRATORY SYSTEM | 0.0 | 0.03 |
| 34 | C10AA07 | rosuvastatin | 4.0 | 0.03 |
| 35 | J01D | OTHER BETA-LACTAM ANTIBACTERIALS | 2.0 | 0.03 |
| 36 | C08CA01 | amlodipine | 4.0 | 0.03 |
| 37 | A10A | INSULINS AND ANALOGUES | 2.0 | 0.03 |
| 38 | C | CARDIOVASCULAR SYSTEM | 0.0 | 0.03 |
| 39 | C03AA | Thiazides, plain | 3.0 | 0.03 |
| 40 | C03CA01 | furosemide | 4.0 | 0.03 |
| 41 | N03AX | Other antiepileptics | 3.0 | 0.03 |
| 42 | G03 | SEX HORMONES AND MODULATORS OF THE GENITAL SYSTEM | 1.0 | 0.03 |
| 43 | C03DA | Aldosterone antagonists | 3.0 | 0.03 |
| 44 | C09DA04 | irbesartan and diuretics | 4.0 | 0.03 |
| 45 | A | ALIMENTARY TRACT AND METABOLISM | 0.0 | 0.03 |
| 46 | A10 | DRUGS USED IN DIABETES | 1.0 | 0.02 |
| 47 | C03DA01 | spironolactone | 4.0 | 0.02 |
| 48 | J01CR02 | amoxicillin and beta-lactamase inhibitor | 4.0 | 0.02 |
| 49 | J01FA09 | clarithromycin | 4.0 | 0.02 |
| 50 | J01M | QUINOLONE ANTIBACTERIALS | 2.0 | 0.02 |
| 51 | J01 | ANTIBACTERIALS FOR SYSTEMIC USE | 1.0 | 0.02 |
| 52 | G04CA | Alpha-adrenoreceptor antagonists | 3.0 | 0.02 |
| 53 | B01AC04 | clopidogrel | 4.0 | 0.01 |
| 54 | N05C | HYPNOTICS AND SEDATIVES | 2.0 | 0.01 |
| 55 | A10B | BLOOD GLUCOSE LOWERING DRUGS, EXCL. INSULINS | 2.0 | 0.01 |
| 56 | S | SENSORY ORGANS | 0.0 | 0.01 |
| 57 | N02AJ | Opioids in combination with non-opioid analgesics | 3.0 | 0.01 |
| 58 | A10BK | Sodium-glucose co-transporter 2 (SGLT2) inhibitors | 3.0 | 0.01 |
| 59 | M04AA01 | allopurinol | 4.0 | 0.01 |
| 60 | C03CA | Sulfonamides, plain | 3.0 | 0.01 |
| 61 | C03D | POTASSIUM-SPARING AGENTS | 2.0 | 0.01 |
| 62 | N03AX12 | gabapentin | 4.0 | 0.01 |
| 63 | J01A | TETRACYCLINES | 2.0 | 0.01 |
| 64 | H02AB07 | prednisone | 4.0 | 0.01 |
| 65 | M05 | DRUGS FOR TREATMENT OF BONE DISEASES | 1.0 | 0.01 |
| 66 | H03AA01 | levothyroxine sodium | 4.0 | 0.01 |
| 67 | C01DA02 | glyceryl trinitrate | 4.0 | 0.01 |
| 68 | H03 | THYROID THERAPY | 1.0 | 0.01 |
| 69 | D06A | ANTIBIOTICS FOR TOPICAL USE | 2.0 | 0.01 |
| 70 | B03BA | Vitamin B12 (cyanocobalamin and analogues) | 3.0 | 0.0 |
| 71 | M | MUSCULO-SKELETAL SYSTEM | 0.0 | 0.0 |
| 72 | N02BA01 | acetylsalicylic acid | 4.0 | 0.0 |
| 73 | C09CA06 | candesartan | 4.0 | 0.0 |
| 74 | C10AA05 | atorvastatin | 4.0 | 0.0 |
| 75 | H03AA05 | thyroid gland preparations | 4.0 | 0.0 |
| 76 | M04 | ANTIGOUT PREPARATIONS | 1.0 | 0.0 |
| 77 | C09CA04 | irbesartan | 4.0 | 0.0 |
| 78 | N02B | OTHER ANALGESICS AND ANTIPYRETICS | 2.0 | 0.0 |
| 79 | N05B | ANXIOLYTICS | 2.0 | 0.0 |
| 80 | H03A | THYROID PREPARATIONS | 2.0 | 0.0 |
| 81 | B03BA01 | cyanocobalamin | 4.0 | 0.0 |
| 82 | A02 | DRUGS FOR ACID RELATED DISORDERS | 1.0 | 0.0 |
| 83 | C09CA03 | valsartan | 4.0 | 0.0 |
| 84 | C01DA14 | isosorbide mononitrate | 4.0 | 0.0 |
| 85 | M01A | ANTIINFLAMMATORY AND ANTIRHEUMATIC PRODUCTS, NON-STEROIDS | 2.0 | 0.0 |
| 86 | H03B | ANTITHYROID PREPARATIONS | 2.0 | 0.0 |
| 87 | C10A | LIPID MODIFYING AGENTS, PLAIN | 2.0 | 0.0 |
| 88 | M04AA03 | febuxostat | 4.0 | 0.0 |
| 89 | M01C | SPECIFIC ANTIRHEUMATIC AGENTS | 2.0 | 0.0 |
| 90 | C10B | LIPID MODIFYING AGENTS, COMBINATIONS | 2.0 | 0.0 |
| 91 | C03CA02 | bumetanide | 4.0 | 0.0 |
| 92 | H03AA | Thyroid hormones | 3.0 | 0.0 |
| 93 | B01A | ANTITHROMBOTIC AGENTS | 2.0 | 0.0 |
| 94 | C09CA | Angiotensin II receptor blockers (ARBs), plain | 3.0 | 0.0 |
| 95 | M04A | ANTIGOUT PREPARATIONS | 2.0 | 0.0 |
| 96 | C03AA03 | hydrochlorothiazide | 4.0 | 0.0 |
| 97 | A10BA02 | metformin | 4.0 | 0.0 |
| 98 | A06A | DRUGS FOR CONSTIPATION | 2.0 | 0.0 |
| 99 | C01DA | Organic nitrates | 3.0 | 0.0 |
|  |  |  |  |  |

**Table S17. Features Selected by PFA in the PIN Dataset**

|  | **Code** | **Description** | **Rank** | **Mean Absolute SHAP Score** |
| --- | --- | --- | --- | --- |
| 0 | N04B | DOPAMINERGIC AGENTS | 2.0 | 0.03 |
| 1 | C09DX04 | valsartan and sacubitril | 4.0 | 0.02 |
| 2 | D01AA | Antibiotics | 3.0 | 0.01 |
| 3 | C05AA | Corticosteroids | 3.0 | 0.01 |
| 4 | R03BB05 | aclidinium bromide | 4.0 | 0.01 |
| 5 | N02AE | Oripavine derivatives | 3.0 | 0.01 |
| 6 | J01CF | Beta-lactamase resistant penicillins | 3.0 | 0.01 |
| 7 | M05BB | Bisphosphonates, combinations | 3.0 | 0.0 |
| 8 | A05AA | Bile acids and derivatives | 3.0 | 0.0 |
| 9 | C01DA08 | isosorbide dinitrate | 4.0 | 0.0 |
| 10 | M05BX | Other drugs affecting bone structure and mineralization | 3.0 | 0.0 |
| 11 | A06AA01 | liquid paraffin | 4.0 | 0.0 |
| 12 | C05AX | Other agents for treatment of hemorrhoids and anal fissures for topical use | 3.0 | 0.0 |
| 13 | C07CB | Beta blocking agents, selective, and other diuretics | 3.0 | 0.0 |
| 14 | B01AX | Other antithrombotic agents | 3.0 | 0.0 |
| 15 | N05AH01 | loxapine | 4.0 | 0.0 |
| 16 | C09BB04 | perindopril and amlodipine | 4.0 | 0.0 |
| 17 | C07AG01 | labetalol | 4.0 | 0.0 |
| 18 | B01AC22 | prasugrel | 4.0 | 0.0 |
| 19 | J01XA | Glycopeptide antibacterials | 3.0 | 0.0 |
| 20 | C10AB04 | gemfibrozil | 4.0 | 0.0 |
| 21 | S02BA08 | fluocinolone acetonide | 4.0 | 0.0 |
| 22 | J01FA | Macrolides | 3.0 | 0.0 |
| 23 | N05CD02 | nitrazepam | 4.0 | 0.0 |
| 24 | A10BX12 | EMPAGLIFLOZIN | 4.0 | 0.0 |
| 25 | D03B | ENZYMES | 2.0 | 0.0 |
| 26 | H04A | GLYCOGENOLYTIC HORMONES | 2.0 | 0.0 |
| 27 | M03BC01 | orphenadrine (citrate) | 4.0 | 0.0 |
| 28 | N05AG02 | pimozide | 4.0 | 0.0 |
| 29 | D07AC21 | ulobetasol | 4.0 | 0.0 |
| 30 | L01XE05 | SORAFENIB | 4.0 | 0.0 |
| 31 | A12AX | Calcium, combinations with vitamin D and/or other drugs | 3.0 | 0.0 |
| 32 | D06AX07 | gentamicin | 4.0 | 0.0 |
| 33 | D07AC06 | diflucortolone | 4.0 | 0.0 |
| 34 | S01EB01 | pilocarpine | 4.0 | 0.0 |
| 35 | H02AB06 | prednisolone | 4.0 | 0.0 |
| 36 | R01AX30 | combinations | 4.0 | 0.0 |
| 37 | A07FA01 | lactic acid producing organisms | 4.0 | 0.0 |
| 38 | A13 | TONICS | 1.0 | 0.0 |
| 39 | A06AD04 | magnesium sulfate | 4.0 | 0.0 |
| 40 | A07BC04 | attapulgite | 4.0 | 0.0 |
| 41 | V03AE03 | lanthanum carbonate | 4.0 | 0.0 |
| 42 | A02BD07 | lansoprazole, amoxicillin and clarithromycin | 4.0 | 0.0 |
| 43 | N05BA04 | oxazepam | 4.0 | 0.0 |
| 44 | L01XX41 | eribulin | 4.0 | 0.0 |
| 45 | L01XX47 | IDELALISIB | 4.0 | 0.0 |
| 46 | L03AC01 | aldesleukin | 4.0 | 0.0 |
| 47 | L04AA26 | belimumab | 4.0 | 0.0 |
| 48 | L04AC12 | brodalumab | 4.0 | 0.0 |
| 49 | M01AE11 | tiaprofenic acid | 4.0 | 0.0 |
| 50 | M01AX | Other antiinflammatory and antirheumatic agents, non-steroids | 3.0 | 0.0 |
| 51 | S01AD | Antivirals | 3.0 | 0.0 |
| 52 | N05BA05 | potassium clorazepate | 4.0 | 0.0 |
| 53 | R06AB04 | chlorphenamine | 4.0 | 0.0 |
| 54 | N02AD | Benzomorphan derivatives | 3.0 | 0.0 |
| 55 | R05DA03 | hydrocodone | 4.0 | 0.0 |
| 56 | R03DA05 | aminophylline | 4.0 | 0.0 |
| 57 | R01BA53 | phenylephrine, combinations | 4.0 | 0.0 |
| 58 | P03AB | Chlorine containing products | 3.0 | 0.0 |
| 59 | N04BA03 | levodopa, decarboxylase inhibitor and COMT inhibitor | 4.0 | 0.0 |
| 60 | P01BD01 | pyrimethamine | 4.0 | 0.0 |
| 61 | N06CA01 | amitriptyline and psycholeptics | 4.0 | 0.0 |
| 62 | N06AA06 | trimipramine | 4.0 | 0.0 |
| 63 | L01XE16 | Crizotinib | 4.0 | 0.0 |
| 64 | A02AB01 | aluminium hydroxide | 4.0 | 0.0 |
| 65 | L01XC32 | atezolizumab | 4.0 | 0.0 |
| 66 | A12AA20 | calcium (different salts in combination) | 4.0 | 0.0 |
| 67 | C04AA02 | buphenine | 4.0 | 0.0 |
| 68 | C03DB | Other potassium-sparing agents | 3.0 | 0.0 |
| 69 | B05Z | HEMODIALYTICS AND HEMOFILTRATES | 2.0 | 0.0 |
| 70 | B03XA06 | luspatercept | 4.0 | 0.0 |
| 71 | B03BB51 | folic acid, combinations | 4.0 | 0.0 |
| 72 | B03BA51 | cyanocobalamin, combinations | 4.0 | 0.0 |
| 73 | A11HA01 | nicotinamide | 4.0 | 0.0 |
| 74 | C08CA04 | nicardipine | 4.0 | 0.0 |
| 75 | A11CB | Vitamin A and D in combination | 3.0 | 0.0 |
| 76 | A10BD19 | linagliptin and empagliflozin | 4.0 | 0.0 |
| 77 | A10AE01 | insulin (human) | 4.0 | 0.0 |
| 78 | A08AA01 | phentermine | 4.0 | 0.0 |
| 79 | A06AD17 | sodium phosphate | 4.0 | 0.0 |
| 80 | A06AB57 | cascara, combinations | 4.0 | 0.0 |
| 81 | C05AX03 | other preparations, combinations | 4.0 | 0.0 |
| 82 | C08CA06 | nimodipine | 4.0 | 0.0 |
| 83 | L01XC06 | cetuximab | 4.0 | 0.0 |
| 84 | H03BA02 | propylthiouracil | 4.0 | 0.0 |
| 85 | J07AH09 | meningococcus B, multicomponent vaccine | 4.0 | 0.0 |
| 86 | J01G | AMINOGLYCOSIDE ANTIBACTERIALS | 2.0 | 0.0 |
| 87 | J01FF02 | lincomycin | 4.0 | 0.0 |
| 88 | J01DE | Fourth-generation cephalosporins | 3.0 | 0.0 |
| 89 | J01DD01 | cefotaxime | 4.0 | 0.0 |
| 90 | J01DC04 | cefaclor | 4.0 | 0.0 |
| 91 | A02BB01 | misoprostol | 4.0 | 0.0 |
| 92 | C08CA55 | nifedipine, combinations | 4.0 | 0.0 |
| 93 | G03DC02 | norethisterone | 4.0 | 0.0 |
| 94 | G01AF15 | butoconazole | 4.0 | 0.0 |
| 95 | G01AC30 | oxyquinoline | 4.0 | 0.0 |
| 96 | D10AB | Preparations containing sulfur | 3.0 | 0.0 |
| 97 | D02B | PROTECTIVES AGAINST UV-RADIATION | 2.0 | 0.0 |
| 98 | D01AC02 | miconazole | 4.0 | 0.0 |
| 99 | V04 | DIAGNOSTIC AGENTS | 1.0 | 0.0 |
|  |  |  |  |  |

**Table S18. Features Selected by LS in the PIN Dataset**

|  | **Code** | **Description** | **Rank** | **Mean Absolute SHAP Score** |
| --- | --- | --- | --- | --- |
| 0 | C03C | HIGH-CEILING DIURETICS | 2.0 | 0.38 |
| 1 | B | BLOOD AND BLOOD FORMING ORGANS | 0.0 | 0.31 |
| 2 | N | NERVOUS SYSTEM | 0.0 | 0.3 |
| 3 | C09 | AGENTS ACTING ON THE RENIN-ANGIOTENSIN SYSTEM | 1.0 | 0.28 |
| 4 | A | ALIMENTARY TRACT AND METABOLISM | 0.0 | 0.25 |
| 5 | C10 | LIPID MODIFYING AGENTS | 1.0 | 0.24 |
| 6 | H | SYSTEMIC HORMONAL PREPARATIONS, EXCL. SEX HORMONES AND INSULINS | 0.0 | 0.21 |
| 7 | A02BC02 | pantoprazole | 4.0 | 0.18 |
| 8 | N05 | PSYCHOLEPTICS | 1.0 | 0.11 |
| 9 | C07A | BETA BLOCKING AGENTS | 2.0 | 0.09 |
| 10 | G04BE | Drugs used in erectile dysfunction | 3.0 | 0.09 |
| 11 | A02B | DRUGS FOR PEPTIC ULCER AND GASTRO-OESOPHAGEAL REFLUX DISEASE (GORD) | 2.0 | 0.09 |
| 12 | A10BA | Biguanides | 3.0 | 0.09 |
| 13 | B01AC | Platelet aggregation inhibitors excl. heparin | 3.0 | 0.08 |
| 14 | C01D | VASODILATORS USED IN CARDIAC DISEASES | 2.0 | 0.07 |
| 15 | H03A | THYROID PREPARATIONS | 2.0 | 0.07 |
| 16 | C03DA | Aldosterone antagonists | 3.0 | 0.06 |
| 17 | C07 | BETA BLOCKING AGENTS | 1.0 | 0.06 |
| 18 | C09DA | Angiotensin II receptor blockers (ARBs) and diuretics | 3.0 | 0.06 |
| 19 | G | GENITO URINARY SYSTEM AND SEX HORMONES | 0.0 | 0.05 |
| 20 | C03CA01 | furosemide | 4.0 | 0.05 |
| 21 | C07AB02 | metoprolol | 4.0 | 0.05 |
| 22 | G04 | UROLOGICALS | 1.0 | 0.05 |
| 23 | C07AB07 | bisoprolol | 4.0 | 0.05 |
| 24 | C07AG02 | carvedilol | 4.0 | 0.05 |
| 25 | A02BC | Proton pump inhibitors | 3.0 | 0.04 |
| 26 | C10AA07 | rosuvastatin | 4.0 | 0.04 |
| 27 | C08 | CALCIUM CHANNEL BLOCKERS | 1.0 | 0.04 |
| 28 | C08CA01 | amlodipine | 4.0 | 0.04 |
| 29 | G04CA02 | tamsulosin | 4.0 | 0.03 |
| 30 | C10AA | HMG CoA reductase inhibitors | 3.0 | 0.03 |
| 31 | A10B | BLOOD GLUCOSE LOWERING DRUGS, EXCL. INSULINS | 2.0 | 0.03 |
| 32 | C03A | LOW-CEILING DIURETICS, THIAZIDES | 2.0 | 0.03 |
| 33 | N05CF | Benzodiazepine related drugs | 3.0 | 0.03 |
| 34 | C09AA04 | perindopril | 4.0 | 0.03 |
| 35 | A10A | INSULINS AND ANALOGUES | 2.0 | 0.03 |
| 36 | C09C | ANGIOTENSIN II RECEPTOR BLOCKERS (ARBs), PLAIN | 2.0 | 0.03 |
| 37 | N06 | PSYCHOANALEPTICS | 1.0 | 0.03 |
| 38 | G04C | DRUGS USED IN BENIGN PROSTATIC HYPERTROPHY | 2.0 | 0.02 |
| 39 | C09B | ACE INHIBITORS, COMBINATIONS | 2.0 | 0.02 |
| 40 | B01AC06 | acetylsalicylic acid | 4.0 | 0.02 |
| 41 | B01 | ANTITHROMBOTIC AGENTS | 1.0 | 0.02 |
| 42 | C03 | DIURETICS | 1.0 | 0.02 |
| 43 | A10 | DRUGS USED IN DIABETES | 1.0 | 0.02 |
| 44 | C09AA05 | ramipril | 4.0 | 0.02 |
| 45 | C09A | ACE INHIBITORS, PLAIN | 2.0 | 0.01 |
| 46 | L01BA01 | methotrexate | 4.0 | 0.01 |
| 47 | A02 | DRUGS FOR ACID RELATED DISORDERS | 1.0 | 0.01 |
| 48 | C08DB01 | diltiazem | 4.0 | 0.01 |
| 49 | H03 | THYROID THERAPY | 1.0 | 0.01 |
| 50 | C10AA05 | atorvastatin | 4.0 | 0.01 |
| 51 | N05CF01 | zopiclone | 4.0 | 0.01 |
| 52 | M04AA01 | allopurinol | 4.0 | 0.01 |
| 53 | L01BA | Folic acid analogues | 3.0 | 0.0 |
| 54 | C03D | POTASSIUM-SPARING AGENTS | 2.0 | 0.0 |
| 55 | C | CARDIOVASCULAR SYSTEM | 0.0 | 0.0 |
| 56 | C03CA | Sulfonamides, plain | 3.0 | 0.0 |
| 57 | L04A | IMMUNOSUPPRESSANTS | 2.0 | 0.0 |
| 58 | C09DX | Angiotensin II receptor blockers (ARBs), other combinations | 3.0 | 0.0 |
| 59 | N06A | ANTIDEPRESSANTS | 2.0 | 0.0 |
| 60 | C07AB | Beta blocking agents, selective | 3.0 | 0.0 |
| 61 | N05C | HYPNOTICS AND SEDATIVES | 2.0 | 0.0 |
| 62 | M04 | ANTIGOUT PREPARATIONS | 1.0 | 0.0 |
| 63 | M04AA | Preparations inhibiting uric acid production | 3.0 | 0.0 |
| 64 | L04AD | Calcineurin inhibitors | 3.0 | 0.0 |
| 65 | C07AG | Alpha and beta blocking agents | 3.0 | 0.0 |
| 66 | L04AD02 | tacrolimus | 4.0 | 0.0 |
| 67 | P01BA02 | hydroxychloroquine | 4.0 | 0.0 |
| 68 | N05AB03 | perphenazine | 4.0 | 0.0 |
| 69 | L04 | IMMUNOSUPPRESSANTS | 1.0 | 0.0 |
| 70 | C09D | ANGIOTENSIN II RECEPTOR BLOCKERS (ARBs), COMBINATIONS | 2.0 | 0.0 |
| 71 | L04AA | Selective immunosuppressants | 3.0 | 0.0 |
| 72 | P01BA | Aminoquinolines | 3.0 | 0.0 |
| 73 | L04AA06 | mycophenolic acid | 4.0 | 0.0 |
| 74 | N06AF04 | tranylcypromine | 4.0 | 0.0 |
| 75 | B01AA07 | acenocoumarol | 4.0 | 0.0 |
| 76 | N07XX04 | sodium oxybate | 4.0 | 0.0 |
| 77 | N03AG04 | vigabatrin | 4.0 | 0.0 |
| 78 | M04A | ANTIGOUT PREPARATIONS | 2.0 | 0.0 |
| 79 | C09CA | Angiotensin II receptor blockers (ARBs), plain | 3.0 | 0.0 |
| 80 | L04AA31 | teriflunomide | 4.0 | 0.0 |
| 81 | L04AA27 | fingolimod | 4.0 | 0.0 |
| 82 | A10BA02 | metformin | 4.0 | 0.0 |
| 83 | B01A | ANTITHROMBOTIC AGENTS | 2.0 | 0.0 |
| 84 | B01AC21 | treprostinil | 4.0 | 0.0 |
| 85 | C01DA | Organic nitrates | 3.0 | 0.0 |
| 86 | C02KX04 | macitentan | 4.0 | 0.0 |
| 87 | C03AA | Thiazides, plain | 3.0 | 0.0 |
| 88 | C03AA03 | hydrochlorothiazide | 4.0 | 0.0 |
| 89 | C08C | SELECTIVE CALCIUM CHANNEL BLOCKERS WITH MAINLY VASCULAR EFFECTS | 2.0 | 0.0 |
| 90 | C08CA | Dihydropyridine derivatives | 3.0 | 0.0 |
| 91 | C09AA | ACE inhibitors, plain | 3.0 | 0.0 |
| 92 | C09BA | ACE inhibitors and diuretics | 3.0 | 0.0 |
| 93 | C09DX04 | valsartan and sacubitril | 4.0 | 0.0 |
| 94 | C10A | LIPID MODIFYING AGENTS, PLAIN | 2.0 | 0.0 |
| 95 | G04CA | Alpha-adrenoreceptor antagonists | 3.0 | 0.0 |
| 96 | H03AA | Thyroid hormones | 3.0 | 0.0 |
| 97 | H03AA01 | levothyroxine sodium | 4.0 | 0.0 |
| 98 | J05AF13 | tenofovir alafenamide | 4.0 | 0.0 |
| 99 | R02AA20 | various | 4.0 | 0.0 |
|  |  |  |  |  |
